# Synthesis of novel cytotoxic tetracyclic acridone derivatives and study of their molecular docking, ADMET, QSAR, bioactivity and protein binding properties

**DOI:** 10.1038/s41598-020-77590-1

**Published:** 2020-11-26

**Authors:** Rajkumar Veligeti, Rajesh Bagepalli Madhu, Jayashree Anireddy, Visweswara Rao Pasupuleti, Vijaya Kumar Reddy Avula, Krishna S. Ethiraj, Srinivas Uppalanchi, Sivaprasad Kasturi, Yogeeswari Perumal, Hasitha Shilpa Anantaraju, Naveen Polkam, Mallilkarjuna Reddy Guda, Swetha Vallela, Grigory Vasilievich Zyryanov

**Affiliations:** 1 Centre for Chemical Sciences and Technology, Institute of Science and Technology, Jawaharlal Nehru Technological University Hyderabad, Hyderabad, Telangana 500085 India; 2 Medicinal Chemistry Division, GVK Biosciences Private Limited, Plot No. 28A, IDA Nacharam, Hyderabad, Telangana 500076 India; 3 Discovery and Development Solutions, GVK Biosciences Private Limited, Plot No. 284A, Jigini Village, Bengaluru, Karnataka 562106 India; 4 Department of Biomedical Sciences and Therapeutics, Faculty of Medicine and Health Sciences, Universiti Malaysia Sabah, 88400 Kota Kinabalu, Sabah Malaysia; 5 Chemical Engineering Institute, Ural Federal University, Yekaterinburg, Russian Federation 620002; 6 Drug Discovery Research Laboratory, Department of Pharmacy, Birla Institute of Technology and Science - Pilani, Hyderabad Campus, Hyderabad, Telangana 500078 India; 7 Ural Division of the Russian Academy of Sciences, I. Ya. Postovskiy Institute of Organic Synthesis, 22 S. Kovalevskoy Street, Yekaterinburg, Russian Federation 620219

**Keywords:** Medicinal chemistry, Organic chemistry, Chemical synthesis

## Abstract

Acridone based synthetic and natural products with inherent anticancer activity advancing the research and generating a large number of structurally diversified compounds. In this sequence we have designed, synthesized a series of tetracyclic acridones with amide framework viz*.*, 3-(alkyloyl/ aryloyl/ heteroaryloyl/ heteroaryl)-2,3-dihydropyrazino[3,2,1-*de*]acridin-7(1*H*)-ones and screened for their in vitro anti-cancer activity. The in vitro study revealed that compounds with cyclopropyl-acetyl, benzoyl, *p*-hydroxybenzoyl, *p*-**(trifluoromethyl)benzoyl**, *p*-fluorobenzoyl, *m*-fluorobenzoyl, picolinoyl, 6-methylpicolinoyl and 3-nicotinoyl groups are active against HT29, MDAMB231 and HEK293T cancer cell lines. The molecular docking studies performed for them against 4N5Y, HT29 and 2VWD revealed the potential ligand–protein binding interactions among the neutral aminoacid of the enzymes and carbonyl groups of the title compounds with a binding energy ranging from − 8.1394 to − 6.9915 kcal/mol. In addition, the BSA protein binding assay performed for them has confirmed their interaction with target proteins through strong binding to BSA macromolecule. The additional studies like ADMET, QSAR, bioactivity scores, drug properties and toxicity risks ascertained them as newer drug candidates. This study had added a new collection of piperazino fused acridone derivatives to the existing array of other nitrogen heterocyclic fused acridone derivatives as anticancer agents.

## Introduction

Cancer is one of the leading diseases causing death worldwide and millions of people are getting affected every year and WHO estimates 12 million cancer deaths worldwide in 2030^1^. Despite the great strides made in the treatment of cancer over the past 50 years, it continues to be a major health concern and therefore, extensive efforts have been devoted to search for novel scaffolds to develop chemo-therapeutics for the treatment of cancer. Although many drugs are available for its treatment, their resistance and low-specificity are the key challenges available for the medicinal chemists^2^. Hence, the development of specific and potential anticancer drugs is a promising aspect of the moment. In such, many researchers have aimed to synthesize new anticancer drugs, amongst such compounds acridone derivatives plays a key role as acridine based natural and synthetic compounds are a *vital* class of nitrogen heterocycles. These compounds are attentive as they exhibit an extensive array of pharmaceutical properties as it is being an integral part of natural products and important heterocycles in medicinal chemistry.

These acridone compounds are prompt to exhibit bioactivities like anticancer^3^, antimalarial^4,5^, antitubercular^6^, antiviral^7,8^, anti-inflammatory^9^, antiparasitic^10^, antimicrobial^11^, fungicidal^12^, anti-psoriatic^13^, anti-candidiasis^14^ and anti-biofilm^15^ activities. The acridone based alkaloids acronycine (**I**)^16^ and glyfoline (**II**)^17^ were found as potent human leukemia cell (HL60) growth inhibitors. The triazolo acridone derivatives C-1533 (**IIIa**) and C-1305 (**IIIb**) were known as anticancer agents against leukaemia, melanomas and colon adenocarcinoma cell lines^18^. Similarly the imidazole fused acridone viz*.,* symadex (**IV**) has been identified as antitumor agent^19,20^. The pyrano fused acridone derivative viz*.,* elacridar (**V**) is reported as a potent breast cancer resistance proteins inhibitor^21,22^. Similarly, atalaphyllidine (**VI**) has been identified as human lung adenocarcinoma (A549) cell growth inhibitors^23^. On the other hand acrifoline (**VII**), chlorospermine A (**VIII**) and chlorospermine B (**IX**) were reported as potential inhibitor of DYRK1A, a therapeutic cancer target which regulates the cell cycle progression of tumors and oncogenes^24,25^.

**Figure 1:**
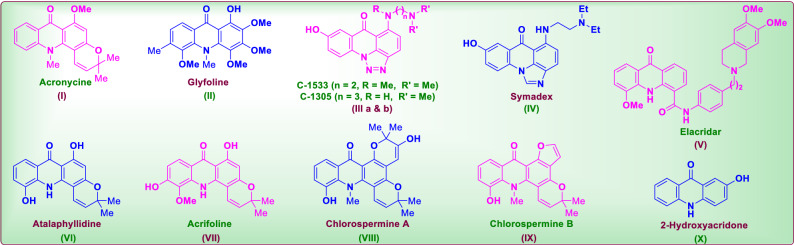
Natural products with acridone framework.

Similarly, 2-hydroxyacridone (**X**) and its substituted derivatives were reported as DNA topoisomerase II and protein kinase C inhibitors^26^ (Fig. 1). It is also reported that some of the synthesized fluorinated acridone derivatives as potent anticancer agents against MCF7, A549 and HT29 cell lines^27^. Similarly, the versatile biological activity of acridone and piperazine hybrids/conjugates^28^ like multifunctional cholinesterase inhibition acivity^29^, anticancer agents^30^, and antimicrobial activity^31^, hAChE and hBChE inhibitors^32^. This striking segment stimulated us to conjugate piperazine ring onto parent acridone ring to greatly induce its potentiality. This hypothesis has been realized by generating a piperazine fused acridone ring that is appended with an amide linker comprising of alicyclic/ aromatic/ hetero-aromatic rings by *N*-aroylation/ *N*-aroylation. In ultimate this study has realized the concept making the newer molecules with enhanced lipophilicity and binding interactions towards the interacting proteins. The merit of this accomplishment is that all existing nitrogen heterocycle fused acridone derivatives includes triazole, imidazole and pyrimidine rings in them, this is the first report on the synthesis and anticancer activity evaluation of the piperazine fused acridone derivatives. Moreover the *tertiary* amide linker (compared to the tertiary amines) impregnated on the piperazine ring has been proved as a prominent structural feature of the study.

## Materials and methods

### Chemistry

Reagent grade chemicals and analytical grade solvents were procured from Sigma-Aldrich and used for the synthesis, characterization and biological screening of title compounds. The analytical reagent [AR] grade solvents were purified by literature methods^33^. TLC monitoring was performed on alumina supported silica gel 60 F254 plates (Merck, Darmstadt, Germany) and visualized under UV light. All ^1^H (400 MHz) and ^13^C NMR (400 MHz) spectra were recorded on Bruker NMR spectrometer. Chemical shifts were reported in ppm (δ) with reference to TMS (internal standard) using CDCl_3_, DMSO-d_6_ and TFA as solvents. Coupling constant (J) values were given in Hertz and the multiplicities were designated as br, broad; s, singlet; d, doublet; t, triplet; m, multiplet. Molecular weights of the synthesized compounds were checked by SHIMADZU LCMS-2020 series in ESI mode. Melting points were recorded in capillaries and on Buchi Melting Point B-540 and are uncorrected. IR data was recorded on Perkin Elmer Spectrum 100. Column chromatography was performed with 100–200 mesh silica.

### Cytotoxicity assay

Anticancer activity of **7a–s** has been evaluated by MTT [3-(4,5-dimethylthiazol-2-yl)-2,5-diphenyl-tetrazolium bromide] assay and the cell lines HT29 (ATCC: HTB-38), MDA-MB-231 (ATCC: HTB-26), HEK293T (ATCC: CRL-3216) were procured from American type cell culture collection (ATCC), Manassas, USA and other chemicals were procured from Sigma-Aldrich. Then absorbance (used as a measure of cell proliferation) was measured at 570 nm with a kinetic micro-plate reader (BioTek, Winooski, Vermont).

## Experimental

### Chemistry

**Scheme 1:**
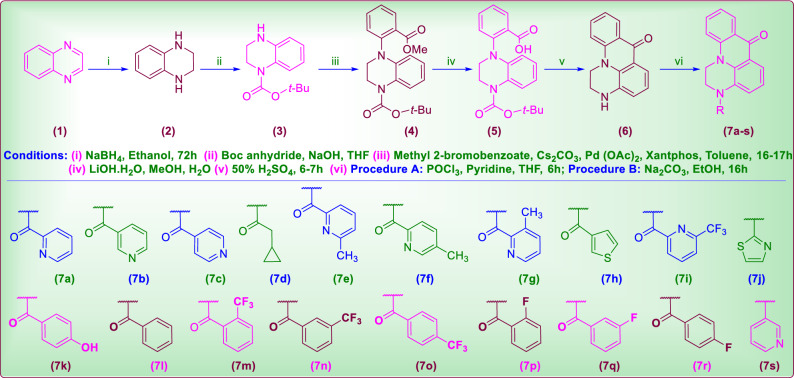
Synthesis of 3-aryl/aroyl-2,3-dihydro-1*H*,7H-pyrazino[3,2,1-*de*]acridin-7-one derivatives.

Novel 3-(alkyloyl/ aryloyl/ heteroaryloyl)-2,3-dihydropyrazino [3,2,1-de]acridin-7(1H)-ones and 3-(heteroaryl)-2,3-dihydro pyrazino [3,2,1-de]acridin-7(1H)-ones **(7a–s)** were designed by fusing of novel piperazine and acridone rings (Scheme 1). Synthetically, tetrahydroquinoxaline (**2**) is prepared from NaBH_4_ reduction of quinoxaline (**1**) in EtOH and protected with mono-Boc. Later the mono-Boc-protected tetrahydroquinoxaline (**3**) in toluene was reacted with methyl 2-bromobenzoate, in presence of Cs_2_CO_3_–Pd(OAc)_2_–Xantphos catalyst system at 100 °C to form o-methyl ester derivative of tetrahydroquinoxaline (**4**). Then **4** on mild hydrolysis with LiOH produced its acid derivative **5**, which up on 50% Sulfuric acid (50% H_2_SO_4_) treatment produced the tetracyclic compound **6**. Then on treating **6** with corresponding acids/ halides of alkyl/ aryl/ heteroaryl moieties produced **7a–s**. The structures of all the synthesized compounds have been confirmed by IR, ^1^H, ^13^C NMR, Mass spectral analytical and HPLC analytical studies and the spectra were provided in supplementary information.

### 1,2,3,4-Tetrahydroquinoxaline (2)

Sodium borohydride^34^ (NaBH_4_, 16.78 g, 441.83 mmol) was added to the mixture of quinoxaline (**1**, 23 g, 176.732 mmol) in ethanol (EtOH, 230 mL). Then it was purged with nitrogen, attached to mineral oil bubbler and stirred for 72 h at r.t. Then the same equivalent portion of NaBH_4_ in EtOH was added again and was purged for 48–72 h more. Then ethanol was evaporated under vacuum and the residue was dissolved in EtOAc (ethyl acetate) and washed with 3 M sodium hydroxide (NaOH), water and brine solution. Then organic phase was dried over anhydrous Na_2_SO_4_ (sodium sulphate), solvent was distilled off and the residue obtained was triturated with hexane to isolate pure compound **2** (Yield: 97%; mp: 98–101 °C). IR (KBr, cm^−1^) ν_max_: 3244 (NH); ^1^H NMR (400 MHz, CDCl_3_): δ 6.59–6.55 (m, 2H), 6.50–6.46 (m, 2H), 3.58–3.51 (br s, 2H), 3.40 (s, 4H); ^13^C NMR (400 MHz, CDCl_3_): δ 133.62, 118.68, 114.64, 41.32; MS (ESI) [M + H]^+^ Calcd for C_8_H_10_N_2_: 134.08; found: 135.07; HPLC: 97.34%.

### t-Butyl 3,4-dihydroquinoxaline-1(2H)-carboxylate (3)

Compound **2** obtained above was added with NaOH (8.23 g, 205.968 mmol) and boc anhydride (t-butoxycarbonyl anhydride, 19.69 mL, 85.82 mmol) in tetrahydrofuran (THF, 250 mL) and water (250 mL), the obtained reaction mixture was stirred for 16 h at r.t. Then the resulting contents were extracted with EtOAc and organic phase was dried over anhydrous Na_2_SO_4_. Then the solvent was distilled off to obtain solid compound **3** (Yield: 50%; mp: 90–92 °C). IR (KBr, cm^−1^) ν_max_: 1673 (C=O), 3396 (NH); ^1^H NMR (400 MHz, CDCl_3_): δ 7.49–7.47 (d, J = 6.4 Hz, 1H), 6.89–6.85 (t, J = 7.6 Hz, 1H) 6.65–6.61 (t, J = 7.6 Hz, 1H), 6.54–6.52 (d, J = 8.0 Hz, 1H), 3.97 (br s 1H), 3.75–3.73 (t, J = 4.8 Hz, 2H), 3.38–3.36 (t, J = 4.8 Hz, 2H), 1.51 (s, 9H); ^13^C NMR (400 MHz, CDCl_3_): δ 153.23, 136.72, 124.60, 124.52, 116.52, 114.44, 80.81, 41.97, 28.31; MS (ESI) [M + H]^+^ Calcd for C_13_H_18_N_2_O_2_: 234.14; found: 235.34; HPLC: 98.37%.

### t-Butyl 4-(2-(methoxycarbonyl) phenyl)-3,4-dihydroquinoxaline-1(2H)-carboxylate (4)

To the mixture of compound **3** (3.0 g, 12.83 mmol) and methyl 2-bromobenzoate (5.51 g, 25.64 mmol), a catalytic system of cesium carbonate (Cs_2_CO_3_, 10.44 g, 32.05 mmol), palladium(II) acetate (Pd(OAc)_2_, 0.01 mmol) and xantphos (9,9-Dimethyl-4,5-bis(diphenyl phosphino) xanthene, 0.01 mmol) were added in toluene (30 mL) and then degasified by purging nitrogen for 15 min and stirred for 16–17 h at 100 °C. Then the reaction mixture was extracted with EtOAc. The organic phase was dried over anhydrous Na_2_SO_4_ and then solvent was distilled off and the residue was column chromatographed using EtOAc and hexane (5:1) as eluents and isolated compound **4** (Yield: 72%; mp: 73–74 °C); IR (KBr, cm^−1^) ν_max_: 1693 (O=C–N_piper_), 1726 (O=C–C_aryl_), 2938 (C=C–N_piper_); ^1^H NMR (400 MHz, CDCl_3_): δ 7.89–7.86 (dd, 1H, ^2^J = 8.0 Hz, ^3^J = 1.6 Hz, ArH), 7.59–7.54 (dt, ^2^J = 7.8 Hz, ^3^J = 1.6 Hz, 1H) 7.50–7.48 (br d, J = 7.6 Hz, 1H), 7.34–7.31 (t, J = 7.6 Hz, 2H), 6.78–6.74 (dt, ^2^J = 8.0 Hz, ^3^J = 1.2 Hz, 1H) 6.67–6.63 (dt, ^2^J = 8.2 Hz, ^3^J = 1.2 Hz, 1H) 6.26–6.24 (dd, ^2^J = 8.2 Hz, ^3^J = 1.0 Hz, 1H), 3.94–3.92 (t, J = 4.6 Hz, 2H), 3.70–3.68 (t, J = 4.8 Hz, 2H), 3.62 (s, 3H), 1.55 (s, 9H); ^13^C NMR (400 MHz, CDCl_3_): δ 167.08, 153.22, 146.13, 137.82, 133.65, 131.88, 130.04, 129.72, 126.31, 125.35, 124.73, 124.51, 116.97, 113.71, 80.95, 52.13, 51.328, 41.38, 28.41; MS (ESI) [M + H]^+^ calcd for C_21_H_24_N_2_O_4_: 368.43; found, 369.46; HPLC: 98.95%.

### 2-(4-(t-Butoxycarbonyl)-3,4-dihydroquinoxalin-1(2H)-yl)benzoic acid (5)

A mixture of compound **4** (20 g, 54.34 mmol), lithium hydroxide (LiOH) monohydrate (11.40 g, 543.47 mmol) in methanol (100 mL) and water (50 mL) was stirred for 16–17 h at r.t. The solvent distilled off and the residue was extracted with EtOAc. The organic phase was dried over anhydrous Na_2_SO_4_ and solvent distilled out to isolate pure compound **5** (Yield: 83.2%; mp: 169–171 °C); IR (KBr, cm^−1^) ν_max_: 1593 (C=O), 1699 (C=O), 3443 (OH): ^1^H NMR (400 MHz, DMSO-d_6_): δ 12.83 (s, 1H, COOH), 7.85–7.82 (dd, ^2^J = 7.8 Hz, ^3^J = 1.2 Hz, 1H), 7.67–7.63 (dt, ^2^J = 7.6 Hz, ^3^J = 1.2 Hz, 1H), 7.44–7.37 (m, 3H, ArH), 6.73–6.69 (dt, ^2^J = 8.4 Hz, ^3^J = 1.4 Hz, 1H), 6.57–6.53 (dt, ^2^J = 8.2 Hz, ^3^J = 1.2 Hz, 1H), 6.06–6.03 (dd, ^2^J = 8.2 Hz, ^3^J = 1.0 Hz, 1H), 3.81–3.79 (t, J = 4.8 Hz, 2H), 3.61–3.59 (t, J = 4.6 Hz, 2H), 1.46 (s, 9H); ^13^C NMR (400 MHz, DMSO-d_6_): δ 167.40, 152.36, 144.82, 137.54, 133.53, 131.19, 131.13, 130.18, 126.79, 124.40, 124.16, 115.83, 112.46, 80.25, 50.50, 40.72, 40.12, 27.96; MS (ESI) [M + H]^+^ calcd for C_20_H_22_N_2_O_4_: 354.40; found: 355.15; HPLC: 98.79%.

### 3-Dihydropyrazino[3,2,1-de]acridin-7(1H)-one (6)

50% H_2_SO_4_^14^ in water (15 mL, 15 v/w) and compound **5** (1 g, 2.824 mmol) stirred for 6–7 h at r.t. Reaction mixture was poured into ice cold water and extracted with EtOAc. The organic phase was dried over anhydrous Na_2_SO_4_. The solvent distilled off and the residue was column chromatographed using EtOAc and hexane (7:1) as eluents and isolated pure compound **6** (Yield: 63%; mp: 225–227 °C); IR (KBr, cm^−1^) ν_max_: 1602 (C=O), 3270 (NH); ^1^H NMR (400 MHz, DMSO-d_6_): δ 8.33 (d, 1H, J = 8.0 Hz), 7.79–7.74 (m, 2H), 7.60–7.58 (m, 1H), 7.30 (t, 1H, J = 7.2 Hz), 7.07–6.99 (m, 2H), 6.42 (s, 1H, NH), 4.34 (t, 2H, J = 5.0 Hz), 3.52 (t, 2H, J = 4.4 Hz); ^13^C NMR (400 MHz, DMSO-d_6_): δ 176.33, 140.75, 137.40, 133.51, 129.36, 126.51, 121.72, 121.42, 120.93, 120.85, 115.59, 114.78, 114.13, 44.92, 40.12. MS (ESI) [M + H]^+^: calcd for C_15_H_12_N_2_O: 236.27; found: 237.12; HPLC: 97.54%.

### 3-(Alkyloyl/ aryloyl/ heteroaryloyl)-2,3-dihydropyrazino [3,2,1-de]acridin-7(1H)-ones^35^

**Procedure A.** Mixture of **6** (1.0 mmol) and suitable carboxylic acid (1.0 mmol) was treated with POCl_3_ (1.5 mmol) and pyridine (3.0 mmol) in Tetrahedrofuran (10 volumes) at r.t. for 16 h. On completion of reaction, it was extracted into EtOAc and organic phase was dried over anhydrous Na_2_SO_4_. Then the solvent was distilled off and the residue was column chromatographed using EtOAc and hexane (10:1) to isolate **7a–i **and** 7k–r **in pure form.

### 3-(Heteroaryl)-2,3-dihydropyrazino [3,2,1-de]acridin-7(1H)-ones

**Procedure B.** Mixture of **6** (1.0 mmol) and appropriate bromide (3.0 mmol) was treated with Na_2_CO_3_ (6.0 mmol) in ethanol (10volumes) at r.t. for 16 h. On completion of reaction, solvent was distilled out and extracted with EtOAc. The organic phase was dried over anhydrous Na_2_SO_4_. Then the solvent was distilled off and the residue was column chromatographed using EtOAc and hexane (10:1) as eluents to isolate **7j** and **7s** in pure form.

### 3-Picolinoyl-2,3-dihydropyrazino[3,2,1-de]acridin-7(1H)-one (7a)

Yellow solid, Yield: 156.0 mg (71.9%); mp: 232–234 °C; IR (KBr, cm^−1^) ν_max_: 1633 (C=O), 1650 (C=O); ^1^H NMR (400 MHz, DMSO-d_6_): δ 8.49 (br s, 1H), 8.37–8.35 (d, J = 8.0 Hz, 1H), 8.08 (br s, 1H), 7.95 (br s, 1H), 7.91–7.87 (t, J = 7.8 Hz, 1H), 7.77–7.75 (d, J = 8.0 Hz, 2H), 7.50 (br s, 1H), 7.42–7.38 (t, J = 7.4 Hz, 1H), 7.05 (br s, 2H), 4.46 (s, 2H), 4.28 (s, 2H); ^13^C NMR (400 MHz, DMSO-d_6_): δ 176.13, 166.48, 153.07, 148.46, 141.05, 137.46, 134.27, 132.62, 127.85, 126.46, 125.22, 124.09, 123.01, 121.85, 121.78, 121.17, 119.99, 115.09, 46.25; MS (ESI) [M + H]^+^ Calcd for C_21_H_15_N_3_O_2_: 341.36; found: 342.1; HPLC: 98.01%.

### 3-Nicotinoyl-2,3-dihydropyrazino[3,2,1-de]acridin-7(1H)-one (7b)

Yellow solid; Yield: 142.0 mg (65.5%); mp: 231–233 °C; IR (KBr, cm^−1^) ν_max_: 1607 (C=O), 1633 (C=O); ^1^H NMR (400 MHz, DMSO-d_6_): δ 8.65 (s, 1H), 8.62–8.61 (d, J = 3.6 Hz, 1H), 8.38–8.36 (dd, ^2^J = 7.8 Hz, ^3^J = 1.4 Hz, 1H), 8.09–8.07 (d, J = 8.0 Hz, 1H), 7.92–7.87 (m, 2H, ArH), 7.78–7.76 (d, J = 8.8 Hz, 1H), 7.43–7.39 (t, J = 7.2 Hz, 2H), 7.26 (br s, 1H), 7.04 (br s, 1H), 4.52–4.50 (t, J = 5.0 Hz, 2H), 4.27 (s, 2H); ^13^C NMR (400 MHz, DMSO-d_6_): δ 176.14, 163.00, 150.96, 149.08, 141.13, 136.20, 134.20, 132.60, 130.93, 128.41, 127.48, 126.43, 123.17, 121.80, 121.21, 119.81, 115.13, 46.11; MS (ESI) [M + H]^+^ Calcd for C_21_H_15_N_3_O_2_: 341.36; found: 342.12; HPLC: 98.78%.

### 3-Isonicotinoyl-2,3 dihydropyrazino[3,2,1-de]acridin-7(1H)-one (7c)

Yellow solid, Yield: 148.0 mg (68.5%); mp: 233–235 °C; IR (KBr, cm^−1^) ν_max_: 1635 (C=O), 1648 (C=O); ^1^H NMR (400 MHz, DMSO-d_6_): δ 8.63 (s, 2H), 8.38–8.35 (dd, ^2^J = 8.2 Hz, ^3^J = 1.4 Hz, 1H), 8.11–8.09 (d, J = 8.0 Hz, 1H), 7.91–7.87 (m, 1H, ArH), 7.77–7.75 (d, J = 8.8 Hz, 1H), 7.47 (s, 1H), 7.42–7.39 (t, J = 7.4 Hz, 2H), 7.06 (br s, 2H), 4.49 (s, 2H), 4.23 (s, 2H); ^13^C NMR (400 MHz, DMSO-d_6_): δ 176.13, 166.18, 149.95, 142.65, 141.12, 134.25, 132.67, 128.32, 126.95, 126.46, 123.44, 122.21, 121.85, 121.79, 121.23, 119.81, 115.13, 46.01, 40.12; MS (ESI) [M + H]^+^ Calcd for C_21_H_15_N_3_O_2_: 341.36; found: 342.06: HPLC: 98.02%.

### 3-(2-Cyclopropylacetyl)-2,3-dihydropyrazino[3,2,1-de]acridin-7(1H)-one (7d)

Pale yellow solid; Yield: 42.0 mg (31.1%); mp: 238–240 °C; IR (KBr, cm^−1^) ν_max_: 1594 (C=O), 1625 (C=O); ^1^H NMR (400 MHz, DMSO-d_6_): δ 8.36–8.33 (dd, ^2^J = 7.8 Hz, ^3^J = 1.4 Hz, 1H), 8.16–8.14 (dd, ^2^J = 8.0 Hz, ^3^J = 1.2 Hz, 1H), 7.89–7.85 (m, 2H, ArH), 7.75–7.73 (d, J = 8.8 Hz, 1H), 7.41–7.37 (t, J = 7.4 Hz, 1H), 7.31–7.27 (t, J = 7.8 Hz, 1H), 4.34 (s, 2H), 4.16 (s, 2H), 2.56–2.54 (d, J = 6.4 Hz, 2H), 0.98 (s, 1H), 0.43 (s, 2H), 0.09 (s, 2H); ^13^C NMR (400 MHz, DMSO-d_6_): δ 176.22, 170.87, 141.11, 134.19, 133.02, 128.33, 126.41, 121.79, 121.17, 120.18, 115.13, 46.36; MS (ESI) [M + H]^+^ Calcd for C_20_H_18_N_2_O_2_: 318.37; found: 319.21; HPLC: 98.46%.

### 3-(6-Methylpicolinoyl)-2,3-dihydropyrazino[3,2,1-de]acridin-7(1H)-one (7e)

Pale yellow solid; Yield: 64.0 mg (42.5%); mp: 246–248 °C; IR (KBr, cm^−1^) ν_max_: 1597 (C=O), 1634 (C=O); ^1^H NMR (400 MHz, DMSO-d_6_): δ 8.33–8.32 (d, J = 6.8 Hz, 1H), 8.06 (s, 1H), 7.86–7.70 (m, 3H, ArH), 7.48 (s, 1H), 7.38–7.34 (t, J = 7.6 Hz, 2H), 7.11 (br s, 2H), 4.41 (s, 2H), 4.20 (s, 2H), 2.37 (s, 3H); ^13^C NMR (400 MHz, DMSO-d_6_): δ 176.14, 166.55, 150.31, 148.58, 141.04, 137.59, 135.00, 134.25, 132.56, 127.79, 126.46, 123.80, 122.88, 121.82, 121.78, 121.15, 120.02, 115.08, 46.28, 17.90; MS (ESI) [M + H]^+^ Calcd for C_22_H_17_N_3_O_2_: 355.39; found: 356.15: HPLC: 91.00%.

### 3-(5-Methylpicolinoyl)-2,3-dihydropyrazino[3,2,1-de]acridin-7(1H)-one (7f)

Yellow solid; Yield: 90.0 mg (59.8%); mp: 252–254 °C; IR (KBr, cm^−1^) ν_max_: 1598 (C=O), 1636 (C=O); ^1^H NMR (400 MHz, DMSO-d_6_): δ 8.37–8.35 (d, J = 7.2 Hz, 2H), 8.09–8.07 (d, J = 6.8 Hz, 1H), 7.90–7.87 (t, J = 7.4 Hz, 1H), 7.66–7.74 (d, J = 8.4 Hz, 2H), 7.68–7.66 (d, J = 8.0 Hz, 1H), 7.42–7.38 (t, J = 7.4 Hz, 1H), 7.09 (br s, 2H), 4.45 (s, 2H), 4.28 (s, 2H), 2.34 (s, 3H); ^13^C NMR (400 MHz, DMSO-d_6_): δ 176.14, 166.55, 150.31, 148.58, 141.04, 137.59, 135.00, 134.25, 132.56, 127.79, 126.46, 123.80, 122.88, 121.82, 121.78, 121.15, 120.02, 115.08, 46.28, 17.90; MS (ESI) [M + H]^+^ Calcd for C_22_H_17_N_3_O_2_: 355.39; found: 355.81; HPLC: 97.42%.

### 3-(3-Methylpicolinoyl)-2,3-dihydropyrazino[3,2,1-de]acridin-7(1H)-one (7g)

Yellow solid; Yield: 52.0 mg (34.5%); mp: 221–223 °C; IR (KBr, cm^−1^) ν_max_: 1599 (C=O), 1661 (C=O); ^1^H NMR (400 MHz, TFA): δ 8.77–8.75 (d, J = 8.4 Hz, 1H), 8.66–8.64 (d, J = 7.6 Hz, 2H), 8.49 (s, 1H), 8.29–8.25 (t, J = 7.8 Hz, 1H), 8.13 (s, 1H), 8.02 (s, 1H), 7.86–7.82 (t, J = 7.8 Hz, 1H), 7.51 (br s, 2H), 5.12 (s, 2H), 4.45 (s, 2H), 2.52 (s, 3H); ^13^C NMR (400 MHz, DMSO-d_6_): δ 176.14, 166.55, 150.31, 148.58, 141.04, 137.59, 135.0, 134.25, 132.56, 127.79, 126.46, 123.80, 122.88, 121.82, 121.78, 120.02, 115.08, 46.28, 17.90; MS (ESI) [M + H]^+^ Calcd for C_22_H_17_N_3_O_2_: 355.39; found: 356.20; HPLC: 99.62%.

### 3-(Thiophene-3-carbonyl)-2,3-dihydropyrazino[3,2,1-de]acridin-7(1H)-one (7h)

Yellow solid; Yield: 66.0 mg (45.0%); mp: 254–256 °C; IR (KBr, cm^−1^) ν_max_: 1608 (C=O), 1637 (C=O); ^1^H NMR (400 MHz, DMSO-d_6_): δ 8.38–8.35 (dd, ^2^J = 8.0 Hz, ^3^J = 1.6 Hz, 1H), 8.11–8.08 (dd, ^2^J = 8.2 Hz, ^3^J = 1.4 Hz, 1H), 7.91–7.86 (m, 2H, ArH), 7.76–7.74 (t, J = 8.4 Hz, 1H), 7.55–7.53 (m, 1H, ArH), 7.42–734 (m, 2H, ArH), 7.12–7.08 (m, 2H, ArH), 4.45 (s, 2H), 4.26 (s, 2H). ^13^C NMR (400 MHz, DMSO-d_6_): δ 176.19, 163.51, 141.08, 135.84, 134.16, 132.48, 130.03, 127.89, 127.83, 127.73, 126.49, 126.43, 122.87, 121.83, 121.71, 121.17, 119.88, 115.08, 46.22; MS (ESI) [M + H]^+^ Calcd for C_20_H_14_N_2_O_2_S: 346.08; found: 347.21; HPLC: 98.18%.

### 3-(6-(Trifluoromethyl)picolinoyl)-2,3-dihydropyrazino[3,2,1-de]acridin-7(1H)-one (7i)

Yellow solid; Yield: 62.0 mg (35.7%); mp: 234–236 °C; IR (KBr, cm^−1^) ν_max_: 1606 (C=O), 1636 (C=O); ^1^H NMR (400 MHz, DMSO-d_6_): δ 8.87 (s, 1H), 8.39–8.36 (dd, ^2^J = 8.0 Hz, ^3^J = 1.2 Hz, 1H), 8.18 (s, 1H), 8.12–8.10 (d, J = 8.0 Hz, 1H), 7.95–7.88 (m, 2H, ArH), 7.79–7.77 (d, J = 8.8 Hz, 1H), 7.43–7.40 (t, J = 7.6 Hz, 1H), 7.06 (br s, 2H), 4.52 (s, 2H), 4.29 (s, 2H); ^13^C NMR (400 MHz, DMSO-d_6_): δ 176.12, 141.13, 134.26, 132.75, 128.54, 126.46, 123.56, 121.85, 121.25, 120.38, 119.82, 115.11, 46.00; MS (ESI) [M + H]^+^ Calcd for C_22_H_14_F_3_N_3_O_2_: 409.36; found: 410.14: HPLC: 99.93%.

### 3-(Thiazol-2-yl)-2,3-dihydropyrazino[3,2,1-de]acridin-7(1H)-one (7j)

Pale yellow solid; Yield: 92.0 mg (68%); mp: 239–241 °C; IR (KBr, cm^−1^) ν_max_: 1593 (C=O), 1626 (C=O); ^1^H NMR (400 MHz, DMSO-d_6_): δ 8.37–8.35 (dd, ^2^J = 8.0 Hz, ^3^J = 1.6 Hz, 1H), 8.19–8.17 (dd, ^2^J = 7.8 Hz, ^3^J = 1.4 Hz, 1H), 8.14–8.12 (dd, ^2^J = 8.0 Hz, ^3^J = 1.6 Hz, 1H), 7.89–7.70 (dt, ^2^J = 8.0 Hz, ^3^J = 1.6 Hz, 1H), 7.79–7.77 (d, J = 8.4 Hz, 1H), 7.41–7.31 (m, 3H, ArH), 7.06–7.05 (d, J = 3.6 Hz, 1H), 4.42–4.40 (d, J = 5.6 Hz, 2H), 4.36–4.35 (d, J = 5.6 Hz, 2H); ^13^C NMR (400 MHz, DMSO-d_6_): δ 176.17, 167.72, 140.95, 139.28, 134.11, 132.04, 130.74, 126.42, 124.38, 122.23, 122.08, 121.77, 121.17, 120.63, 115.09, 110.31, 44.33, 44.16; MS (ESI) [M + H]^+^ Calcd for C_18_H_13_N_3_OS: 319.38; found: 320.19: HPLC: 98.29%.

### 3-(4-hydroxybenzoyl)-2,3-dihydro-1H,7H-pyrazino[3,2,1-de]acridin-7-one (7k)

Yellow solid; Yield: 40.0 mg (26.6%); mp: 265–267 °C; IR (KBr, cm^−1^) ν_max_: 1570 (C=O), 1654 (C=O); ^1^H NMR (400 MHz, DMSO-d_6_): δ 10.70–9.70 (bs, 1H), 8.38–8.33 (m, 1H), 8.06–8.04 (m, 1H), 7.90–7.87 (m, 1H), 7.77–7.75 (m, 1H), 7.41–7.31 (m, 3H), 7.26–7.19 (m, 1H), 7.08–7.04 (m, 1H), 6.74–6.72 (m, 2H), 4.47–4.44 (m, 2H), 4.23 (bs, 2H); ^13^C NMR (400 MHz, DMSO-d_6_): δ 176.26, 168.28, 159.95, 141.11, 134.23, 132.25, 130.92, 128.72, 128.00, 126.48, 124.77, 122.36, 121.85, 121.76, 121.17, 120.00, 115.19, 114.97, 79.25, 46.36; MS (ESI) [M + H]^−^ Calcd for C_22_H_16_N_2_O_3_: 356.38; found: 356.96; HPLC: 97.58%.

### 3-Benzoyl-2,3-dihydro-1H,7H-pyrazino[3,2,1-de]acridin-7-one (7l)

Yellow solid; Yield: 35.0 mg (48.6%); mp: 253–255 °C; IR (KBr, cm^−1^) ν_max_: 1628 (C=O), 1655 (C=O); ^1^H NMR (400 MHz, CDCl_3_): δ 8.61–8.56 (m, 1H), 8.31–8.25 (m, 1H), 7.83–7.76 (m, 1H), 7.55–7.47 (m, 1H), 7.46–7.28 (m, 6H), 7.09–6.99 (s, 1H), 6.98–6.91 (m, 1H), 4.42 (s, 4H); ^13^C NMR (400 MHz, CDCl_3_): δ 177.82, 169.01, 141.59, 134.49, 132.73, 131.25, 129.05, 128.71, 128.20, 127.97, 124.64, 123.06, 122.44, 120.52, 113.86, 47.01, 40.45; MS (ESI) [M + H]^−^ Calcd for C_22_H_16_N_2_O_2_: 340.12; found: 340.81; HPLC: 99.51%

### 3-(2-(Trifluoromethyl)benzoyl)-2,3-dihydro-1H,7H-pyrazino[3,2,1-de]acridin-7-one (7m)

Yellow solid; Yield: 45.0 mg (52%); mp: 252–254 °C; IR (KBr, cm^−1^) ν_max_: 1637 (C=O), 1505 (C=O); ^1^H NMR (400 MHz, CDCl_3_): δ 8.63–8.53 (m, 1H), 8.47–8.33 (m, 1H), 8.30–8.18 (m, 1H), 7.89–7.59 (m, 3H), 7.60–7.44 (m, 2H), 7.43–7.30 (m, 2H), 6.88–6.72 (m, 1H), 4.55–4.40 (m, 2H), 4.29–4.19 (m, 1H), 3.94–3.84 (m, 1H); ^13^C NMR (400 MHz, CDCl_3_): δ 177.42, 166.89, 157.84, 141.33, 134.26, 132.66, 131.71, 129.98, 128.46, 127.88, 126.97, 125.04, 122.69, 122.22, 120.79, 119.94, 113.66, 113.15, 45.91, 38.81; MS (ESI) [M + H]^−^ Calcd for C_23_H_15_F_3_N_2_O_2_: 408.11; found: 408.94; HPLC: 99.33%.

### 3-(3-(Trifluoromethyl)benzoyl)-2,3-dihydro-1H,7H-pyrazino[3,2,1-de]acridin-7-one (7n)

Yellow solid; Yield: 50.0 mg (58%); mp: 247–249 °C; IR (KBr, cm^−1^) ν_max_: 1637 (C=O), 1505 (C=O); ^1^H NMR (400 MHz, CDCl_3_): δ 8.62–8.58 (m, 1H), 8.34–8.29 (m, 1H), 7.84–7.77 (m, 2H), 7.69 (d, J = 7.7 Hz, 1H), 7.54–7.47 (m, 2H), 7.44–7.37 (m, 2H), 7.00–6.92 (m, 2H), 4.45 (s, 4H); ^13^C NMR (400 MHz, CDCl_3_): δ 177.08, 166.60, 140.98, 134.73, 133.97, 132.15, 131.59, 131.11, 130.70, 128.50, 127.98, 127.64, 127.28, 126.72, 125.66, 124.60, 122.55, 121.94, 119.85, 113.24, 46.30, 39.84; MS (ESI) [M + H]^−^ Calcd for C_23_H_15_F_3_N_2_O_2_: 408.11; found: 408.9; HPLC: 98.21%.

### 3-(4-(Trifluoromethyl)benzoyl)-2,3-dihydro-1H,7H-pyrazino[3,2,1-de]acridin-7-one (7o)

Pale yellow solid; Yield: 25.0 mg (29%); mp: 240–242 °C; IR (KBr, cm^−1^) ν_max_: 1637 (C=O), 1602 (C=O); ^1^H NMR (400 MHz, DMSO-d_6_): δ 8.37 (d, 1H, J = 7.6 Hz), 8.09 (d, 1H, J = 8 Hz), 7.90 (t, 1H, J = 7.4 Hz), 7.78–7.73 (m, 6H), 7.41 (t, 1H, J = 7.2 Hz), 7.06 (br s, 1H), 4.51 (br s, 2H), 4.26 (br s, 2H); ^13^C NMR (400 MHz, DMSO-d_6_): δ 176.18, 141.15, 134.32, 128.41, 126.50, 125.34, 123.28, 121.89, 121.83, 121.24, 119.91, 115.19; MS (ESI) [M + H]^−^ Calcd for C_23_H_15_F_3_N_2_O_2_: 408.11; found: 408.94; HPLC: 97.15%.

### 3-(2-Fluorobenzoyl)-2,3-dihydro-1H,7H-pyrazino[3,2,1-de]acridin-7-one (7p)

Yellow solid; Yield: 45.0 mg (60%); mp: 225–227 °C; IR (KBr, cm^−1^) ν_max_: 1650 (C=O), 1594 (C=O); ^1^H NMR (400 MHz, CDCl_3_): δ 8.60–8.52 (m, 1H), 8.31–8.26 (m, 1H), 7.82–7.77 (m, 1H), 7.49 (m, 1H), 7.37 (m, 1H), 7.30–7.29 (m, 1H), 7.24–7.23 (m, 1H), 7.22–7.10 (m, 3H), 6.98–6.94 (m, 1H), 4.46–4.37 (m, 4H); ^13^C NMR (400 MHz, CDCl_3_): δ 172.25, 171.42, 161.13, 155.35, 154.53, 148.63, 138.74, 138.17, 135.13, 128.22, 128.02, 127.86, 115.66, 117.20, 113.59, 80.01, 50.92, 50.60; MS (ESI) [M + H]^−^ Calcd for C_22_H_15_FN_2_O_2_: 358.11; found: 359.03; HPLC: 98.59%.

### 3-(3-Fluorobenzoyl)-2,3-dihydro-1H,7H-pyrazino[3,2,1-de]acridin-7-one (7q)

Yellow solid; Yield: 30.0 mg (40%); mp: 242–244 °C; IR (KBr, cm^−1^) ν_max_: 1650 (C=O), 1603 (C=O); ^1^H NMR (400 MHz, CDCl_3_): δ 8.61–8.56 (m, 1H), 8.32–8.27 (m, 1H), 7.83–7.76 (m, 1H), 7.49 (d, J = 8.68 Hz, 1H), 7.37 (t, J = 7.45 Hz, 1H), 7.31–7.28 (m, 1H), 7.25–7.23 (m, 1H), 7.22–7.09 (m, 3H), 6.99–6.94 (m, 1H), 4.46–4.38 (m, 4H); ^13^C NMR (400 MHz, CDCl_3_): δ 177.00, 166.84, 163.26, 160.79, 140.86, 136.09, 133.83, 132.04, 129.79, 129.71, 127.85, 127.44, 124.24, 123.99, 121.75, 119.83, 117.73, 117.52, 115.66, 115.43, 113.20, 46.24; MS (ESI) [M + H]^−^ Calcd for C_22_H_15_FN_2_O_2_: 358.11; found: 359.03; HPLC: 99.50%.

### 3-(4-Fluorobenzoyl)-2,3-dihydro-1H,7H-pyrazino[3,2,1-de]acridin-7-one (7r)

Yellow solid; Yield: 25.0 mg (33%); mp: 236–238 °C; IR (KBr, cm^−1^) ν_max_: 1630 (C=O), 1600 (C=O); ^1^H NMR (400 MHz, CDCl_3_): δ 8.62 -8.58 (m, 1H), 8.35–8.25 (m, 1H), 7.85–7.68 (m, 1H), 7.55–7.45 (m, 1H), 7.42–7.38 (m, 3H), 7.10–6.87 (m, 4H), 4.45–4.37 (m, 4H); ^13^C NMR (400 MHz, CDCl_3_): δ 177.00, 166.84, 163.26, 160.79, 140.86, 136.09, 133.83, 132.04, 129.39, 127.85, 127.44, 124.24, 121.75, 119.83, 117.52, 115.43, 113.20, 46.24, 39.89, 26.60; MS (ESI) [M + H]^−^ Calcd for C_22_H_15_FN_2_O_2_: 358.11; found: 359.03; HPLC: 98.60%.

### 3-(Pyridin-3-yl)-2,3-dihydropyrazino[3,2,1-de]acridin-7(1H)-one (7s)

Yellow solid; Yield: 41.0 mg (31.6%); mp: 244–246 °C; IR (KBr, cm^−1^) ν_max_: 1549 (C=O), 1621 (C=O); ^1^H NMR (400 MHz, DMSO-d_6_): δ 8.65 (s, 1H), 8.37–8.36 (d, J = 7.6 Hz, 2H), 7.92–7.78 (m, 4H, ArH), 7.58 (s, 1H), 7.39–7.36 (t, J = 7.0 Hz, 1H), 7.26–7.24 (d, J = 7.6 Hz, 1H), 7.12 (t, J = 7.4 Hz, 1H), 4.47 (s, 2H), 4.08 (s, 2H); ^13^C NMR (400 MHz, DMSO-d_6_): δ 176.27, 143.04, 141.38, 140.75, 133.95, 133.35, 131.48, 131.25, 126.43, 125.14, 122.23, 121.48, 121.00, 120.91, 119.04, 118.98, 115.07, 44.81. MS (ESI) [M + H]^+^ Calcd for C_20_H_15_N_3_O: 313.35; found: 314.15; HPLC: 89.86%.

### Cytotoxicity assay

Having synthesized a series of the title compounds, all of them were screened for their in vitro anticancer profiles against a panel of human colorectal adenocarcinoma (HT29), human breast adenocarcinoma (MDA-MB-231) and mutated Human embryonic kidney 293 (HEK293T) cancer cell lines by using MTT colourmetric assay as per Johan van Meerloo protocol^36^. The three cell lines were cultured in RPMI-1370 medium and supplemented with 10% FBS followed by 100 units/mL penicillin/ streptomycin and were cultured at 37 °C in 5% CO_2_. Cell proliferation of the growing cells was measured in terms of logarithmic standards. Analytically, all the cultured cells were plated onto 96 well plates at a starting density of 10^5^ cells. Then all the cells were treated with title compounds **7a–s** for a period of 48 h with different concentrations. After this incubation cell proliferation inhibition was determined by MTT assay and all the experiments were conducted in triplicates and readings were presented as mean ± SD and provided in Tables 1, 2 and 3.

**Table 1:** MTT assay of 7a–s against HT29.

Compound	Percentage of inhibition against treated concentrations (µM)					IC_50_ (µg/mL)
	1	5	10	25	50	
**7a**	78.91 ± 0.42	57.61 ± 0.39	44.06 ± 0.86	14.88 ± 0.36	8.77 ± 0.72	11.24 ± 0.68
**7b**	89.93 ± 0.66	65.63 ± 0.27	52.51 ± 0.82	38.08 ± 0.42	19.44 ± 0.35	20.72 ± 0.49
**7c**	90.02 ± 0.31	66.19 ± 0.83	52.93 ± 0.84	40.37 ± 0.15	20.79 ± 0.08	21.57 ± 0.31
**7d**	88.71 ± 0.78	63.12 ± 0.71	50.91 ± 0.38	29.68 ± 0.23	17.97 ± 0.39	18.26 ± 0.29
**7e**	84.92 ± 0.94	60.82 ± 0.33	49.12 ± 0.85	27.59 ± 0.35	16.26 ± 0.55	16.36 ± 0.48
**7f**	86.92 ± 0.24	61.38 ± 0.68	49.24 ± 0.05	29.09 ± 0.18	17.14 ± 0.65	17.19 ± 0.46
**7g**	79.09 ± 0.34	58.87 ± 0.93	45.11 ± 0.54	25.48 ± 0.44	10.01 ± 0.91	13.22 ± 0.57
**7h**	87.92 ± 0.24	62.38 ± 0.68	50.24 ± 0.65	29.49 ± 0.51	17.84 ± 0.65	17.85 ± 0.66
**7i**	83.56 ± 0.57	59.11 ± 0.75	46.51 ± 0.52	25.83 ± 0.28	12.22 ± 0.27	14.63 ± 0.14
**7j**	89.55 ± 0.11	64.84 ± 0.45	51.48 ± 0.94	31.05 ± 0.19	17.37 ± 0.87	18.86 ± 0.38
**7k**	96.62 ± 0.99	81.84 ± 0.11	74.68 ± 0.64	66.23 ± 0.18	28.03 ± 0.96	33.63 ± 0.21
**7l**	97.17 ± 0.22	83.16 ± 0.99	76.71 ± 0.06	68.88 ± 0.47	30.59 ± 0.87	35.55 ± 0.29
**7m**	92.71 ± 0.01	76.71 ± 0.17	68.51 ± 0.72	57.33 ± 0.32	23.79 ± 0.33	29.09 ± 0.93
**7n**	95.32 ± 0.73	79.12 ± 0.33	72.72 ± 0.33	59.26 ± 0.11	26.92 ± 0.84	31.35 ± 0.24
**7o**	93.92 ± 0.39	77.83 ± 0.91	69.54 ± 0.24	58.23 ± 0.26	24.32 ± 0.76	29.72 ± 0.32
**7p**	90.43 ± 0.77	74.71 ± 0.22	65.84 ± 0.76	54.13 ± 0.37	20.98 ± 0.22	26.94 ± 0.97
**7q**	94.96 ± 0.53	78.87 ± 0.43	70.57 ± 0.53	58.23 ± 0.29	25.33 ± 0.44	30.32 ± 0.86
**7r**	91.94 ± 0.93	75.12 ± 0.73	66.15 ± 0.13	55.26 ± 0.23	21.57 ± 0.24	27.52 ± 0.50
**7s**	84.78 ± 0.12	60.38 ± 0.24	48.41 ± 0.57	27.49 ± 0.35	15.21 ± 0.61	15.98 ± 0.44
**Doxorubicin**	97.09 ± 0.79	90.76 ± 0.83	82.92 ± 0.54	72.56 ± 0.83	42.76 ± 0.86	43.81 ± 0.57

**Table 2:** MTT assay of 7a–s against MDAMB231.

Compound	Percentage of inhibition against treated concentrations (µM)					IC_50_ (µg/mL)
	1	5	10	25	50	
**7a**	84.42 ± 0.35	55.22 ± 0.46	44.52 ± 0.29	24.96 ± 0.27	14.71 ± 0.42	13.82 ± 0.51
**7b**	86.22 ± 0.83	56.42 ± 0.47	46.91 ± 0.44	32.3 ± 0.16	19.03 ± 0.62	16.57 ± 0.63
**7c**	86.77 ± 0.96	57.39 ± 0.32	47.21 ± 0.93	38.69 ± 0.28	22.8 ± 0.89	18.75 ± 0.49
**7d**	85.23 ± 0.73	55.73 ± 0.21	45.64 ± 0.98	28.57 ± 0.43	16.83 ± 0.16	15.09 ± 0.12
**7e**	82.18 ± 0.79	52.86 ± 0.75	42.09 ± 0.52	15.83 ± 0.14	11.33 ± 0.22	10.86 ± 0.38
**7f**	83.22 ± 0.21	54.18 ± 0.35	43.66 ± 0.54	23.33 ± 0.26	13.75 ± 0.73	12.87 ± 0.23
**7g**	81.56 ± 0.48	52.28 ± 0.86	41.70 ± 0.74	14.62 ± 0.34	10.61 ± 0.28	10.34 ± 0.16
**7h**	82.62 ± 0.41	53.75 ± 0.75	42.44 ± 0.49	21.39 ± 0.29	12.60 ± 0.72	12.05 ± 0.37
**7i**	84.78 ± 0.64	55.71 ± 0.32	45.62 ± 0.94	28.55 ± 0.30	16.82 ± 0.37	14.98 ± 0.65
**7j**	87.89 ± 0.95	58.44 ± 0.88	48.17 ± 0.88	39.53 ± 0.23	23.29 ± 0.98	19.59 ± 0.41
**7k**	97.31 ± 0.74	88.16 ± 0.43	80.54 ± 0.54	70.48 ± 0.17	41.54 ± 0.91	42.25 ± 0.36
**7l**	95.13 ± 0.85	82.38 ± 0.27	75.26 ± 0.81	65.86 ± 0.47	38.81 ± 0.88	38.86 ± 0.28
**7m**	92.11 ± 0.88	71.15 ± 0.19	65 ± 0.46	56.88 ± 0.59	33.52 ± 0.57	31.85 ± 0.06
**7n**	90.57 ± 0.59	69.83 ± 0.31	61.97 ± 0.44	54.23 ± 0.32	31.96 ± 0.16	29.58 ± 0.75
**7o**	94.97 ± 0.73	76.63 ± 0.13	70.00 ± 0.93	61.26 ± 0.29	36.1 ± 0.64	35.05 ± 0.74
**7p**	89.82 ± 0.36	68.33 ± 0.79	61.77 ± 0.42	51.43 ± 0.36	30.31 ± 0.84	28.24 ± 0.62
**7q**	93.46 ± 0.54	72.41 ± 0.45	66.15 ± 0.69	57.89 ± 0.12	34.12 ± 0.37	32.71 ± 0.87
**7r**	96.33 ± 0.48	86.31 ± 0.21	78.85 ± 0.32	69.00 ± 0.05	40.66 ± 0.86	41.19 ± 0.94
**7s**	88.63 ± 0.67	59.87 ± 0.26	49.22 ± 0.24	43.07 ± 0.28	25.38 ± 0.46	21.38 ± 0.32
**Doxorubicin**	93.65 ± 0.42	87.54 ± 0.67	79.98 ± 0.67	69.98 ± 0.47	41.25 ± 0.79	42.08 ± 0.26

**Table 3:** MTT assay of 7a–s against HEK293T.

Compound	Percentage of inhibition against treated concentrations (µM)					IC_50_ (µg/mL)
	1	5	10	25	50	
**7a**	99.93 ± 0.11	98.52 ± 0.96	97.63 ± 0.45	96.81 ± 0.20	57.05 ± 0.81	65.53 ± 0.58
**7b**	98.93 ± 0.66	97.41 ± 0.57	96.44 ± 0.46	95.2 ± 0.29	55.11 ± 0.88	62.88 ± 0.45
**7c**	96.63 ± 0.53	94.28 ± 0.53	90.18 ± 0.38	83.28 ± 0.21	49.08 ± 0.23	52.51 ± 0.61
**7d**	99.76 ± 0.56	98.09 ± 0.42	97.1 ± 0.88	96.35 ± 0.19	56.78 ± 0.69	65.13 ± 0.44
**7e**	98.73 ± 0.97	97.08 ± 0.25	95.2 ± 0.76	91.72 ± 0.35	54.05 ± 0.77	60.22 ± 0.52
**7f**	92.96 ± 0.52	90.92 ± 0.52	88.07 ± 0.68	80.53 ± 0.41	47.46 ± 0.38	50.86 ± 0.39
**7g**	95.9 ± 0.54	92.62 ± 0.31	88.84 ± 0.07	81.24 ± 0.32	48.22 ± 0.86	51.22 ± 0.17
**7h**	92.81 ± 0.18	89.61 ± 0.31	86.2 ± 0.66	78.45 ± 0.18	46.23 ± 0.43	49.02 ± 0.12
**7i**	98.39 ± 0.19	95.73 ± 0.97	93.38 ± 0.64	88.56 ± 0.24	52.19 ± 0.26	57.14 ± 0.33
**7j**	96.71 ± 0.63	94.47 ± 0.83	91.28 ± 0.48	84.17 ± 0.37	49.6 ± 0.38	53.32 ± 0.47
**7k**	99.55 ± 0.46	97.77 ± 0.09	96.79 ± 0.17	95.46 ± 0.43	56.26 ± 0.12	64.18 ± 0.55
**7l**	96.44 ± 0.31	94.13 ± 0.23	89.65 ± 0.12	82.83 ± 0.54	48.82 ± 0.67	52.15 ± 0.60
**7m**	97.18 ± 0.28	94.73 ± 0.82	92.36 ± 0.46	86.88 ± 0.30	51.2 ± 0.07	55.84 ± 0.43
**7n**	96.84 ± 0.75	94.67 ± 0.59	92.02 ± 0.39	84.32 ± 0.28	49.69 ± 0.21	53.43 ± 0.36
**7o**	95.33 ± 0.63	91.17 ± 0.26	88.65 ± 0.11	80.68 ± 0.76	48.18 ± 0.79	51.18 ± 0.94
**7p**	96.23 ± 0.74	93.99 ± 0.91	89.17 ± 0.81	82.34 ± 0.34	48.53 ± 0.12	51.74 ± 0.87
**7q**	98.51 ± 0.12	96.21 ± 0.72	94.69 ± 0.34	91.34 ± 0.41	53.83 ± 0.58	60.01 ± 0.73
**7r**	97.98 ± 0.88	95.25 ± 0.68	92.81 ± 0.21	88.11 ± 0.25	51.93 ± 0.23	56.85 ± 0.67
**7s**	93.26 ± 0.88	91.78 ± 0.53	88.09 ± 0.54	80.57 ± 0.26	47.79 ± 0.79	51.06 ± 0.15
**Doxorubicin**	96.1 ± 0.83	95.66 ± 0.42	92.44 ± 0.56	85.27 ± 0.78	50.25 ± 0.14	54.38 ± 0.49

### Molecular docking studies

The mechanistic inhibition of cancer cell proliferation in vitro by **7a–s** has been arbitrated by molecular docking interactions studies. In the course, the crystal structures of proteins were acquired as PDB files from protein data bank and considered after removal of bound water, ligands and cofactors from the environment. The .pdb files and .mol2 files of **7a–s** were produced from Chem3D Pro 14.0 of ChemBioOffice software and docked with the selected proteins on Swiss Dock^37^. In view of prompt concerns of anti-cancer potentiality, study has been protracted to the standard Doxorubicin.

The interactive structures were captured in energy minimized optimizations with 0.100 of minimum root mean standard deviation gradient and the binding modes are envisaged by UCSF Chimera^38^. The binding energies of proper interactions of title compound ligands with target protein receptors in chain P of 4N5Y (hemagglutinin HA1 chain) for HT29, chain B of 1IGT (IGG2A intact antibody—MAB231) for MDAMB231 and chain A of 2VWD (hemagglutinin-neuraminidase) for HEK293T cancer cell lines were considered and fond that **7a–s** are effectively bound with the identified proteins as presented in Tables 4, 5, 6 and 7.

**Table 4:** Molecular docking interactions of 7a–s with Chain P of 4N5Y protein of HT29 cancer cell lines.

Compound	Cluster number		Cluster rank	Binding energy (KCal/mol)	No. of H. bonds	H-bond ligand atoms	H-bond receptor atoms	Binding interaction	Bond length (A°)	H-bond type
7a	0		0	− 8.6095	2	2	2	Ligand(C=O)—GLY23(HN)	2.8935	Donor
								Ligand(Pyr-N)—HSD25(HN)	2.9687	Donor
7b	0		3	− 8.4816	2	2	2	Ligand(C=O)—GLY23(HN)	2.8223	Donor
								Ligand(Pyr-N)—HSD25(HN)	2.9202	Donor
7c	0		7	− 7.2184	2	2	2	Ligand(C=O)—GLY23(HN)	3.0877	Donor
								Ligand(C=O)—HSD25(HN)	1.9558	Donor
7d	0		10	− 8.4880	2	2	2	Ligand(C=O)—GLY23(HN)	3.0911	Donor
								Ligand(C=O)—PHE138(HN)	3.5888	Donor
7e	1		15	− 8.2767	2	2	2	Ligand(C=O)—GLY23(HN)	3.1952	Donor
								Ligand(Pyr-N)—HSD25(HN)	2.8128	Donor
7f	0		0	− 8.8058	2	2	2	Ligand(C=O)—GLY23(HN)	2.8721	Donor
								Ligand(Pyr-N)—HSD25(HN)	2.9898	Donor
7g	0		5	− 8.7595	2	2	2	Ligand(C=O)—GLY23(HN)	3.1550	Donor
								Ligand(C=O)—HSD25(HN)	2.8423	Donor
7h	0		7	− 8.5984	2	2	2	Ligand(C=O)—GLY23(HN)	2.8549	Donor
								Ligand(Thiop-S)—HSD25(HN)	3.6470	Donor
7i	0		3	− 8.8206	2	2	2	Ligand(C=O)—GLY23(HN)	2.9845	Donor
								Ligand(C=O)—PHE138(HN)	3.4200	Donor
7j	0		1	− 8.3752	2	2	2	Ligand(C=O)—GLY23(HN)	2.9582	Donor
								Ligand(Thiaz-N)—HSD25(HN)	2.8874	Donor
7k	0		3	− 8.3791	2	2	2	Ligand(C=O)—GLY23(HN)	3.4829	Donor
								Ligand(C=O)—PHE138(HN)	3.3366	Donor
7l	3		0	− 7.8424	2	2	2	Ligand(C=O)—GLY23(HN)	2.1122	Donor
								Ligand(C=O)—ALA7(HN)	2.8755	Donor
7m	2		3	− 8.4478	2	2	2	Ligand(C=O)—GLY23(HN)	1.9981	Donor
								Ligand(C=O)—ALA7(HN)	2.7552	Donor
7n	1		2	− 8.4168	2	2	2	Ligand(C=O)—GLY23(HN)	2.1058	Donor
								Ligand(C=O)—ALA7(HN)	2.8589	Donor
7o	0		18	− 9.0887	2	2	2	Ligand(C=O)—GLY23(HN)	3.0123	Donor
								Ligand(C=O)—PHE138(HN)	3.1354	Donor
7p	0		20	− 8.1701	2	2	2	Ligand(C=O)—GLY23(HN)	2.9133	Donor
								Ligand(C=O)—PHE138(HN)	3.4498	Donor
7q	1		3	− 8.4636	2	2	2	Ligand(C=O)—GLY23(HN)	3.9704	Donor
								Ligand(C=O)—PHE138(HN)	3.3830	Donor
7r	1		2	− 8.4434	2	2	2	Ligand(C=O)—GLY23(HN)	3.9253	Donor
								Ligand(C=O)—PHE138(HN)	3.3466	Donor
7s	0		0	− 8.3221	2	2	2	Ligand(C=O)—GLY23(HN)	2.5952	Donor
								Ligand(Pyr-N)—PHE138(HN)	3.0020	Donor
Doxorubicin	5		3	− 8.1864	4	4	4	Ligand(HO)—GLY23(HN)	2.6571	Donor
								Ligand(OH)—GLY23(O=C)	1.8366	Donor
								Ligand(HO)—HSD25(HN)	2.7971	Donor
								Ligand(C=O)—PHE138(HN)	2.4472	Donor

**Table 5:** Molecular docking interactions of 7a–s with Chain B of 1IGT protein of MDAMB231 cancer cell lines.

Compound	Cluster number	Cluster rank	Binding energy (KCal/mol)	No. of H. bonds	H-bond ligand atoms	H-bond receptor atoms		Binding interaction	Bond length (A°)	H-bond type
7a	0	5	− 7.4821	2	2	2		Ligand(C=O)—TYR35(HO)	2.1563	Donor
								Ligand(Pyr-N)—TYR58(HO)	4.0539	Donor
7b	7	0	− 7.2520	2	1	2		Ligand(C=O)—ILE260(HN)	4.1297	Donor
								Ligand(C=O)—LYS261(HN)	4.3719	Donor
7c	13	4	− 7.4102	2	1	2		Ligand(C=O)—ILE260(HN)	4.3841	Donor
								Ligand(C=O)—LYS261(HN)	4.6212	Donor
7d	13	0	7.3449	2	1	2		Ligand(C=O)—ILE260(HN)	3.6546	Donor
								Ligand(C=O)—LYS261(HN)	4.1198	Donor
7e	12	0	− 7.4886	2	1	2		Ligand(C=O)—ILE260(HN)	4.1192	Donor
								Ligand(C=O)—LYS261(HN)	4.3945	Donor
7f	7	6	− 7.4606	2	1	2		Ligand(C=O)—ILE260(HN)	4.1971	Donor
								Ligand(C=O)—LYS261(HN)	4.5621	Donor
7g	4	1	− 7.3478	2	1	2		Ligand(C=O)—ILE260(HN)	4.1345	Donor
								Ligand(C=O)—LYS261(HN)	4.4337	Donor
7h	24	0	− 7.2976	2	1	2		Ligand(C=O)—THR108(HO)	3.3480	Donor
								Ligand(C=O)—GLU150(HN)	3.7851	Donor
7i	19	4	− 7.6623	2	1	2		Ligand(C=O)—ILE260(HN)	4.0903	Donor
								Ligand(C=O)—LYS261(HN)	4.3806	Donor
7j	19	6	− 7.2855	2	1	2		Ligand(C=O)—ILE260(HN)	4.3141	Donor
								Ligand(C=O)—LYS261(HN)	4.5155	Donor
7k	8	4	− 7.3750	2	1	2		Ligand(C=O)—ILE260(HN)	4.0838	Donor
								Ligand(C=O)—LYS261(HN)	4.4159	Donor
7l	10	2	− 7.2863	2	1	2		Ligand(C=O)—ILE260(HN)	4.212	Donor
								Ligand(C=O)—LYS261(HN)	4.3262	Donor
7m	10	1	− 7.4682	2	1	2		Ligand(C=O)—ILE260(HN)	4.2244	Donor
								Ligand(C=O)—LYS261(HN)	4.4613	Donor
7n	12	0	− 7.5436	2	1	2		Ligand(C=O)—ILE260(HN)	4.1754	Donor
								Ligand(C=O)—LYS261(HN)	4.4298	Donor
7o	7	2	− 7.6293	2	1	2		Ligand(C=O)—ILE260(HN)	4.1210	Donor
								Ligand(C=O)—LYS261(HN)	4.4333	Donor
7p	8	1	− 7.3795	2	1	2		Ligand(C=O)—ILE260(HN)	4.1400	Donor
								Ligand(C=O)—LYS261(HN)	4.4103	Donor
7q	10	0	− 7.3624	2	1	2		Ligand(C=O)—ILE260(HN)	4.1312	Donor
								Ligand(C=O)—LYS261(HN)	4.4088	Donor
7r	7	1	− 7.4395	2	1	2		Ligand(C=O)—ILE260(HN)	4.1234	Donor
								Ligand(C=O)—LYS261(HN)	4.3922	Donor
7s	13	2	− 7.1704	2	1	2		Ligand(C=O)—ILE260(HN)	4.3825	Donor
								Ligand(C=O)—LYS261(HN)	4.5276	Donor
Doxorubicin	12	2	− 7.2134	4	4	4		Ligand(C=O)—ILE260(HN)	3.5160	Donor
								Ligand(C=O)—LYS261(HN)	3.7258	Donor
								Ligand(HO)—VAL402(HN)	2.1897	Donor
								Ligand(OH)—ASN421(O=C)	2.1975	Acceptor

**Table 6:** Molecular docking interactions of 7a–s with Chain A of 2VWD protein of HEK293T cancer cell lines.

Compound	Cluster number	Cluster rank	Binding energy (KCal/mol)	No. of H. bonds	H-bond ligand atoms	H-bond receptor atoms	Binding interaction	Bond length (A°)	H-bond type
7a	10	4	− 7.6705	2	2	2	Ligand(C=O)—GLU226(HN)	3.2728	Donor
							Ligand(Pyr-N)—LEU567(HN)	4.0462	Donor
7b	7	0	− 7.2520	2	1	2	Ligand(C=O)—LYS569(HN)	4.2550	Donor
							Ligand(C=O)—ASN570(HN)	2.5205	Donor
7c	7	0	− 7.1106	2	1	2	Ligand(Pyr-N)—LEU448(HN)	2.9792	Donor
							Ligand(Pyr-N)—GLY449(HN)	3.7353	Donor
7d	0	0	− 7.2749	2	2	2	Ligand(C=O)—GLY227(HN)	2.4572	Donor
							Ligand(C=O)—LYS569(HN)	4.1546	Donor
7e	13	5	− 7.5182	2	2	2	Ligand(Pyr-N)—GLY227(HN)	2.3756	Donor
							Ligand(C=O)—LYS569(HN)	2.3751	Donor
7f	13	1	− 7.3895	2	2	2	Ligand(Pyr-N)—GLY227(HN)	4.1946	Donor
							Ligand(C=O)—LEU448(HN)	4.0997	Donor
7g	2	3	− 7.3397	2	2	2	Ligand(C=O)—GLY227(HN)	3.1459	Donor
							Ligand(C=O)—LEU448(HN)	3.1550	Donor
7h	0	4	− 7.4982	2	2	2	Ligand(Thiop-S)—ASP257(HN)	3.7906	Donor
							Ligand(C=O)—LYS569(HN)	2.8464	Donor
7i	3	7	− 8.1394	2	2	2	Ligand(Pyr-N)—MET224(HN)	4.3168	Donor
							Ligand(C=O)—LEU567(HN)	3.0713	Donor
7j	0	0	− 7.5193	2	1	2	Ligand(Thiaz-S)—ARG435(HN)	3.6283	Donor
							Ligand(Thiaz-S)—LYS465(HN)	4.3091	Donor
7k	17	0	− 7.3786	2	2	2	Ligand(HO)—GLY214(HN)	2.3554	Donor
							Ligand(C=O)—LYS591(HN)	1.8494	Donor
7l	6	2	− 7.3860	2	2	2	Ligand(C=O)—GLY227(HN)	2.3839	Donor
							Ligand(C=O)—LYS569(HN)	4.1170	Donor
7m	9	6	− 7.2792	2	1	2	Ligand(C=O)—CYS395(S)	5.2391	Donor
							Ligand(C=O)—LEU436(HN)	2.8589	Donor
7n	3	7	− 7.4252	2	1	2	Ligand(C=O)—ARG435(HN)	4.0520	Donor
							Ligand(C=O)—LYS465(HN)	4.6849	Donor
							Ligand(C=O)—ARG435(HN)	3.2132	Donor
7p	1	1	− 7.1904	2	1	2	Ligand(C=O)—ARG435(HN)	2.0468	Donor
							Ligand(C=O)—ARG435(HN)	3.2292	Donor
7q	24	1	− 7.3226	2	1	2	Ligand(C=O)—ARG548(HN)	1.8235	Donor
							Ligand(C=O)—ARG548(HN)	3.1213	Donor
7r	4	4	− 7.5366	2	2	2	Ligand(C=O)—GLY227(HN)	2.4060	Donor
							Ligand(C=O)—LYS569(HN)	4.1669	Donor
7s	4	0	− 6.9915	2	1	2	Ligand(Pyr-N)—LYS569(HN)	2.1221	Donor
							Ligand(Pyr-N)—ASN570(HN)	4.4184	Donor
Doxorubicin	4	2	− 8.1568	4	4	3	Ligand(HO)—ARG435(HN)	2.6447	Donor
							Ligand(NH)—THR498(O=C)	2.5981	Acceptor
							Ligand(OH)—ASP461(OCOH)	2.0155	Acceptor
							Ligand(NH)—ASP461(OCOH)	2.0294	Acceptor

**Table 7:**
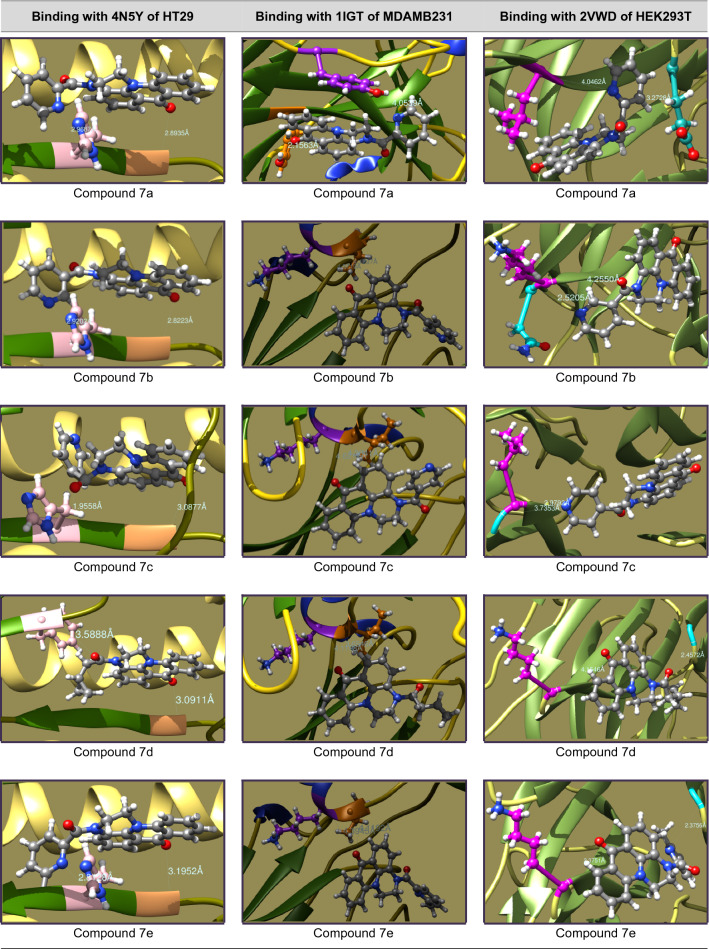

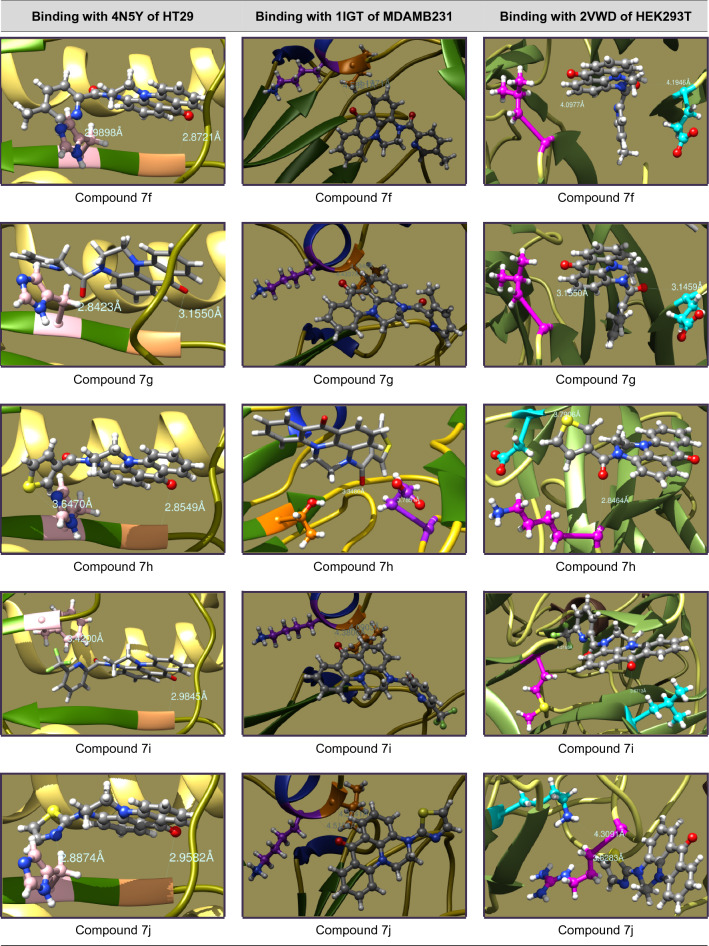

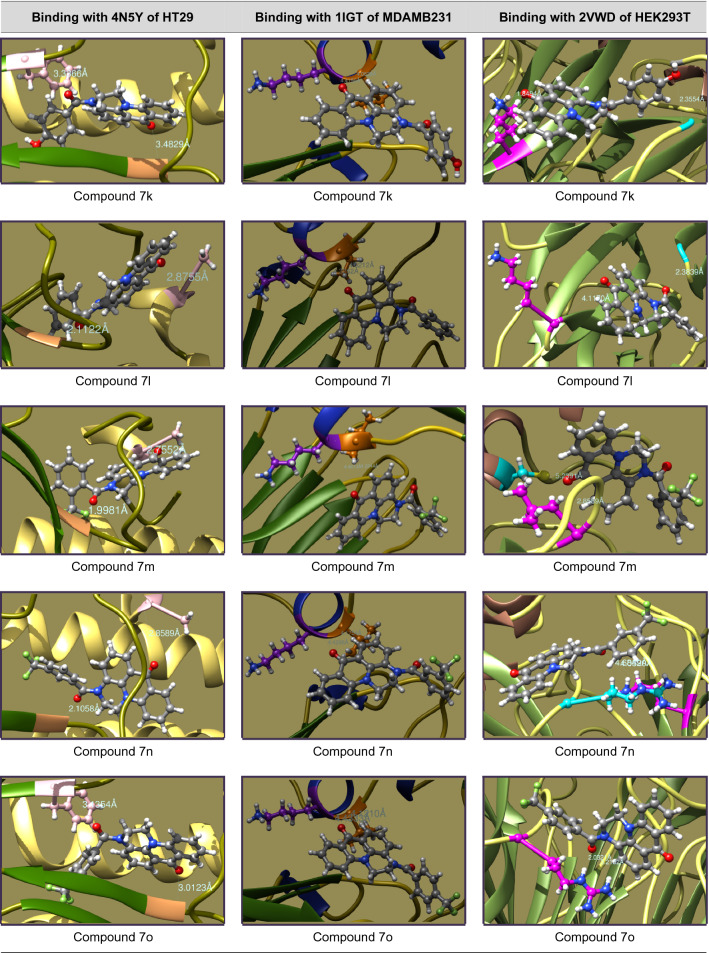

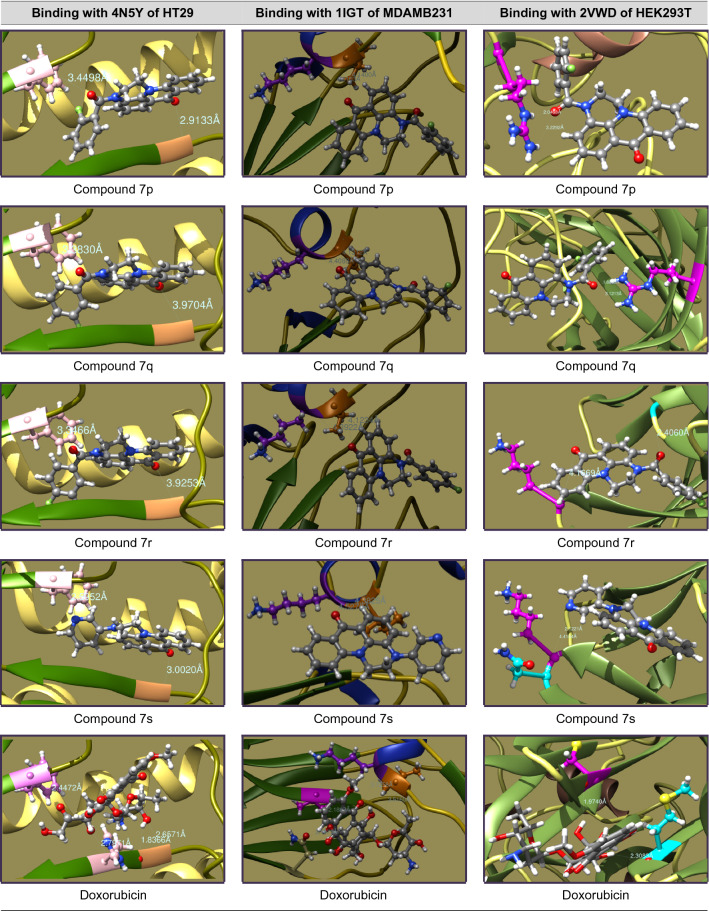
Potential protein–ligand binding interactions of compounds 7a–s and doxorubicin with identified enzymatic proteins.

### ADMET properties^39^

ADMET properties of **7a–s** have calculated on preADMET online server^40^, which assisted to understand their ADMET potentialities In continuance, the prediction of tumarogenic, mutagenic, irritant and reproductive effects have assisted to ascertain the toxicity properties. All the ADMET properties were identified with in the potential limits of safe drugs as presented in Table 8.

**Table 8:** ADMET properties predicted for compounds **7a–s.**

Entry	In vivo blood–brain barrier penetration (C. brain/C. blood)^a^	In vitro Caco-2 cell permeability (nm/s)^b^	Human intestinal absorption (HIA, %)^c^	In vitro MDCK cell permeability (nm/s)^d^	In vitro plasma protein binding (%)^e^	Toxicity^f^
7a	2.5177	40.1997	97.3627	215.3520	98.4091	Negative
7b	1.7088	33.8868	97.3627	33.1032	94.5608	Negative
7c	0.6783	35.9472	97.3627	38.6052	95.3119	Negative
7d	0.6990	42.4307	97.8198	81.3338	89.9324	Negative
7e	2.4518	44.8823	97.3643	74.8252	97.2246	Negative
7f	2.9893	42.2071	97.3643	37.7509	96.8717	Negative
7g	2.4944	42.3708	97.3643	144.9730	96.9384	Negative
7h	2.6465	31.2874	97.6281	33.3530	89.4191	Negative
7i	0.1523	25.8135	97.3668	0.0526	97.6000	Negative
7j	0.9136	44.8966	98.7226	183.6750	92.4212	Negative
7k	0.3451	21.6738	96.2654	1.5542	92.0128	Negative
7l	3.7909	42.2821	97.9994	33.6899	95.9548	Negative
7m	0.3443	28.7804	98.0547	0.0890	91.5915	Negative
7n	0.1846	28.1250	98.0547	0.0454	94.6528	Negative
7o	0.2196	28.4536	98.0547	0.0483	89.0836	Negative
7p	2.8566	39.6531	98.0029	8.8231	91.9695	Negative
7q	2.4562	40.4987	98.0029	2.1707	90.7872	Negative
7r	1.8993	34.4524	97.3623	64.6242	94.1251	Negative
7s	1.7088	33.8868	97.3627	33.1032	94.5608	Negative
Doxorubicin	0.0328	17.7265	31.9529	1.0236	32.7895	Negative

^a^BBB (Blood–Brain Barrier) penetration = [Brain]/ [Blood]; ^b^Caco-2 cells derived from human colon adenocarcinoma, possessing multiple drug transport pathways through intestinal epithelium; ^c^HIA (Human intestinal absorption), the sum of absorption and bioavailability evaluated from ratio of excretion in urine, bile and feces etc*.*; ^d^MDCK cell system is used as tool for rapid permeability screening; ^e^% of drug that binds to plasma protein; ^f^*in vitro* Ames test by Metabolic and Non-metabolic activated TA100 and TA1535 strains collected from rat liver homogenate.

### QSAR studies

As ADMET properties are foremost requisites for drug candidates to reach clinical stage, in addition the oral bio-availability comprehends them by precise poise among partitioning and solubility as evolved from QSAR studies. Similarly, obeying Lipinski’s rule of five^41^ is a notable tool for screening potentiality of newer molecules and was predicted by Molinspiration^42^ software. The computation of Veber Rule and other parameters like partition coefficient (octanol to water) and percentage of absorption in addition fulfils the QSAR studies as presented the results in Table 9.

**Table 9:** QSAR properties of **7a–s.**

Entry	Lipinski parameters						Veber parameters			Other parameters					
	MW	HB Don	HB Acc	logP (o/w)	MR	Lip. Vio	TPSA	No. of RB	Veb. Vio	No. of H	V.Vol	ρ	S	CLP	% ABS
**7a**	341.37	5	0	1.63	96.38	0	55.21	1	0	15	298.16	1.44	− 5.55	3.48	89.95
**7b**	341.37	5	0	1.56	96.38	0	55.21	1	0	15	298.16	1.44	− 5.52	3.43	89.95
**7c**	341.37	5	0	1.51	96.38	0	55.21	1	0	15	298.16	1.44	− 5.52	3.43	89.95
**7d**	318.38	4	0	1.56	90.33	0	42.31	2	0	18	287.3	1.37	− 5.94	3.76	94.40
**7e**	355.40	5	0	1.68	101.01	0	55.21	1	0	17	314.72	1.41	− 5.92	3.88	89.95
**7f**	355.40	5	0	2.08	101.01	0	55.21	1	0	17	314.72	1.41	− 5.89	3.83	89.95
**7g**	355.40	5	0	2.41	101.01	0	55.21	1	0	17	314.72	1.41	− 5.89	3.83	89.95
**7h**	346.41	4	0	2.39	96.72	0	42.31	1	0	14	293.03	1.48	− 6.22	4.21	94.40
**7i**	409.37	5	0	2.59	101.37	0	55.21	2	0	14	329.46	1.53	− 6.35	4.38	89.95
**7j**	319.39	4	0	2.52	94.52	0	38.13	1	0	13	269.89	1.53	− 6.62	4.37	95.85
**7k**	356.38	5	1	2.32	100.11	0	62.54	1	0	16	310.33	1.48	− 6.02	4.08	87.42
**7l**	340.38	4	0	2.80	98.58	0	42.31	1	0	16	302.32	1.40	− 6.32	4.43	94.40
**7m**	408.38	4	0	3.65	103.57	0	42.31	2	0	15	333.61	1.49	− 7.1	5.28	94.40
**7n**	408.38	4	0	3.67	103.57	0	42.31	2	0	15	333.61	1.49	− 7.1	5.28	94.40
**7o**	408.38	4	0	3.69	103.57	0	42.31	2	0	15	333.61	1.49	− 7.1	5.28	94.40
**7p**	358.37	4	0	2.91	98.70	0	42.31	1	0	15	307.25	1.44	− 6.63	4.53	94.40
**7q**	358.37	4	0	2.94	98.70	0	42.31	1	0	15	307.25	1.44	− 6.63	4.53	94.40
**7r**	358.37	4	0	2.96	96.49	0	42.31	1	0	14	307.25	1.49	− 6.63	4.53	94.40
**7s**	313.36	4	0	2.39	96.38	0	38.13	1	0	15	279.18	1.44	− 6.09	3.47	95.85
**Doxorubicin**	543.52	12	7	0.57	131.52	3	206.08	5	0	29	459.18	1.61	− 4.51	0.17	37.90

MW: Molecular weight; HB Don: Hydrogen bond donors (n ON); HB Acc: Hydrogen bond acceptors (n OH NH); logP: log of octanol to water partition coefficient; MR: Molecular refractivity (cm^3^/mol); Lip. Vio.: Lipinski Violations; TPSA: Total polar surface area (A°)^2^; No. of RB: Number of rotatable bonds; Veb. Vio.: Veber Violations; No. of ‘H’: Number of Hydrophobic Atoms; V.Vol.: *Van der Waals* volume; ρ: Density (gm/cc); S: Solubility; CLP: ClogP; % ABS: % of absorption;

### Bioactivity and toxicity risk studies

The QSAR descriptors of **7a–s** have been predicted on molinspiration online server^42^ as properties were explored with molinspiration engine v2018.10 and bioactivity scores were explored with molinspiration engine v2018.03 and the results proved them as safer drugs. Similarly, the Osiris online property explorer toolkit^43^ has provided the toxicity risks and drug properties as presented in Table 10. This study helped in understanding the physico-chemical interactions of the synthesized compounds against their targets and eventually facilitated in determining their drug properties.

**Table 10:** Bioactivity scores, drug properties and toxicity risks of 7a–s.

Entry	Bioactivity						Drug properties		Toxicity risks			
	GPCRL	ICM	KI	NRL	PI	EI	Drug-likeness	Drug score	Mut	Tum	Irrit	R.E
**7a**	0.24	− 0.09	0.04	− 0.28	− 0.01	0.23	4.82	0.58	Nil	Nil	Nil	Nil
**7b**	0.22	− 0.10	0.12	− 0.26	− 0.11	0.19	5.94	0.59	Nil	Nil	Nil	Nil
**7c**	0.19	− 0.12	0.12	− 0.25	− 0.11	0.17	5.11	0.58	Nil	Nil	Nil	Nil
**7d**	0.32	− 0.14	0.01	− 0.16	0.04	0.18	5.32	0.54	Nil	Nil	Nil	Nil
**7e**	0.21	− 0.15	− 0.04	− 0.30	− 0.05	0.16	4.64	0.52	Nil	Nil	Nil	Nil
**7f**	0.19	− 0.16	− 0.02	− 0.29	− 0.08	0.16	4.64	0.52	Nil	Nil	Nil	Nil
**7g**	0.23	− 0.09	− 0.04	− 0.27	− 0.02	0.18	4.71	0.52	Nil	Nil	Nil	Nil
**7h**	0.16	− 0.22	− 0.02	− 0.34	− 0.18	0.06	5.72	0.29	Risk	Nil	Nil	Nil
**7i**	0.23	− 0.02	− 0.02	− 0.14	0.00	0.21	− 2.49	0.23	Nil	Nil	Nil	Nil
**7j**	− 0.01	− 0.36	0.02	− 0.46	− 0.36	0.24	4.37	0.45	Nil	Nil	Nil	Nil
**7k**	0.18	− 0.14	0.04	− 0.09	− 0.13	0.15	5.05	0.50	Nil	Nil	Nil	Nil
**7l**	0.15	− 0.18	0.01	− 0.22	− 0.14	0.11	4.73	0.46	Nil	Nil	Nil	Nil
**7m**	0.18	− 0.09	0.06	− 0.04	− 0.11	0.11	− 6.11	0.17	Nil	Nil	Nil	Nil
**7n**	0.19	− 0.09	0.05	− 0.07	− 0.10	0.10	− 5.58	0.17	Nil	Nil	Nil	Nil
**7o**	0.18	− 0.09	0.05	− 0.08	− 0.10	0.10	− 5.99	0.17	Nil	Nil	Nil	Nil
**7p**	0.13	− 0.22	0.03	− 0.19	− 0.16	0.10	3.50	0.43	Nil	Nil	Nil	Nil
**7q**	0.16	− 0.18	0.04	− 0.19	− 0.14	0.09	0.86	0.37	Nil	Nil	Nil	Nil
**7r**	0.15	− 0.19	0.03	− 0.20	− 0.16	0.09	4.23	0.43	Nil	Nil	Nil	Nil
**7s**	0.27	0.06	0.17	− 0.10	− 0.06	0.25	3.93	0.52	Nil	Nil	Nil	Nil
**Doxorubicin**	0.20	− 0.20	− 0.07	0.32	0.67	0.66	7.19	0.33	Nil	Nil	Risk	Nil

GPCRL: G protein-coupled receptor ligand; ICM: Ion channel modulator; KI: Kinase inhibitor; NRL: Nuclear receptor ligand; PI: Protease inhibitor; EI: Enzyme inhibitor; Mut: Mutagenic; Tum: Tumorigenic; Irrit: Irritant; R.E.: Reproductive effect.

### BSA protein binding assay

The protein binding assay of title compounds was performed with Bovine serum albumin (BSA), a standard protein, to correlate their evaluated anticancer activity through mutual interactions, where such interactions made these anticancer agents as transportable in blood. The UV–Visible absorption spectroscopy has adapted to track the changes in the absorption bands induced by conformational change showing the formation of the protein bound compound. The binding constant K_b_ predicted from the BSA protein binding assay was given in Table 11.

**Table 11:** The BSA protein binding constants (K_b_) of 7a–s.

Entry	λ_max_ (nm)	K_b_ (M^−1^)	Entry	λ_max_ (nm)	K_b_ (M^−1^)
7a	280	1.3042 × 10^4^	7k	280	0.9450 × 10^4^
7b	280	1.2800 × 10^4^	7l	280	1.0085 × 10^4^
7c	280	1.1943 × 10^4^	7m	280	1.1029 × 10^4^
7d	280	1.2944 × 10^4^	7n	280	1.1813 × 10^4^
7e	280	1.4140 × 10^4^	7o	280	1.0175 × 10^4^
7f	280	1.4033 × 10^4^	7p	280	1.1926 × 10^4^
7g	280	1.5156 × 10^4^	7q	280	1.0876 × 10^4^
7h	280	1.3785 × 10^4^	7r	280	1.0792 × 10^4^
7i	280	1.5415 × 10^4^	7s	280	1.1661 × 10^4^
7j	280	1.2693 × 10^4^	Doxorubicin	280	0.7865 × 10^4^

In assay, prepared standard BSA protein solution [2.5 mg in 10.0 mL of Tris–HCl buffer (5 mM Tris–HCl + 10 mM NaCl @ pH = 7.4) was preserved at refrigeration conditions. Title compound solutions were incubated at room temperature for nearly 30 min before the process. Then the UV–Visible absorption spectra of title compounds of a conserved concentration of 25 μM in combination with prepared BSA solutions ranging from 5 to 500 μM were analyzed in the wavelength ranging from 200 to 400 nm. The UV–visible spectral studies were performed in a mixed solvent system (1:9 DMSO and Tris–HCl buffer) and absorption spectra were recorded by using 1-cm-path quartz cuvettes at room temperature. Then the Binding constant K_b_ is calculated from Benesi–Hildebrand equation shown below. Where, A_o_ and ε_f_ are the absorbance and molar extinction coefficients of title compounds in free form, A and ε_b_ are absorbance and molar extinction coefficients of respective title compounds bound with BSA protein.

$$\frac{{A}_{o}}{A-{A}_{o}}=\frac{{\varepsilon }_{f}}{\left({\varepsilon }_{b}-{\varepsilon }_{f}\right)}+\frac{{\varepsilon }_{f}}{\left({\varepsilon }_{b}-{\varepsilon }_{f}\right){K}_{b} \left[Analayte\right]}$$

## Results and discussion

### Chemistry

The acridone fused piperazino-carboxamide derivatives **(7a–s)** synthesized in the present study are novel as the piperazine ring has been constructed on the bridged carbon (linking ring A and B) rather simply fusing on ring A or C of basic acridone skeleton. This kind of construction has evolved some triazolo-, imidazolo- and pyrimidino-fused acridones so far, this piperazine fused acridone frame linking alkyl/ aryl moieties with amide linker is a novel accomplishment to the existing array of acridones. As Pd(OAc)_2_–Xantphos is used as a potential catalyst–ligand system for C–N coupling^44^, here we have extended the use of Cs_2_CO_3_–Pd(OAc)_2_–Xantphos system in producing the acridone ring by forming a C–N linkage through N-arylation process. The chemical structures of the synthesized compounds **(7a–s)** of the study were confirmed by IR, ^1^H, ^13^C and mass spectrometry analyses and all the spectral responses were observed in their expected standard range, and corresponding mass spectral fragments were identified as their isotopic and daughter ion peaks at expected m/z with significant intensities.

### Cytotoxic activity

Among the acridone derivatives **(7a–s)** screened for anticancer activity against human colorectal adenocarcinoma (HT29) cancer cell lines, **7l** and **7k** were identified to exert highest activity with 68.88 and 66.23 of percentage of inhibition respectively and **7m–r** exhibited moderate activity in the range of 59.26–54.13 percentage of inhibition. Similarly as screened against human breast adenocarcinoma (MDA-MB-231) cancer cell lines, **7k**, **7r**, **7l** and **7o** have demonstrated significant activity with 70.48, 69.00, 65.86 and 61.26 percentage of inhibition respectively and 7q, 7m, 7n and 7p have exhibited moderate activity in the range of 57.89–51.43 percentage of inhibition. Similarly as screened against human embryonic kidney (HEK-293T) cancer cell lines almost all the compounds have exhibited the most significant activity in terms of percentage of inhibition in the range of 96.81–78.45, where 7a, 7d, 7k, 7b, 7e and 7q have exhibited 96.81, 96.35, 95.46, 95.20, 91.72 and 91.34 of percentage of inhibition respectively and remaining compounds have exhibited the moderate activity in the range of 88.56–78.45 of percentage of inhibition. The potential compounds **7k, 7r**, **7l**, **7o**, **7a**, **7d**, **7b**, **7e, 7i** and **7q** having the substitutions like p-hydroxybenzoyl, p-fluorobenzoyl, benzoyl, p-(trifluoromethyl)benzoyl, picolinoyl, cyclopropyl-acetyl, 3-nicotinoyl, 6-methylpicolinoyl, trifluoromethylpicolinyl and 3-fluorobenzoyl groups on acridone fused piperazine moiety. The significant inhibition of title compounds is due to the greater electron releasing capacity, better intermolecular hydrogen bonding interactions, higher electronegativity, molecular volume and steric hindrance, which are making them to get interact with targeted cell lines and have boosted the pharmacokinetic, physicochemical, liphophilic, properties of the title compounds that led to the metabolic destruction of cells^45^. Significantly the tested profiles on HEK293T normal cell lines revealed them as safer compounds which are more potent against HT29 and MDA-MB-231 cell lines as shown in Tables 1, 2 and 3.

### Molecular docking studies

**Figure 2:**
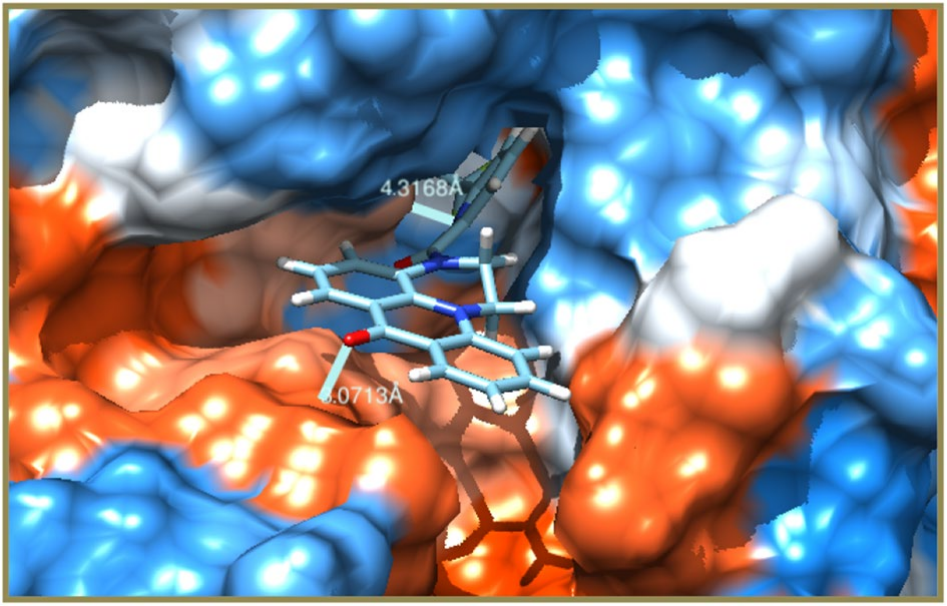
Hydrophobic surface protein–ligand interface in 7i.

The in vitro cell proliferation inhibition of **7a–s** against HT29, MDAMB231 and HEK293T has been supported by correlating the binding potentialities of **7a–s** with their corresponding enzymatic protein chains. In concern the molecular docking was performed for the structures of **7a–s** against chain P of 4N5Y (hemagglutinin HA1 chain) for HT29, chain B of 1IGT (IGG2A intact antibody—MAB231) for MDA-MB-231 and chain A of 2VWD (hemagglutinin-neuraminidase) for HEK293T cell lines^46^ to study the ligand–protein binding interactions. The binding interactions of aminoacid residues with hydrogen bond donors and acceptors of the **7a–s** and their docking postures were presented in Tables 4, 5, 6 and 7. The binding specificity of **7a–s** with the selected protein chains (4N5Y, 1IGT and 2VWD) that proliferates the cancerous cells growth will be arrested and acts as anticancer agents. As **7a–s** binds with chain P of 4N5Y for HT29, chain B of 1IGT for MDAMB231 and chain A of 2VWD for HEK293T cancer cell lines. Remarkably they have selectively bound to glycine (GLY), histidine (HSD), phenyl alanine (PHE), alanine (ALA) of 4N5Y with a binding energy ranging from − 9.0887 to − 7.2184 kcal/mol. Similarly bound to tyrosine (TYR), isoleucine (ILE), lysine (LYS), threonine (THR) and glutamic acid (GLU) of 1IGT with a binding energy ranging from − 7.6623 to − 7.1704 kcal/mol. Likewise bound to leucine (LEU), asparagine (ASN), methionine (MET), arginine (ARG), cysteine (CYS), glycine (GLY), lysine (LYS) and glutamic acid (GLU) of 2VWD with a binding energy ranging from − 8.1394 to − 6.9915 kcal/mol. The intense analysis discloses that neutral aminoacids are strongly bound to the ligands than basic than acidic aminoacids. The hydrophobic surface protein–ligand interfacial interactions in **7i** have been shown in Fig. 2.

### ADMET properties

The investigation of ADMET properties for a group of analytes under study assists to comprehend the physico-chemical interactions of those analytes and helps us to evaluate their drug-likeness properties. This type of high-throughput screening helps in distinguishing a lead compound of a large group in the fascinated domain of a target^47^. This critical study assists in identifying the pharmacokinetic properties of **7a–s** and to unveil their drug-like interactions. The Human intestinal absorption describes carrying of the effective composites to the target cell tissues via blood stream and made them to interact mutually. In oral administration of a drug-like compound its degree of absorption has been considered, where it again depends on its inherent bioavailability properties. There the absorbed quantity of compound be itself distributed into the muscles and there to other organs by the circulation via extracellular sites. Then the compound’s distribution lowers its plasma concentration independently and then metabolizes, from there those metabolites will be distributed by the enzymatic redox reactions. In pharmacological aspects, potential metabolites distributed will work proficiently on cellular systems, inactive metabolites deactivates the administered compound and diminishes its effect in vivo, and the inert metabolites will be automatically excreted from kidneys.

The analysis of the obtained ADMET properties (Table 8) of **7a–s** informed that the in vivo BBB penetration potentiality ratio is effective with a range of 0.1523–3.7909 and confirms their high CNS significance and approves their greater permeability permeability for the self-distribution in vivo. It is strengthened on the basis of their in vitro Caco-2 cell permeability perceived with 21.6738–44.8966 nm/s range, which institutes their persistent permeability to bind with plasma proteins and to penetrate in to BBB system. The in vitro PPB affinity in 89.0836–98.4091% range authorizes the robust binding capability of the compounds to plasma proteins. The in vitro MDCK cell permeability in 0.0454–215.3520 nm/s range reveals them as good permeable. Similarly the %HIA in the range of 96.2654–98.7226 ratio assures their interactions with the proper species in the anticipating target of domains. The negative sign of the toxicity predictions indicate that compounds **7a–s** are non-toxic and safer drugs. Inclusively this ADMET analysis has been revealed the potential physico-chemical interactions of **7a–s** and their drug-likeness properties.

### QSAR studies

QSAR results (Table 9) indicate that analogues **7a–s** under study with molecular weights ranging from 409.37 to 313.36 (less than 500 daltons) demonstrated log *P* in the range of 3.69 to 1.51 (less than 5) suggesting their better permeability through cell membranes. Similarly number of hydrogen bond acceptor and donors are in the line of Lipinski’s rule as < 10 and < 5 respectively. The molecular refractivity from 90.33 to 103.57 cm^3^/mol as in the standard range *i.e.,* 40–130 cm^3^/mol ascertains as all analogues are obeying the Lipinski rule of five and all they are considerably orally active drugs with good drug likeness properties. Moreover the total polar surface area contributed by the sum of polar atoms such as oxygen, nitrogen and attached hydrogens^48^, which is ranging from 38.13 to 62.54 is also obeying the Veber rule as it is less than 140 Å^2^. Hence, these molecules are estimated to be easily diffused, absorbed and transported. Here the total polar surface area is very much correlated with the hydrogen bonding of a molecule and is associated with the transport properties of drug across the membranes, prediction in the BBB and intestinal crossing. Molecules with total polar surface area in the range ≤ 160 Å^2^ have good intestinal absorption and ≤ 60 Å^2^ has BBB penetration^49^. Total polar surface area for the examined successions all the offshoots have come out to be best intestinal absorbers. On the other hand the number of rotatable bonds in all the compounds are limited to 1–2 which is as per the Veber rule (*i.e.,* less than 7) and hence in total they also obeying the Veber’s rule and again ascertains them as orally administrable drugs. Furthermore, the percentage of absorption ranging from 87.42 to 95.85, density in the range of 1.37 to 1.53 gm/cc, solubility ranging from − 5.52 to − 7.10, *Van der Waals* volume in the range 269.89 to 333.61 Å^3^ and ClogP in the range of 3.43 to 5.28 ascertains all the compounds as significantly safer drug-like compounds. These calculations are in understanding the physico-chemical interactions of synthesized analogues with their targets and eventually helped in determining their drug properties by associating with the bioactivity and toxicity risks studies.

### Bioactivity and toxicity risk studies

The prediction of bioactivity and toxicity risk studies of the synthesized analogues **7a–s** (Table 10) revealed their bioactivity properties like GPCR ligand property, ion channel modulator, kinase inhibitor, nuclear receptor ligand interactions, protease inhibitor and enzyme inhibitor interactions, and drug properties like drug-likeness and drug scores have measured and ascertained as potential non-toxic molecules. This Molinspiration prediction extensively helps to investigate the cheminformatics of the compounds under study by correlating with the database of in vitro and in vivo studies of established drugs based on mutual functional group similarity.

The toxicity risk results clearly indicate that **7a–s** are safer as showing low or no risks of mutagenicity, tumorigenicity, irritant and low or no effect on reproductive system and conformed drug like behavior. The positive value of drug likeness states that the molecule contains predominantly fragments which are frequently present in commercial drugs whereas majority of compounds accounts for negative values^38^. Solubility is an important factor which aids in the movement of a compound from the site of administration into the blood stream and poor solubility leads to poor absorption^50^. Alike drug score is a harmonizing parameter of druglikeness, ClogP, logS, molecular weight and toxicity risks and used to judge the compound's overall potential to qualify for a drug. Ultimately it is predicted that all the synthesized analogues **7a–s** exhibited higher scores than the standard drug.

### Structure activity relationship studies

**Figure 3:**
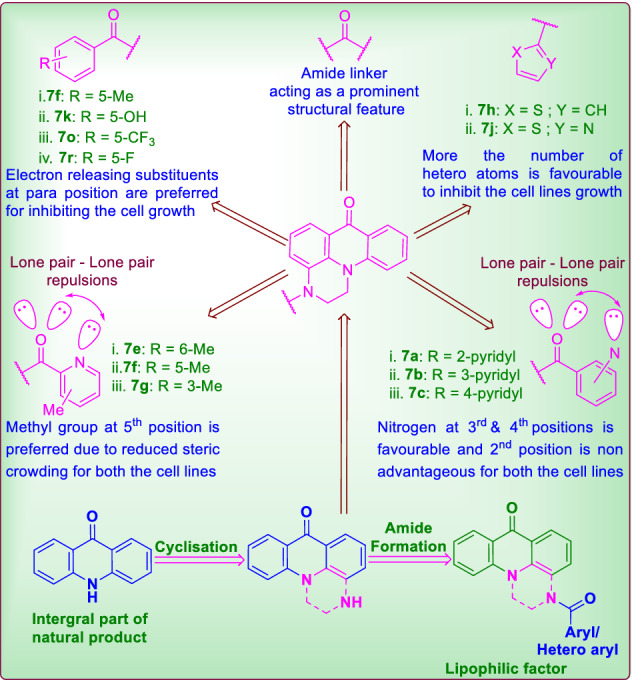
SAR and structural effect of amide bond on **7a–s.**

The comparative structural analysis infers that amide linker is beneficial as it highly elevated the activity of all the compounds. In over-all increase in number of hetero atoms in **7h** (thiophene) and **7j** (thiazole) increased the activity among the tested array of cells. Similarly among the positional isomers **7a** (2-pyridyl), **7b** (3-pyridyl) and **7c** (4-pyridyl), nitrogen atom at second position in **7a** is diminishing the activity and its lower commotion is due to lone pair-lone pair repulsions present on the oxygen and nitrogen (Fig. 3). Ultimately, nitrogen at fourth and third positions is more favourable for stimulating the activity. Similarly, isomer series of **7m** (*o*-CF_3_), **7n** (*m*-CF_3_) and **7o** (*p*-CF_3_) and **7p** (*o*-F), **7q** (*m*-F) and **7r** (*p*-F), have followed the activity trending in the order of meta > para > ortho by following the negative inductive effect and demonstrated the higher activity for meta position over other two. In the series of **7e–g**, the increasing order of activity is observed as **7g** (3-methylpicolinoyl) < **7e** (6-methylpicolinoyl) < **7f** (5-methylpicolinoyl) as di functional substitutions diminishing the activity based on steric factors. In brief, the para substituted compounds with respect to the amide bond are acting as electron releasing groups and improving the activity (Fig. 3).

### BSA protein binding assay

The study of BSA protein binding interactions of a drug substance infers about its possible transportability with BSA, as it is a standard protein carrier for almost all drugs and metabolites. Here, all the title compounds were identified as potentially bound to the BSA protein, compounds **7i, 7g, 7e** and **7f** having 2-trifluoromethyl, 2-methyl, 5-methyl and 4-methyl substitutions on picolinoyl groups were exhibited the highest binding constant (K^b^) values (Table 11) and absorption maxima at 280 nm and showed the hyperchromic effect. The values for these ranging from 1.5415 × 10^4^ M^−1^ to 1.4033 × 10^4^ M^−1^ are better in comparison to the Doxorubicin drug standard (0.7865 × 10^4^ M^−1^) of the study. Hence, the BSA protein binding assay also ascertained the drug properties of the title compounds along the cytotoxic activity screened for them.

## Conclusion

An array of novel tetracyclic acridone derivatives **(7a–s)** have been synthesized in good yields from quinoxaline, where Cs_2_CO_3_–Pd(OAc)_2_–Xantphos used as a potential catalytic system in generating the acridone ring by forming a C–N linkage. Similarly, the anticancer activity evaluation had revealed that **7k, 7r**, **7l**, **7o**, **7a**, **7d**, **7b**, **7e, 7i** and **7q** were identified as effective anticancer agents as assessed by MTT assay against HT29, MDAMB231 and HEK293T cancer cell lines. In addition, the molecular docking studies, QSAR, ADMET, bioactivity and toxicity risk properties predicted for them have strengthened their drug-likeness and were well correlated with in vitro anticancer activity and BSA protein binding assay results. The BSA binding studies confirms the stronger bond of title compounds with BSA as evidenced from the binding constants ranging from 1.5415 × 10^4^ M^−1^ to 1.4033 × 10^4^ M^−1^ than the Doxoruicin drug reference. The molecular docking studies have inferred that compounds **7a–s** have potentially bound to Glycine and Lysine (neutral aminoacids) present on enzymatic proteins 4N5Y, 1IGT and 2VWD with a binding energy ranging from − 9.0887 to − 6.9915 kcal/mol. These potential inter molecular hydrogen bonding interactions between hydrogen atoms of amides of enzymatic proteins and carbonyl groups of acridinone and 1-piperazinoyl fragments are responsible for cell growth inhibition by **7a–s**. Ultimately, compounds **7a–s** were identified as potential excitatory protein donor antagonists to reduce and block the cell functionality by neuronal damages and death of the cells. Therefore the idea of fusing acridone core with piperazine ring and linking various alkyl/ aryl/ heteroaryl to them via piperzinoyl carbon has been ascertained as an admiring task in designing these potential anticancer agents. Prospectively this study is serving as a trustworthy tool in accomplishing more potential acridone derivatives by novel structural modifications in **7a–s**, which are under study and are exercising for higher activity. These results demonstrate strong evidence for innovative investigations involving in designing more analogous compounds and methodologies to explore mechanistic aspects of their anticancer activity.

## Supplementary information

Supplementary Information.
