# Supplementary material for: Synthesis of novel cytotoxic tetracyclic acridone derivatives and study of their molecular docking, ADMET, QSAR, bioactivity and protein binding properties
Source: Sci Rep. 2020 Nov 26;10:20720. doi: 10.1038/s41598-020-77590-1 (PMC7691360; doi:10.1038/s41598-020-77590-1)
Supplement: Supplementary file 1 — Supplementary Information. [file 41598_2020_77590_MOESM1_ESM.pdf]

Supplementary information

Synthesis of novel cytotoxic tetracyclic acridone derivatives and study of their molecular docking, ADMET, QSAR, bioactivity and protein binding properties

Rajkumar Veligeti[1,2], Rajesh Bagepalli Madhu[2,3,*], Jayashree Anireddy[1,*], Visweswara Rao Pasupuleti[4,*], Vijaya Kumar Reddy Avula[5,*], Krishna S Ethiraj[2], Srinivas Uppalanchi[2], Sivaprasad Kasturi[1,2], Yogeeswari Perumal[6], Hasitha Shilpa Anantaraju[6], Naveen Polkam[1], Mallilkarjuna Reddy Guda[5], Swetha Vallela[5], Grigory Vasilievich Zyryanov[5,7]

[1]Centre for Chemical Sciences and Technology, Institute of Science & Technology, Jawaharlal Nehru Technological University Hyderabad, Hyderabad-500085, Telangana, India

[2]Medicinal Chemistry Division, GVK Biosciences Private Limited, Plot No. 28A, IDA Nacharam, Hyderabad-500076, Telangana, India

[3]Discovery & Development Solutions, GVK Biosciences Private Limited, Plot No.284A, Jigini Village, Bengaluru-562106, Karnataka, India

[4]Department of Biomedical Sciences and Therapeutics, Faculty of Medicine and Health Sciences, Universiti Malaysia Sabah, Kota Kinabalu, 88400, Sabah, Malaysia

[5]Chemical Engineering Institute, Ural Federal University, Yekaterinburg 620002, Russian Federation

[6]Drug Discovery Research Laboratory, Department of Pharmacy, Birla Institute of Technology & Science - Pilani, Hyderabad Campus, Hyderabad - 500078, Telangana, India

[7]Ural Division of the Russian Academy of Sciences, I. Ya. Postovskiy Institute of Organic Synthesis, 22 S. Kovalevskoy Street, Yekaterinburg 620219, Russian Federation

*Corresponding author: jayashreeanireddy@gmail.com (J. Anireddy); rajeshbagepallimadhu@gmail.com (B.M.Rajesh); pvrao@ums.edu.my (V.R. Pasupuleti); vijaykumarreddy.jntuh@gmail.com (V.K.R. Avula).

Analysis of Compound 2:

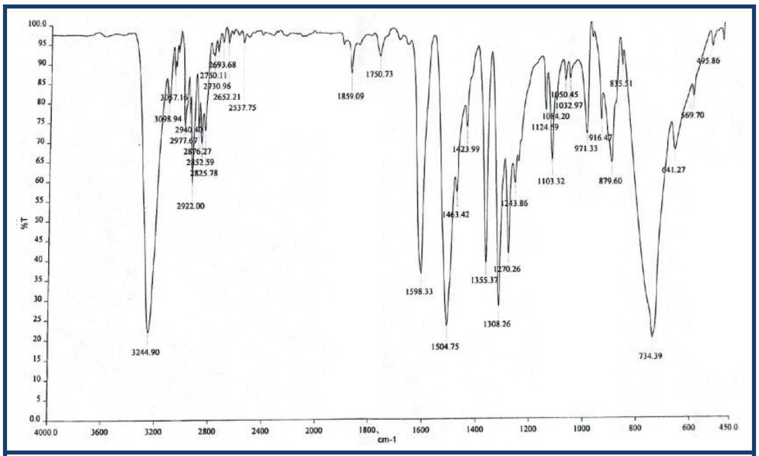

FTIR spectrum of Compound 2

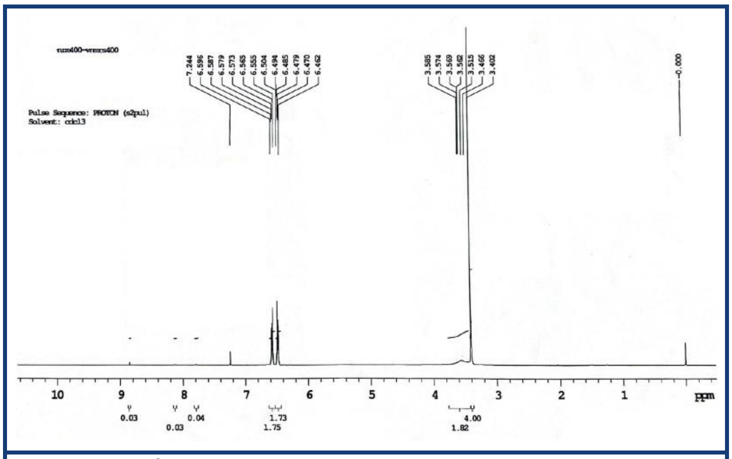

¹H NMR spectrum of Compound 2

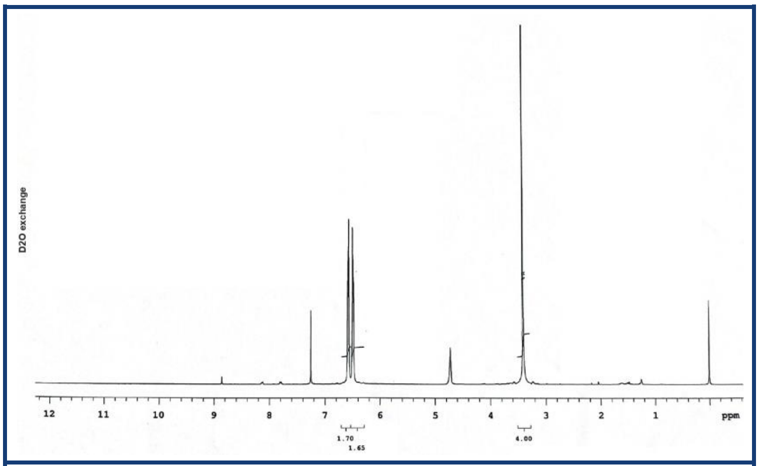

¹H NMR D2O Exchange spectrum of Compound 2

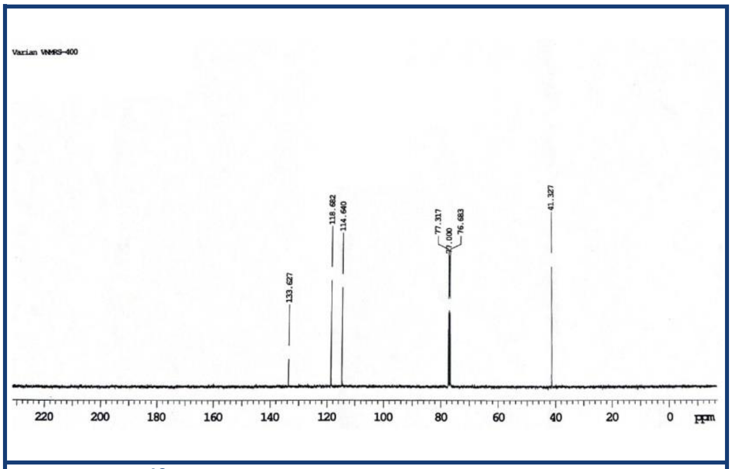

¹³C NMR spectrum of Compound 2

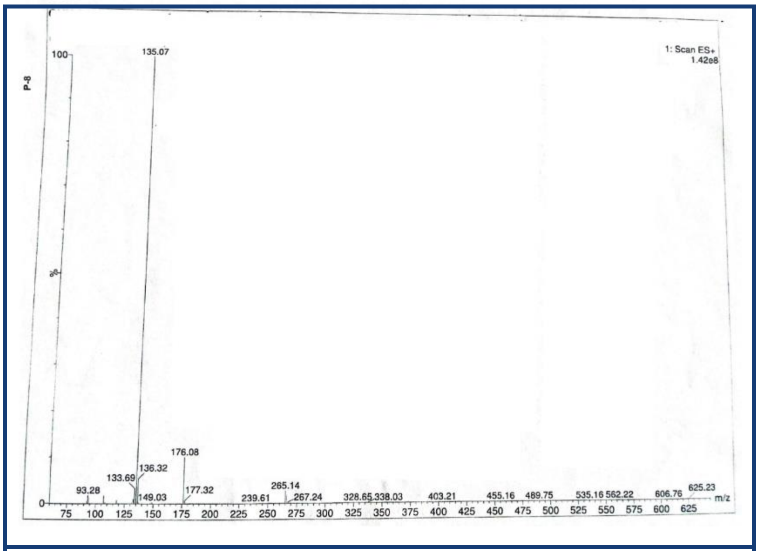

Mass spectrum of Compound 2

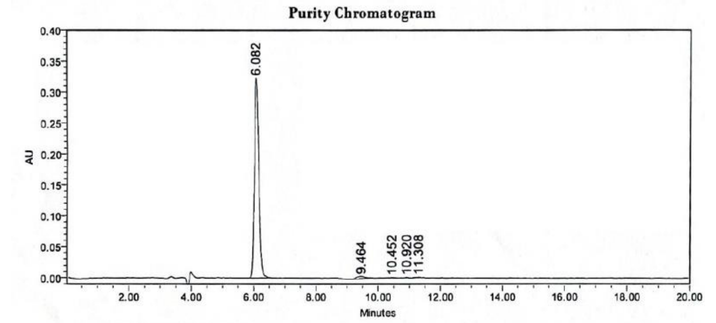

	RT	Area	% Area
1	6.08	3169731	97.34
2	9.46	61872	1.90
3	10.45	5718	0.18
4	10.92	6700	0.21
5	11.31	12292	0.38

Column : XBridge C18 (4.6X250mm)5u
Mobile Phase: : A:ACN B:0.1% TFA
T/%B : 0/90,4/90,8/10,18/10,20/90,20.1/90.
Flow Rate: : 1.0 ml/min
Temp: : Ambient
Diluent: : Mobile phase

HPLC Chromatogram of Compound 2

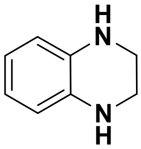

Structure of Compound 2

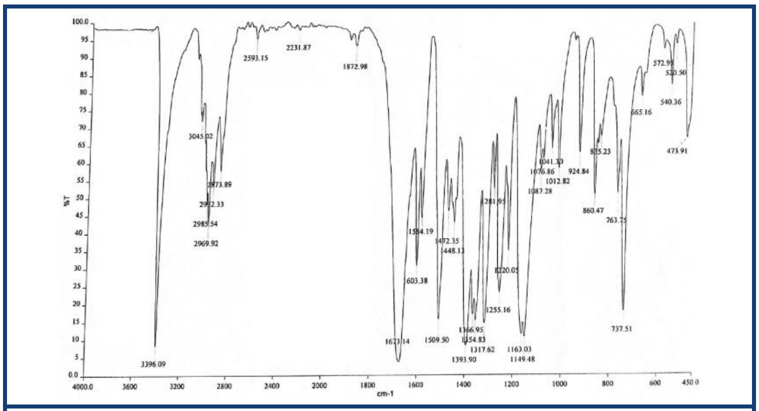

FTIR spectrum of Compound 3

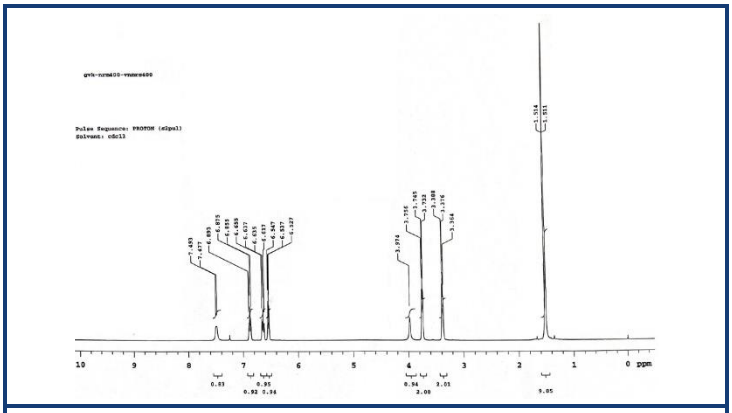

^{1}H NMR spectrum of Compound 3

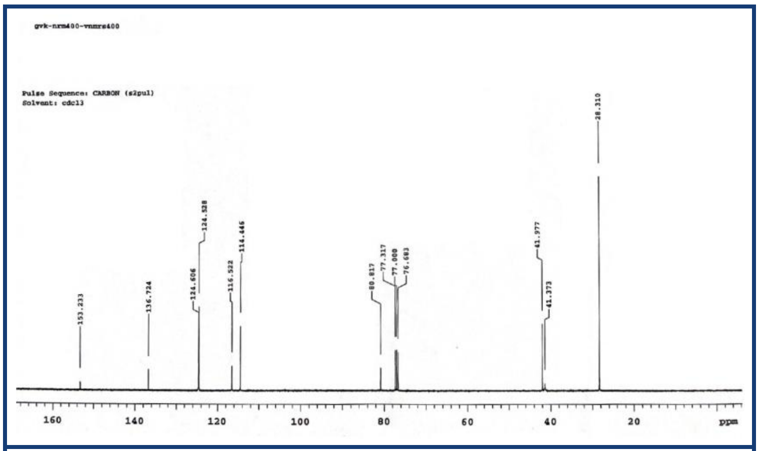

^{13}C NMR spectrum of Compound 3

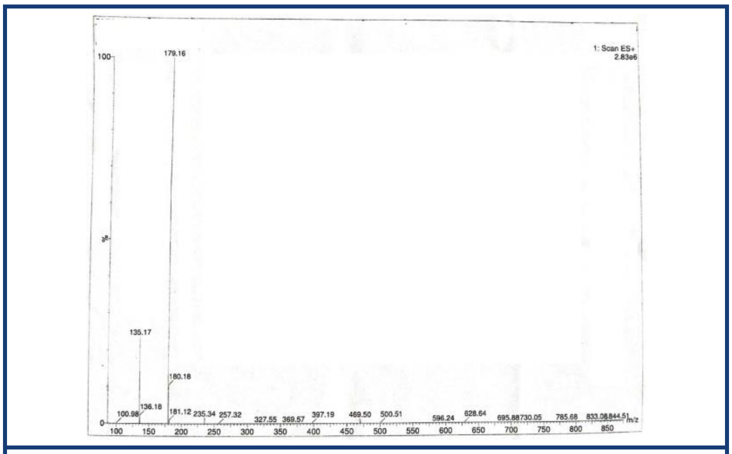

Mass spectrum of Compound 3

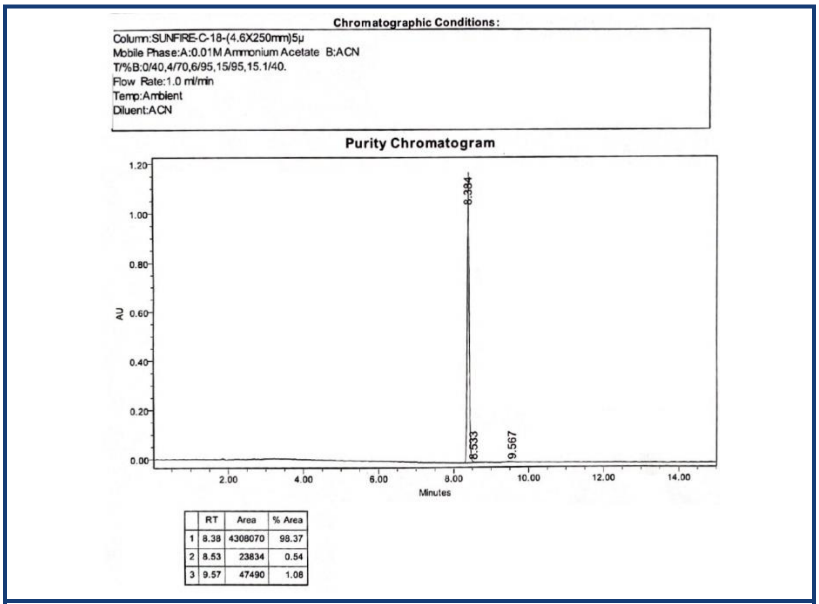

	RT	Area	% Area
1	8.38	4308070	98.37
2	8.53	23834	0.54
3	9.57	47490	1.08

HPLC Chromatogram of Compound 3

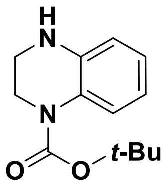

Structure of Compound 3

Analysis of Compound 4:

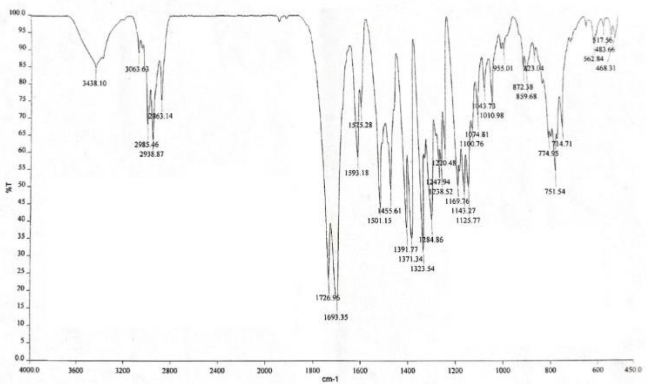

FTIR spectrum of Compound 4

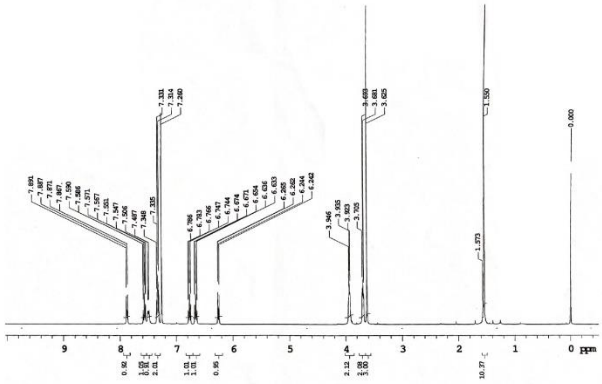

^{1}H NMR spectrum of Compound 4

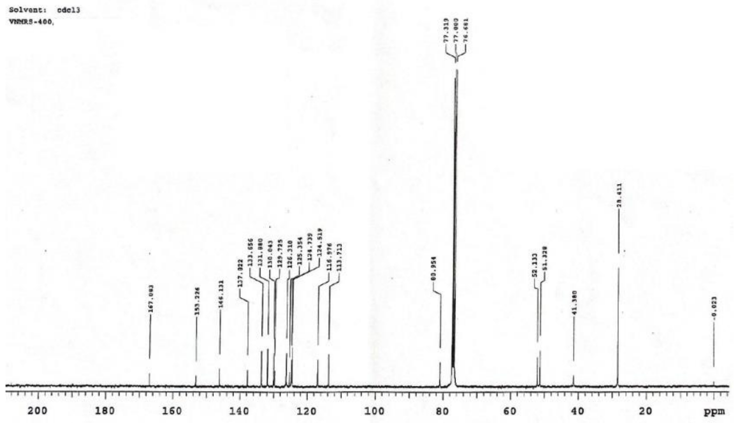

^{13}C NMR spectrum of Compound 4

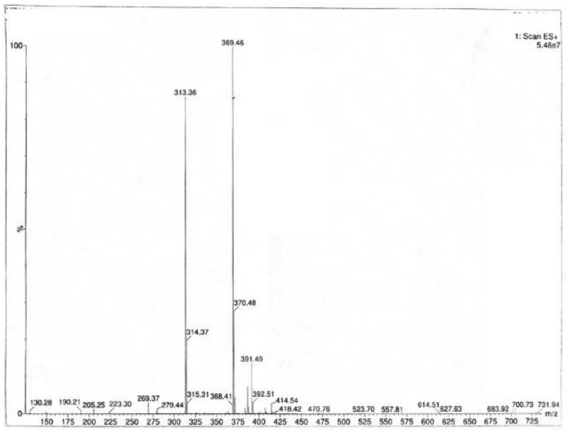

Mass spectrum of Compound 4

Purity Chromatogram

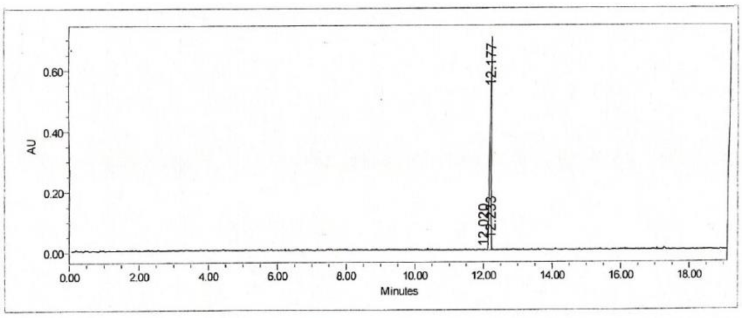

Peak Results

	RT	Area	% Area
1	12.020	4728	0.22
2	12.177	2146770	98.95
3	12.233	18017	0.83

HPLC Method Conditions:

Column: XBridge C18(150 X4.6mm, 3.5μm)
Mobile Phase: A:10mM Ammonium Bicarbonate, B:100% ACN
Gradient: Time/ %B: 0/5, 1.5/5, 3/15, 7/55, 10/95, 14/95, 17/5, 20/5
Column Temp:35°C, Flow Rate:1.0ml/min
Diluent:70:30(ACN:H2O)

HPLC Chromatogram of Compound 4

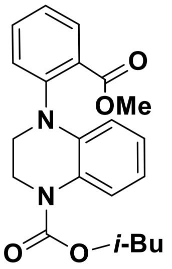

Structure of Compound 4

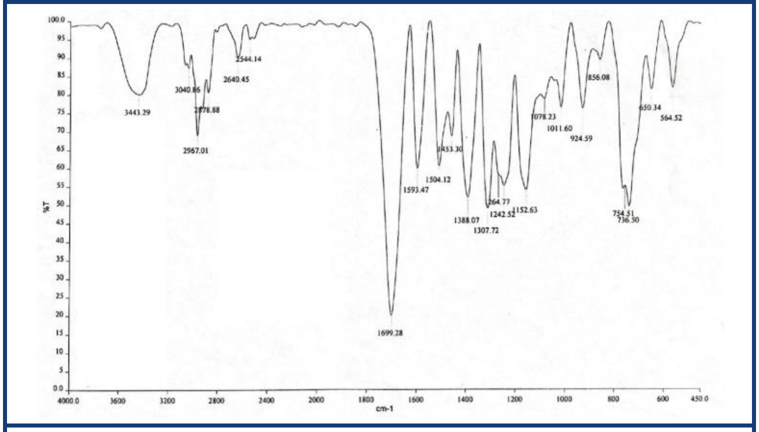

FTIR spectrum of Compound 5

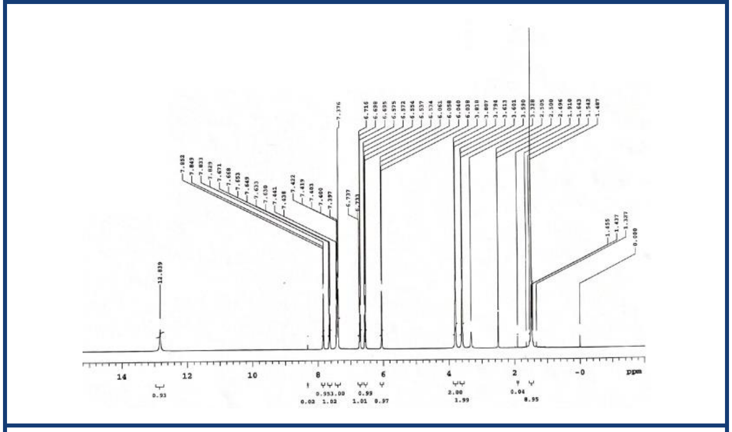

^{1}H NMR spectrum of Compound 5

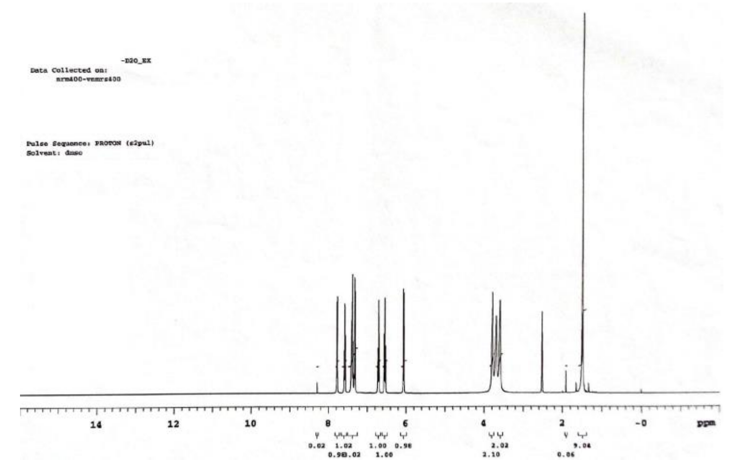

^{1}H NMR D2O Exchange spectrum of Compound 5

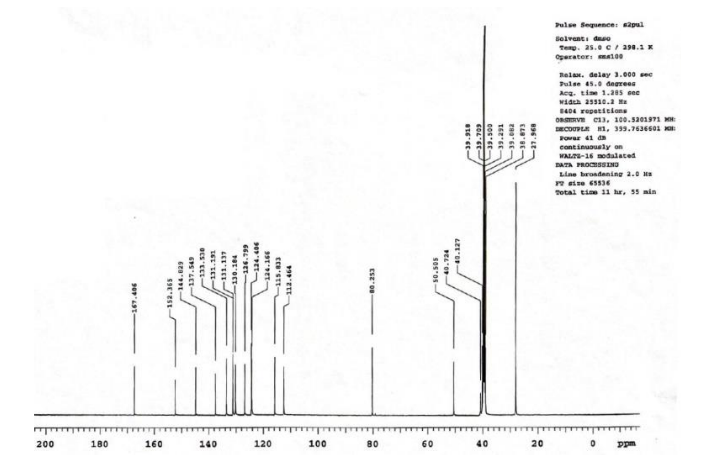

^{13}C NMR spectrum of Compound 5

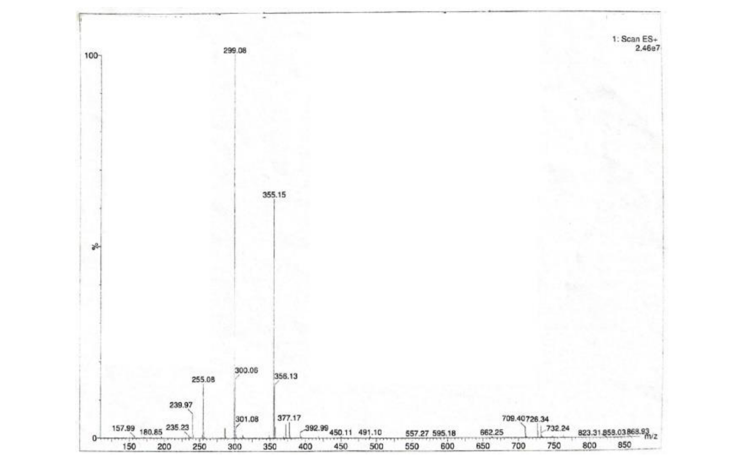

Mass spectrum of Compound 5

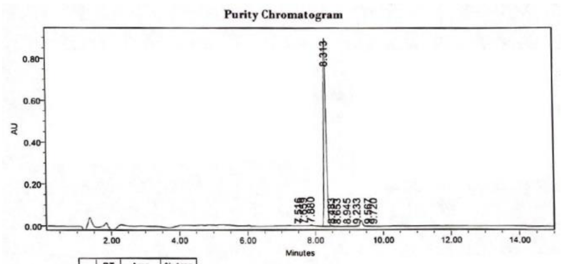

	RT	Area	% Area
1	7.52	3713	0.08
2	7.66	3666	0.08
3	7.88	22883	0.52
4	8.31	4372789	98.79
5	8.48	6146	0.14
6	8.65	4434	0.10
7	8.94	3480	0.08
8	9.23	1801	0.04
9	9.57	5520	0.12
10	9.72	1995	0.05

Column : X Bridge C18(4.6X150mm)3.5 um
Mobile Phase: : A:ACN B:0.1% HCOOH
T/%A : 0/10,1/10,3/45,4/45,6/90,15/90,15.1/10.
Flow Rate: : 1.0 ml/min
Temp: : Ambient
Diluent: : Mobile phase

HPLC Chromatogram of Compound 5

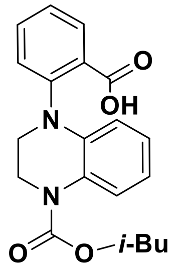

Structure of Compound 5

Analysis of Compound 6:

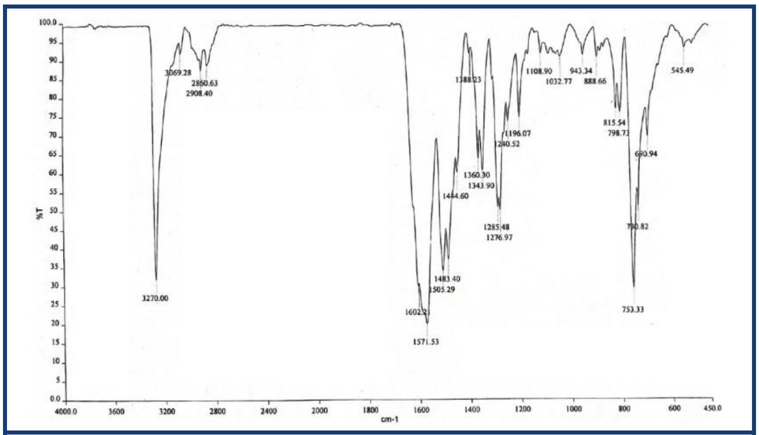

FTIR spectrum of Compound 6

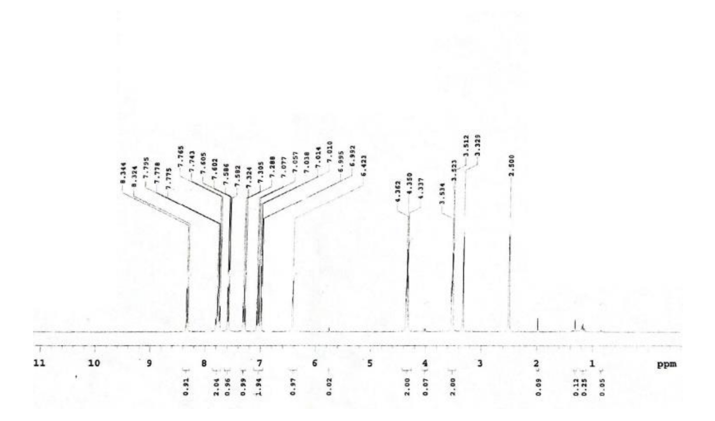

^{1}H NMR spectrum of Compound 6

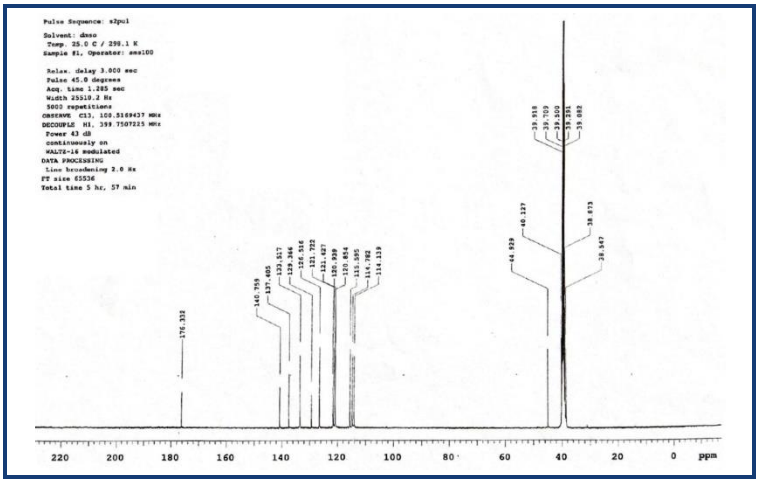

^{13}C NMR spectrum of Compound 6

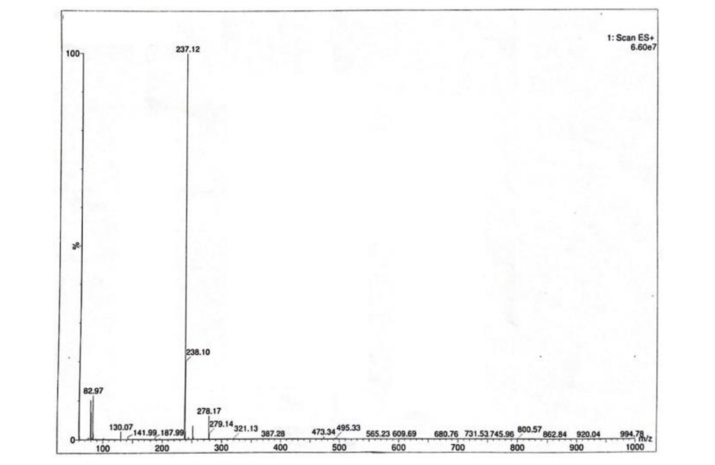

Mass spectrum of Compound 6

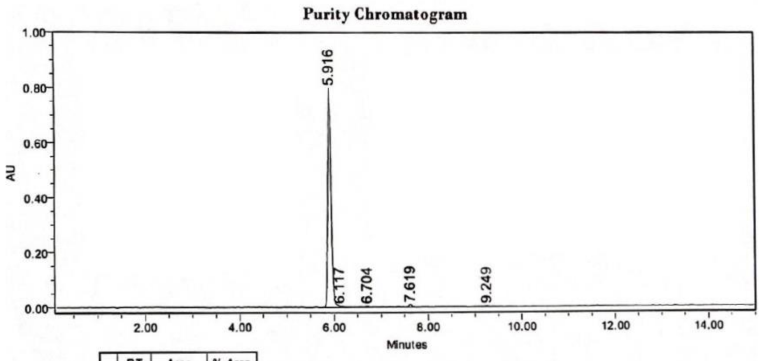

	RT	Area	% Area
1	5.92	3883449	97.54
2	6.12	32964	0.83
3	6.70	19440	0.49
4	7.62	32667	0.82
5	9.25	12996	0.33

Column : X Bridge C18(4.6X150mm)3.5 um
Mobile Phase: : A:ACN B:0.1% HCOOH
T/%A : 0/10,1/10,3/45,4/45,6/90,15/90,15.1/10.
Flow Rate: : 1.0 ml/min
Temp: : Ambient
Diluent: : Mobile phase

HPLC Chromatogram of Compound 6

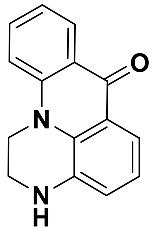

Structure of Compound 6

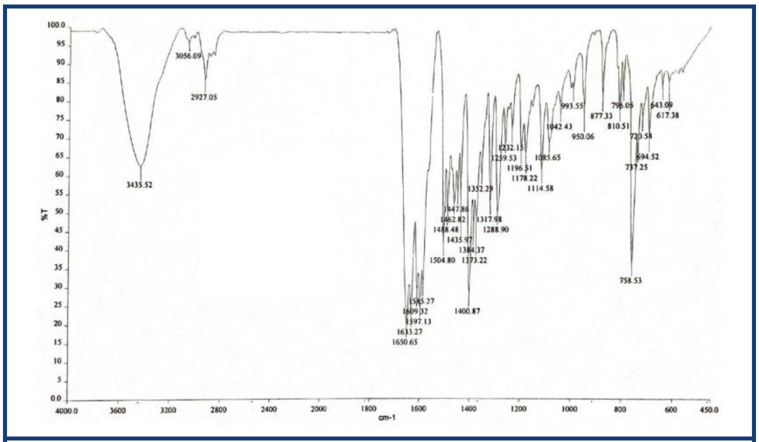

FTIR spectrum of Compound 7a

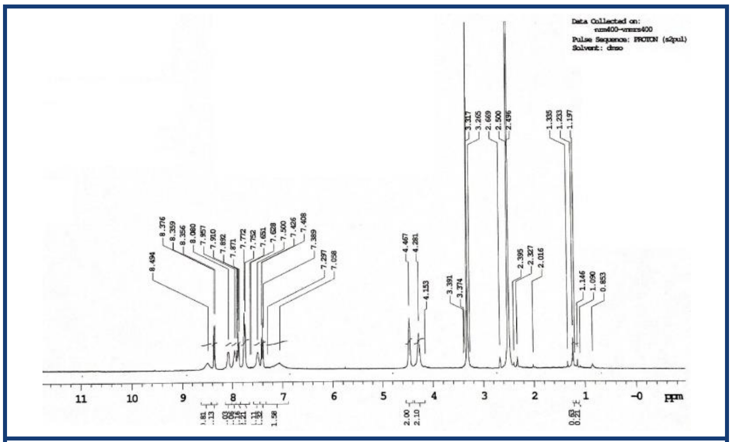

^{1}H NMR spectrum of Compound 7a

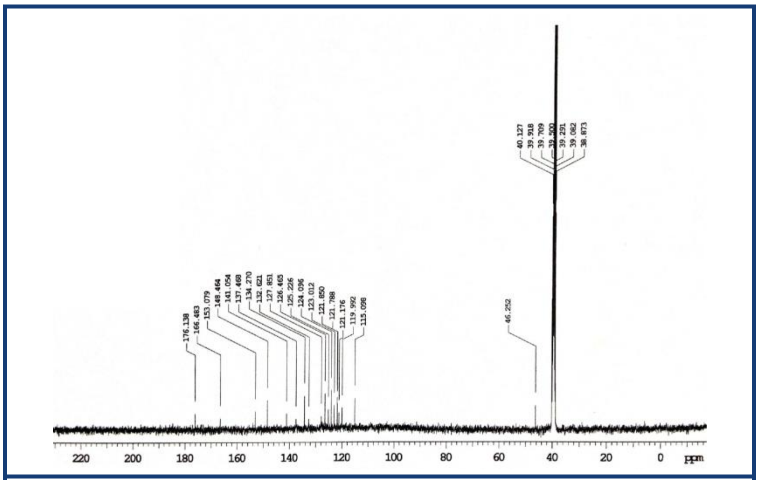

^{13}C NMR spectrum of Compound 7a

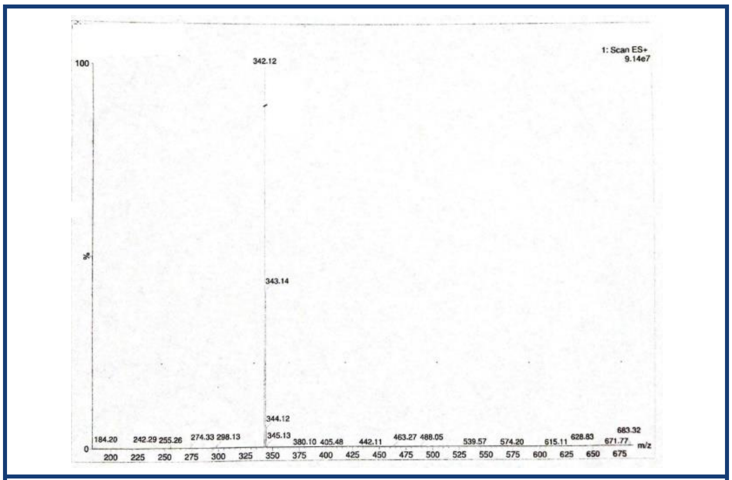

Mass spectrum of Compound 7a

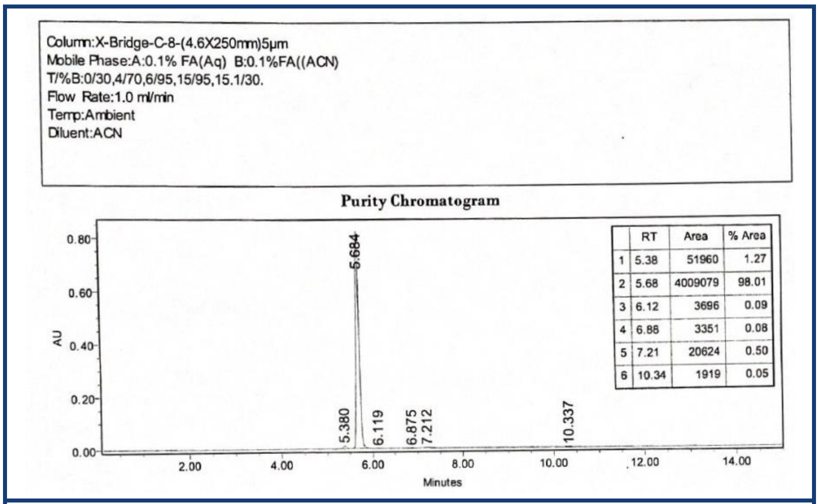

HPLC Chromatogram of Compound 7a

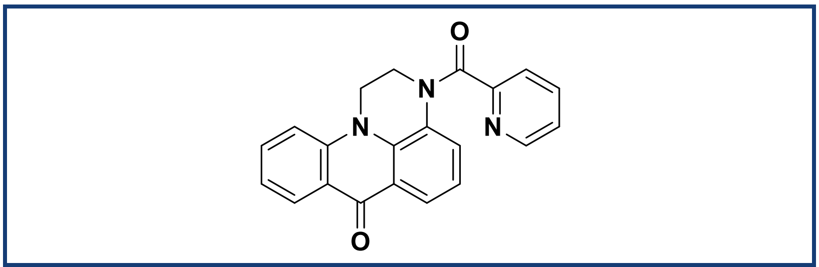

Structure of Compound 7a

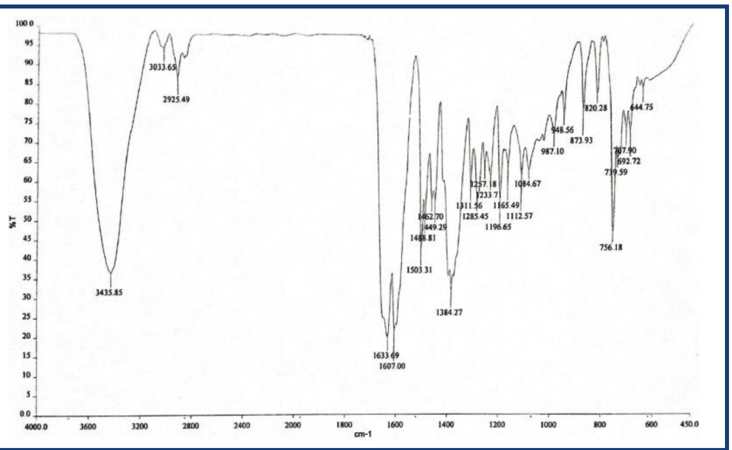

FTIR spectrum of Compound 7b

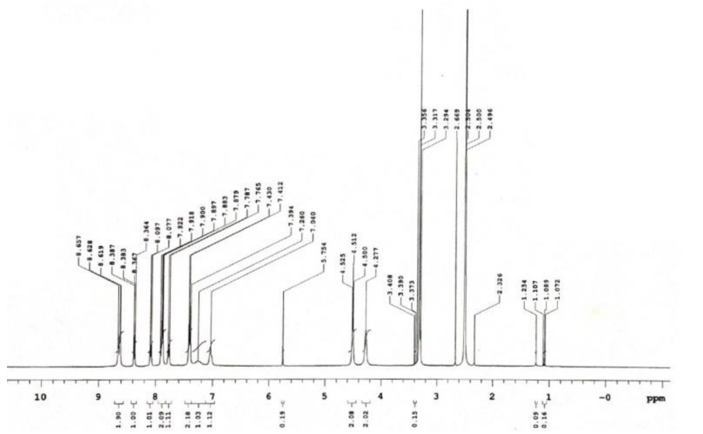

^{1}H NMR spectrum of Compound 7b

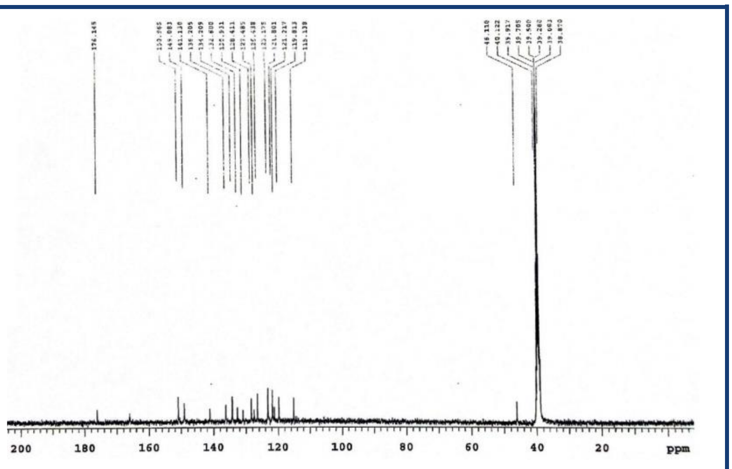

^{13}C NMR spectrum of Compound 7b

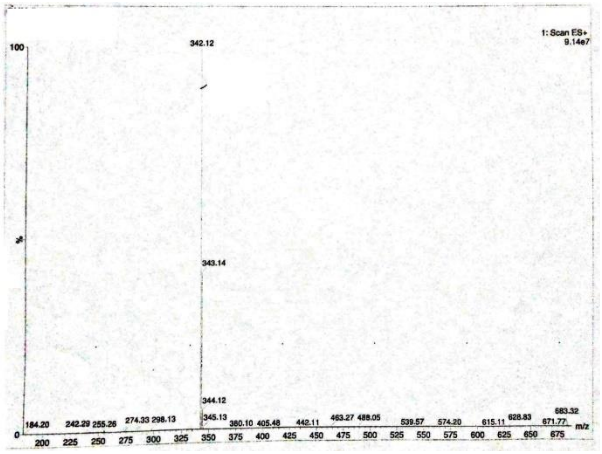

Mass spectrum of Compound 7b

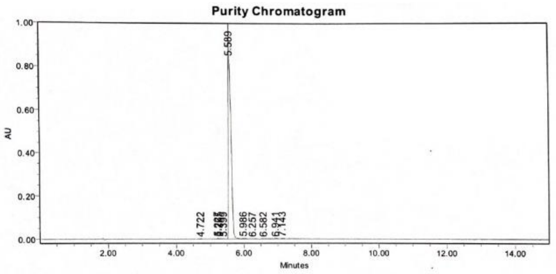

	RT	Area	% Area
1	4.72	6768	0.13
2	5.23	7065	0.14
3	5.27	5349	0.10
4	5.40	1877	0.04
5	5.59	5085863	98.78
6	5.99	10399	0.20
7	6.26	3643	0.07
8	6.58	6958	0.14
9	6.94	11688	0.23

	RT	Area	% Area
10	7.14	9051	0.18

Chromatographic Conditions:

Column: X-BRIDGE C18 (4.6X150mm)3.5μ
Mobile Phase:A:0.1%FA(Aq) B:0.1%FA(ACN)
T/%B:0/10,1/10,3/45,4/45,6/90,15/90,15.1/10
Flow Rate:1.0 ml/min
Temp:Ambient
Diluent:ACN+H20

HPLC Chromatogram of Compound 7b

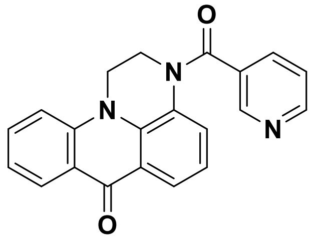

Structure of Compound 7b

Analysis of Compound 7c:

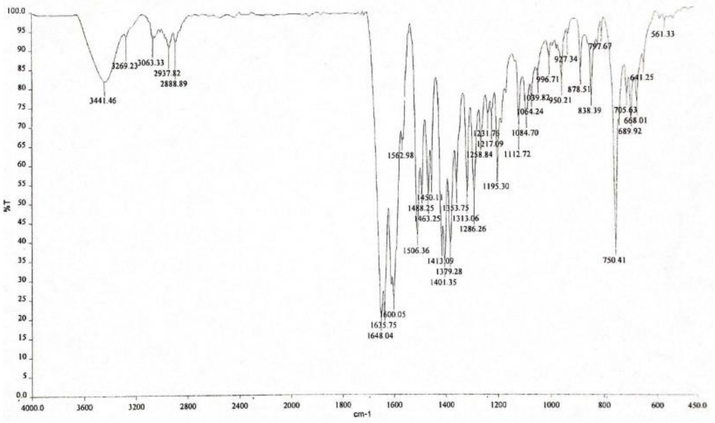

FTIR spectrum of Compound 7c

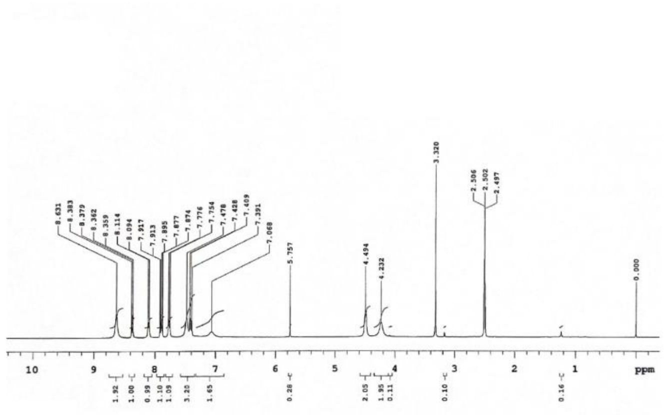

^{1}H NMR spectrum of Compound 7c

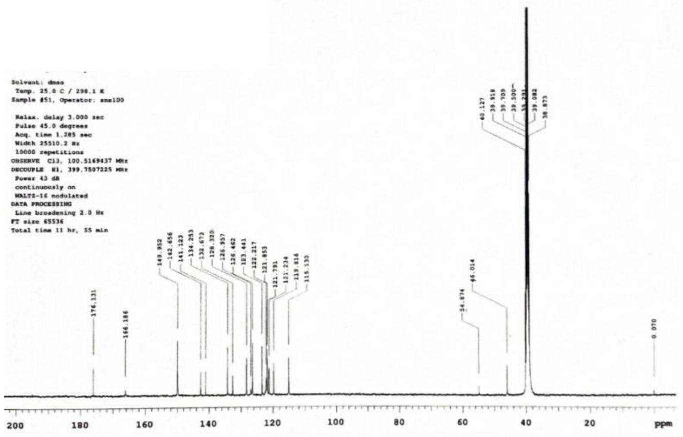

^{13}C NMR spectrum of Compound 7c

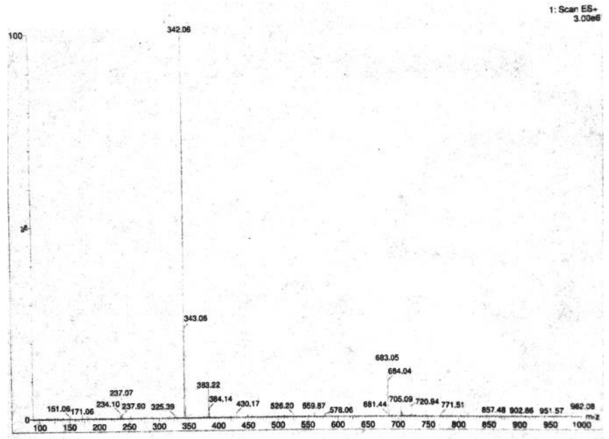

Mass spectrum of Compound 7c

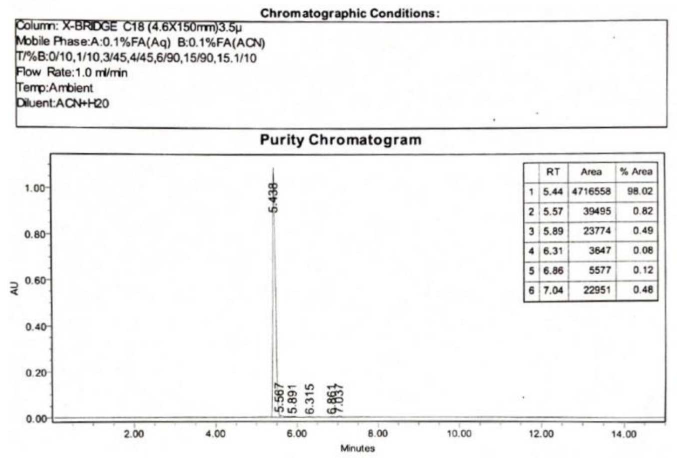

HPLC Chromatogram of Compound 7c

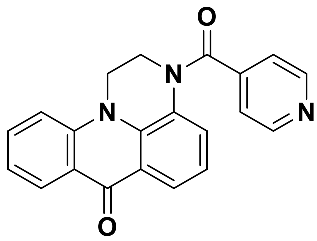

Structure of Compound 7c

Analysis of Compound 7d:

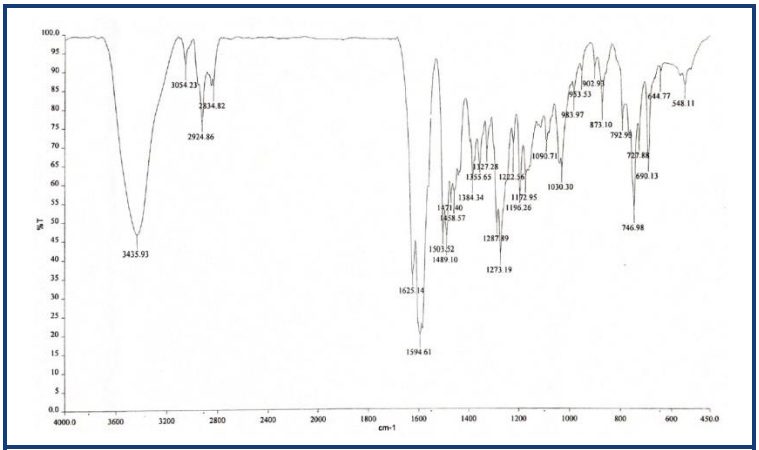

FTIR spectrum of Compound 7d

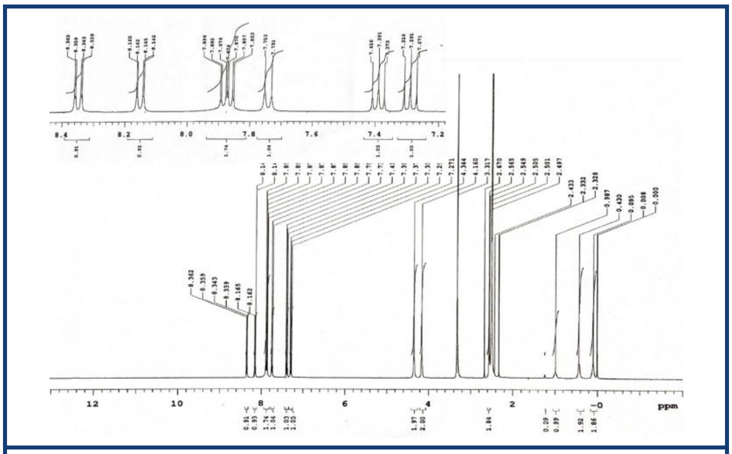

^{1}H NMR spectrum of Compound 7d

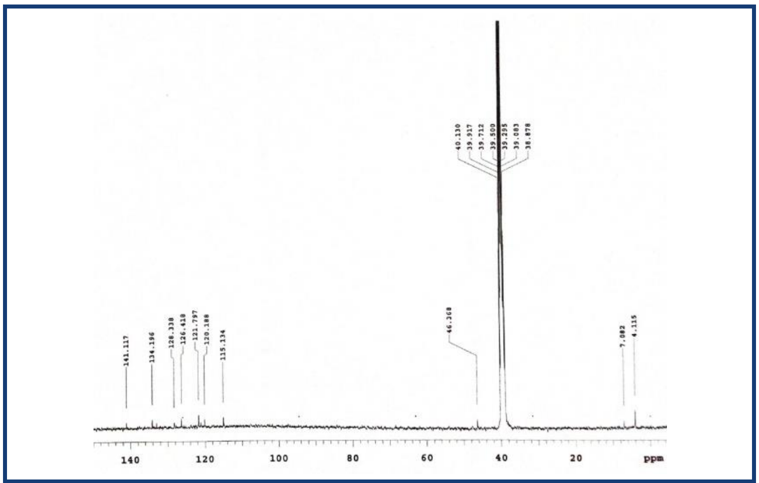

^{13}C NMR spectrum of Compound 7d

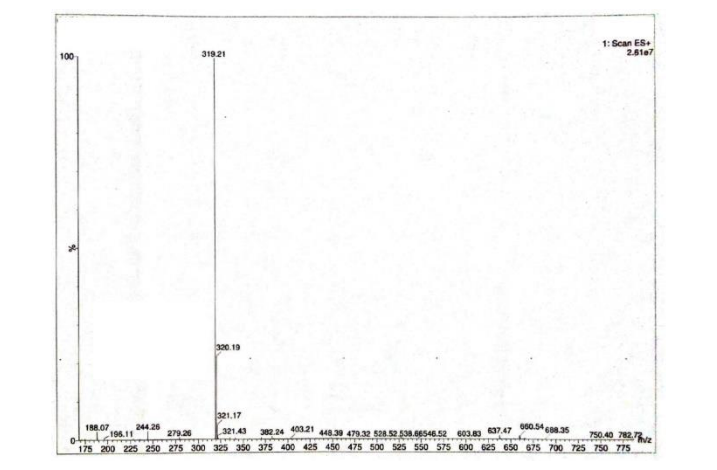

Mass spectrum of Compound 7d

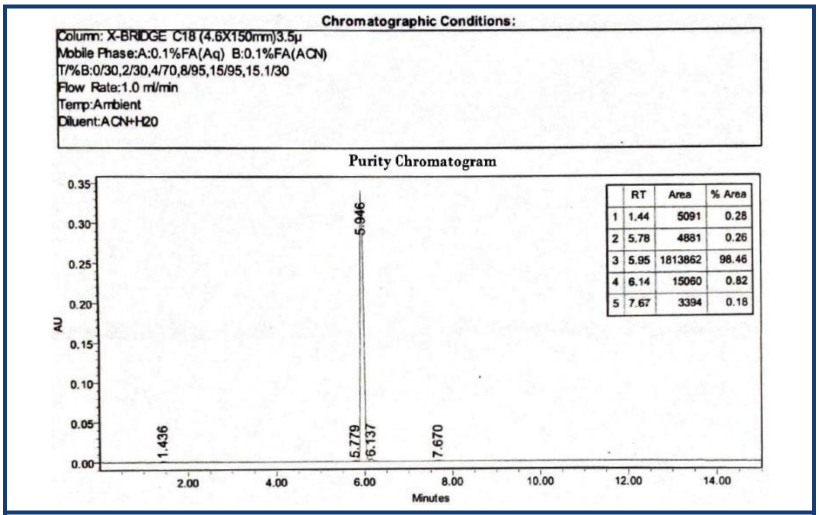

	RT	Area	% Area
1	1.44	5091	0.28
2	5.78	4881	0.26
3	5.95	1813862	98.46
4	6.14	15060	0.82
5	7.67	3394	0.18

HPLC Chromatogram of Compound 7d

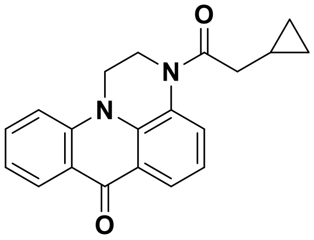

Structure of Compound 7d

Analysis of Compound 7e:

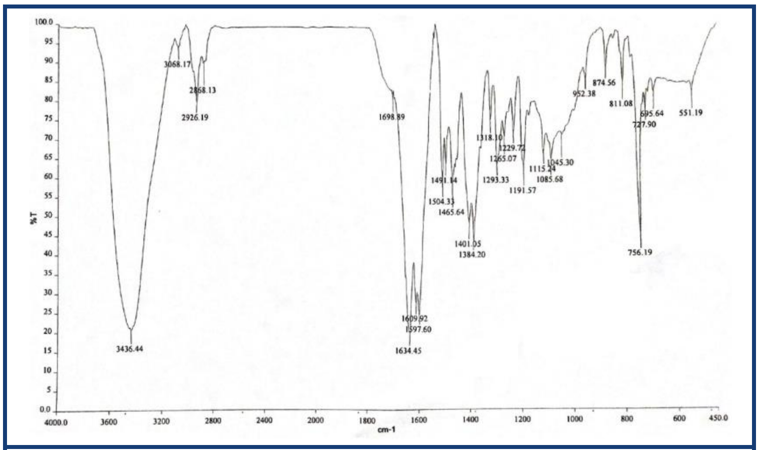

FTIR spectrum of Compound 7e

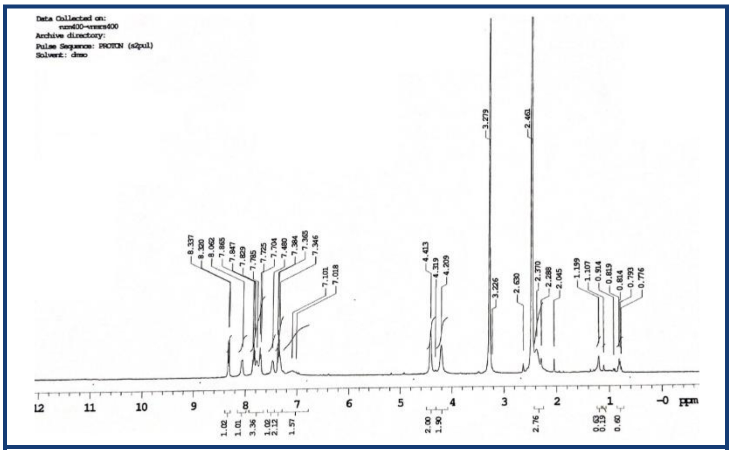

^{1}H NMR spectrum of Compound 7e

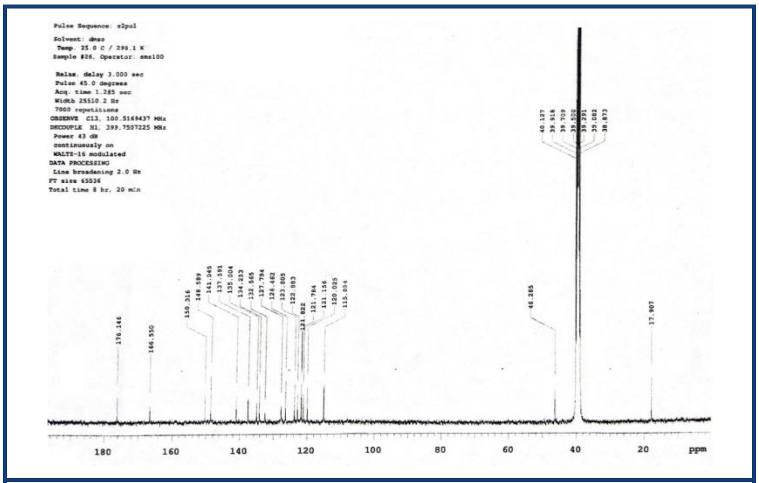

^{13}C NMR spectrum of Compound 7e

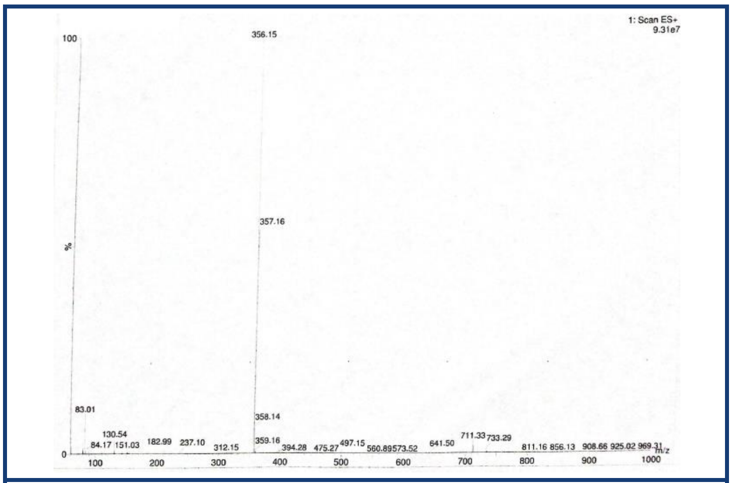

Mass spectrum of Compound 7e

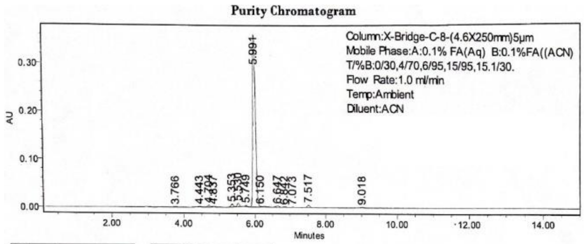

	RT	Area	% Area
1	3.77	4110	0.21
2	4.44	4920	0.25
3	4.70	21350	1.07
4	4.84	9095	0.46
5	5.35	29214	1.47
6	5.53	32123	1.61
7	5.75	11448	0.58
8	5.99	1810362	91.00
9	6.15	15396	0.77
10	6.65	15391	0.77
11	6.84	4385	0.22
12	7.07	1846	0.09
13	7.52	23011	1.16
14	9.02	6792	0.34

HPLC Chromatogram of Compound 7e

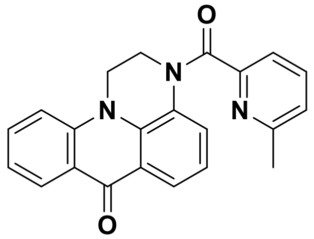

Structure of Compound 7e

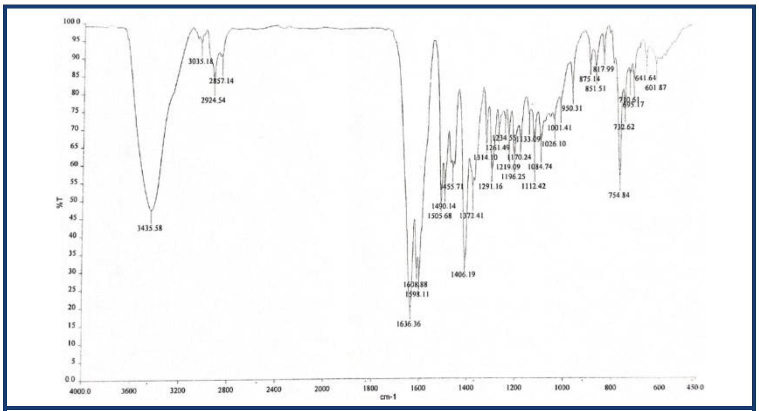

FTIR spectrum of Compound 7f

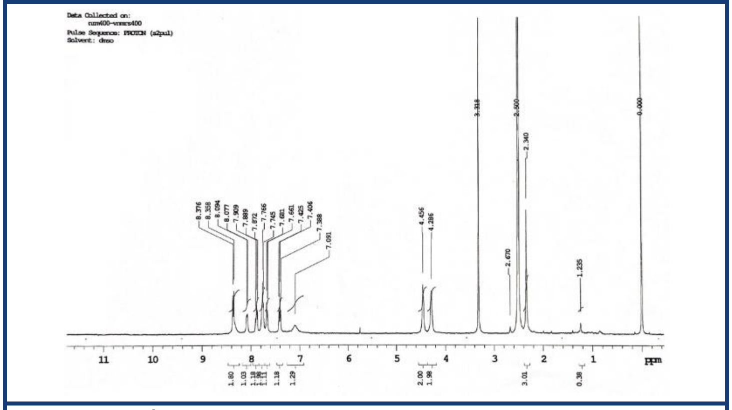

^{1}H NMR spectrum of Compound 7f

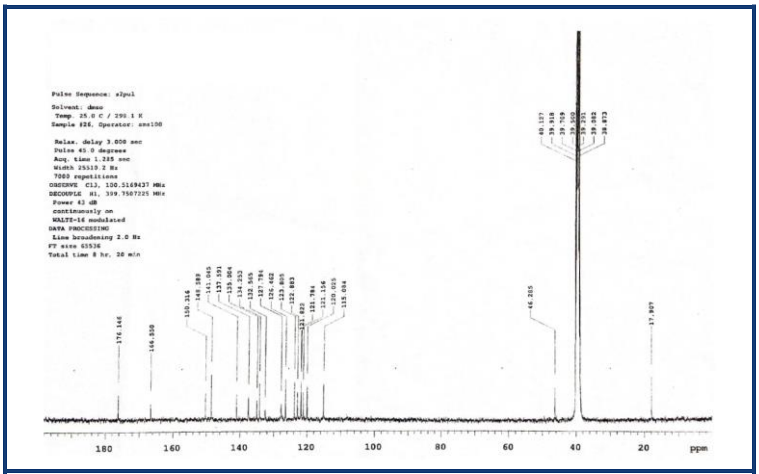

^{13}C NMR spectrum of Compound 7f

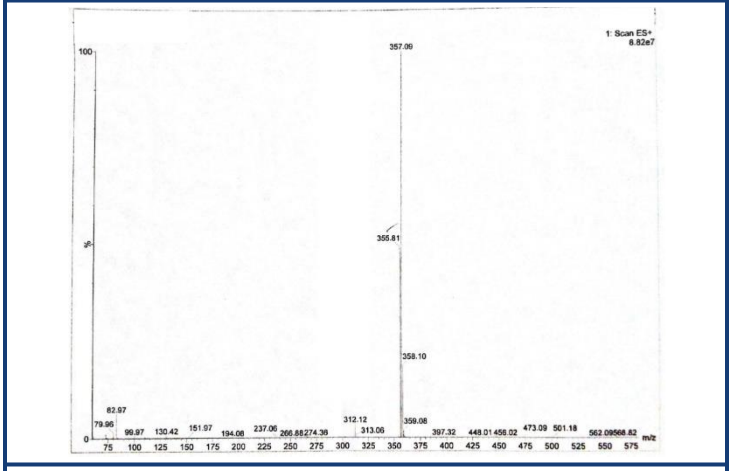

Mass spectrum of Compound 7f

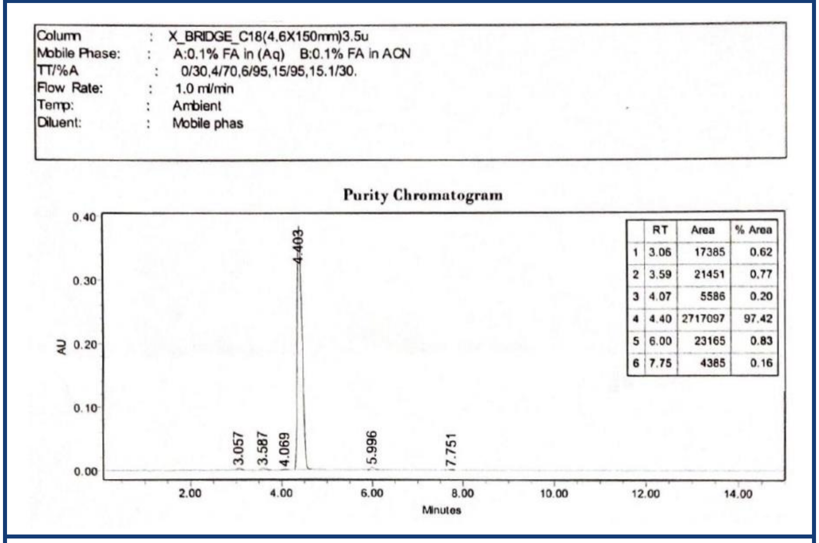

HPLC Chromatogram of Compound 7f

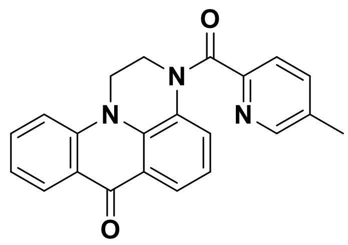

Structure of Compound 7f

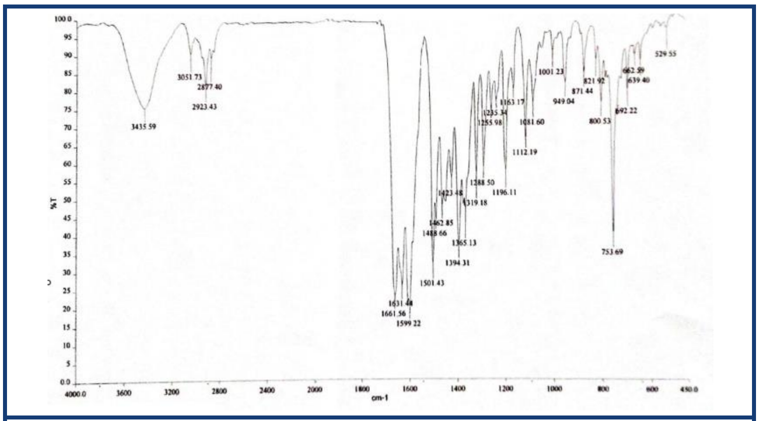

FTIR spectrum of Compound 7g

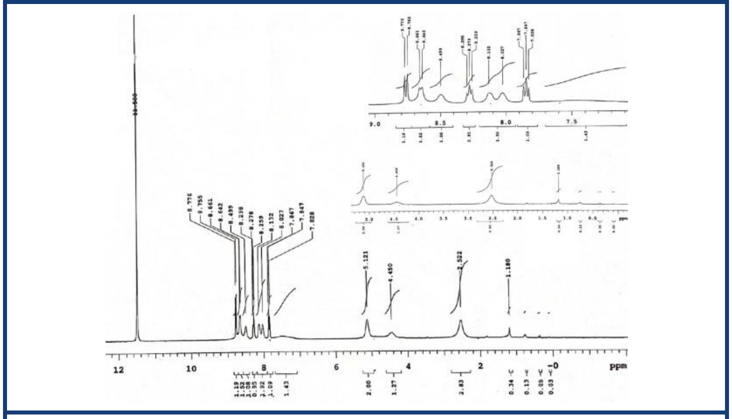

^{1}H NMR spectrum of Compound 7g

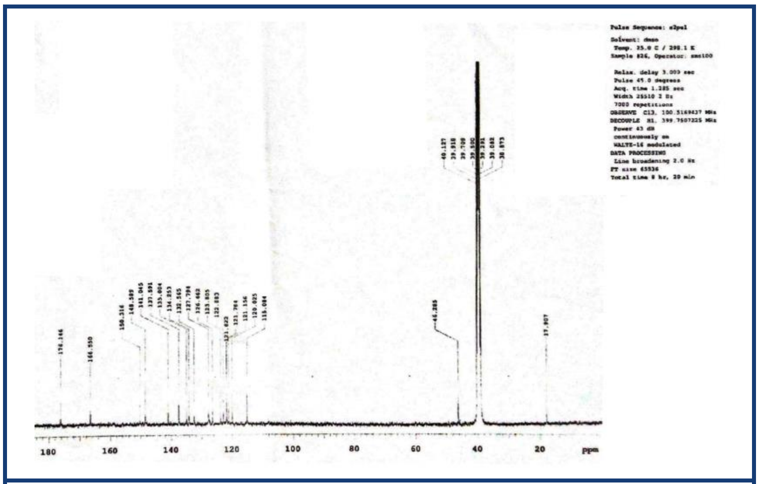

^{13}C NMR spectrum of Compound 7g

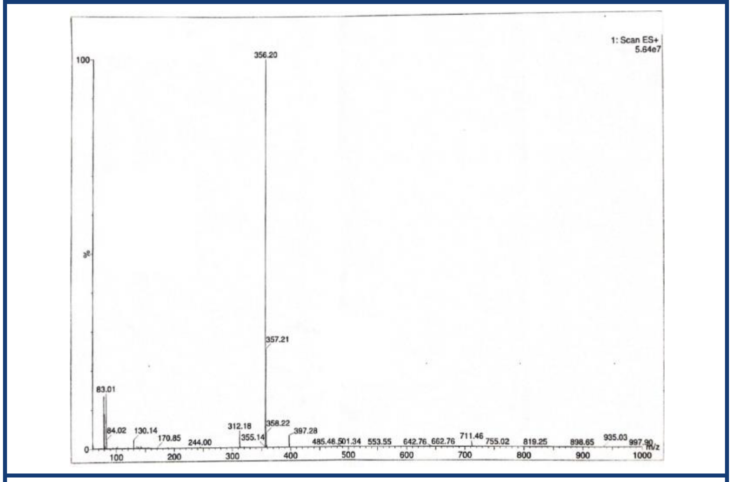

Mass spectrum of Compound 7g

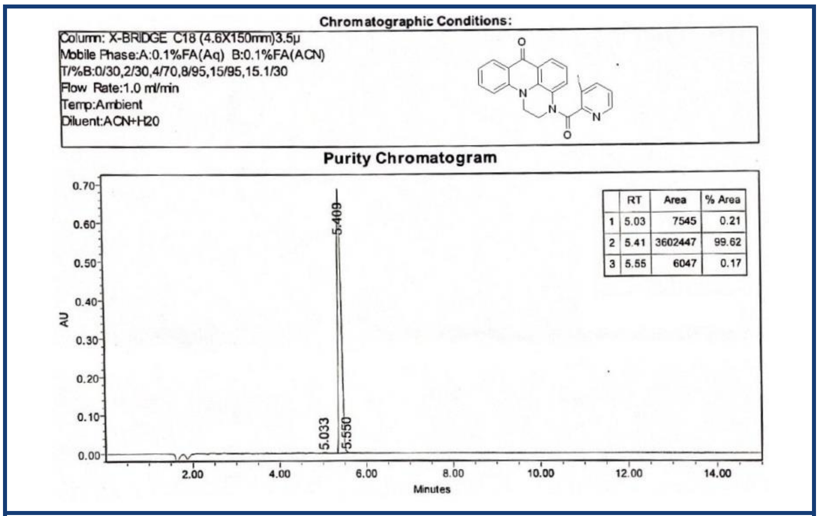

	RT	Area	% Area
1	5.03	7545	0.21
2	5.41	3602447	99.62
3	5.55	6047	0.17

HPLC Chromatogram of Compound 7g

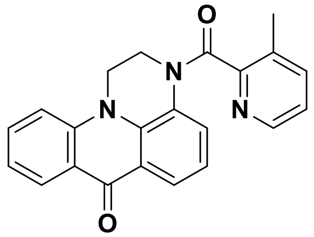

Structure of Compound 7g

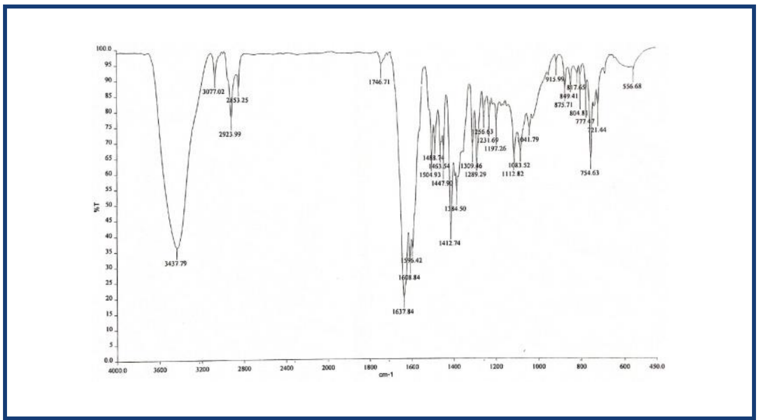

FTIR spectrum of Compound 7h

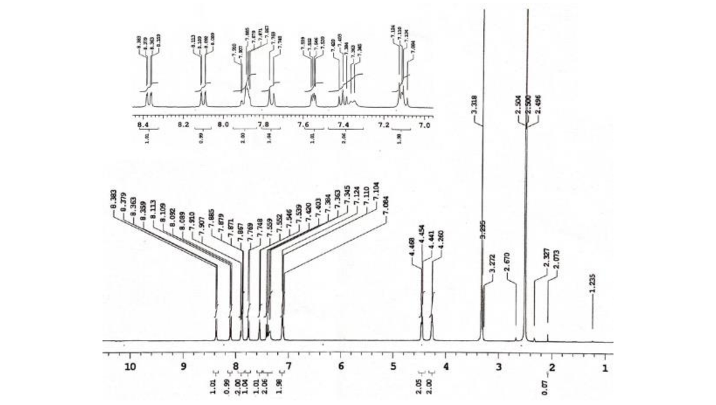

^{1}H NMR spectrum of Compound 7h

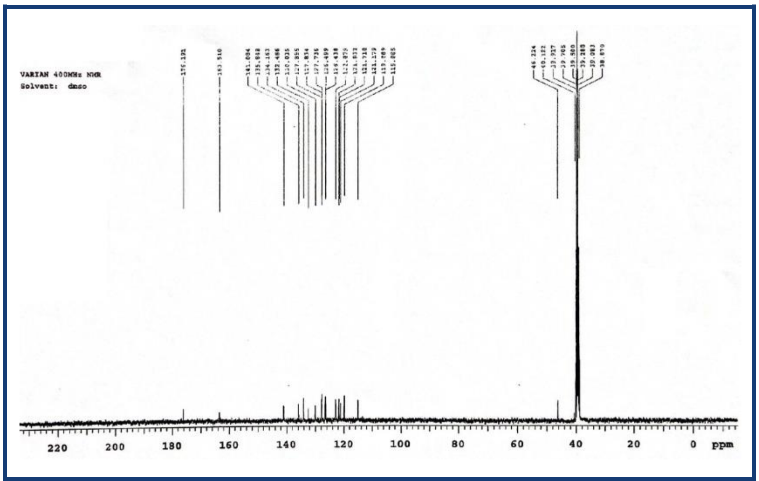

^{13}C NMR spectrum of Compound 7h

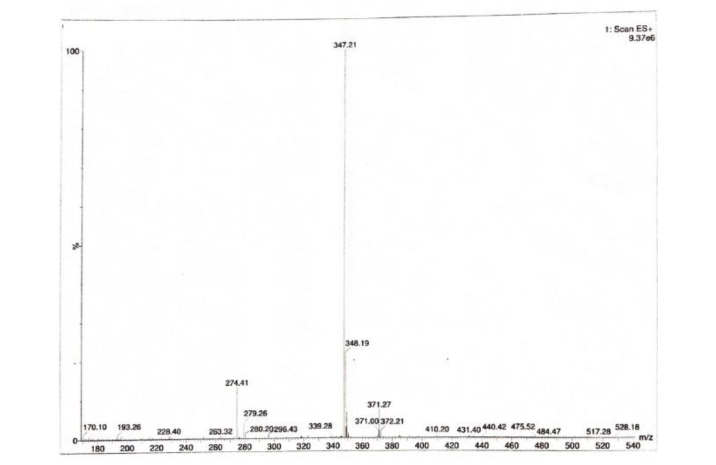

Mass spectrum of Compound 7h

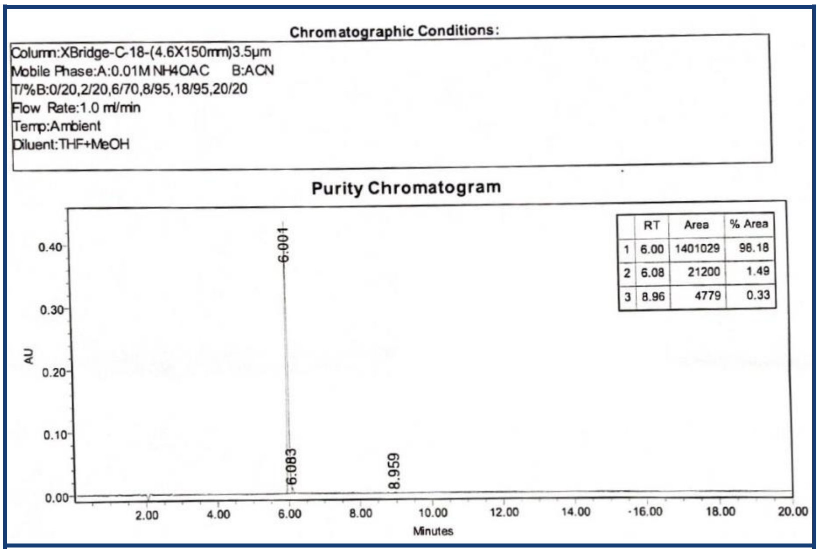

HPLC Chromatogram of Compound 7h

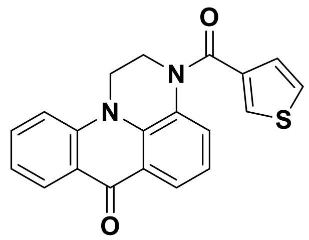

Structure of Compound 7h

Analysis of Compound 7i:

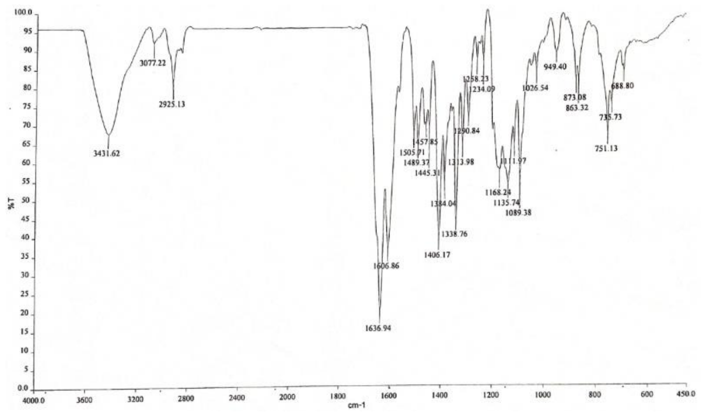

FTIR spectrum of Compound 7i

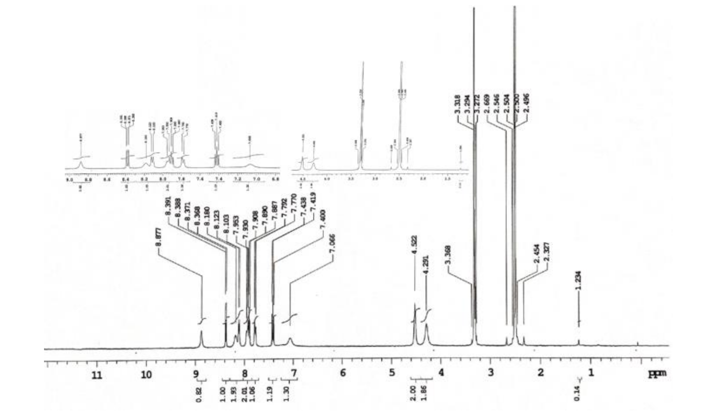

^{1}H NMR spectrum of Compound 7i

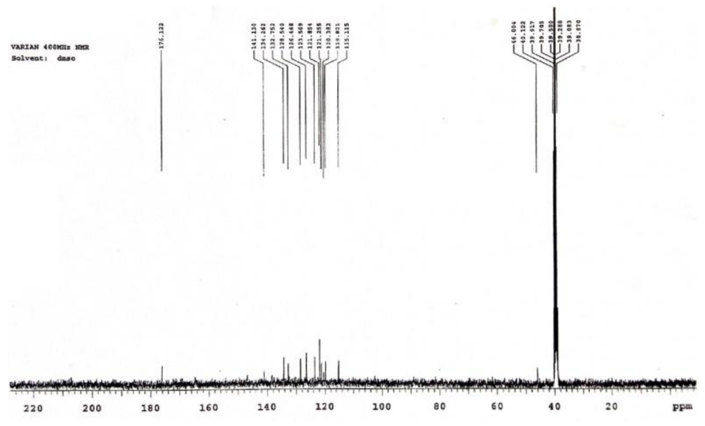

^{13}C NMR spectrum of Compound 7i

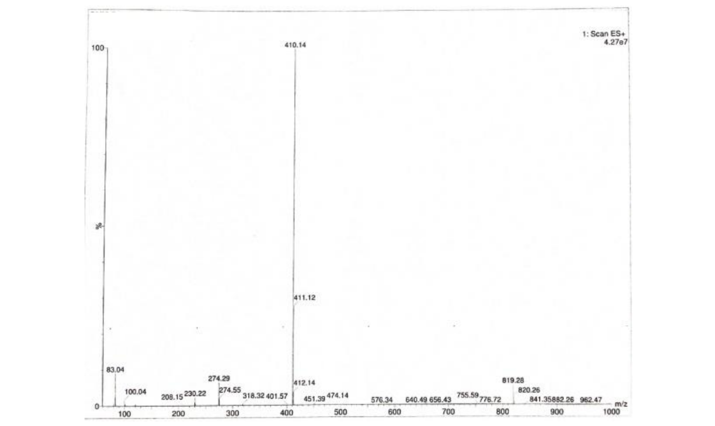

Mass spectrum of Compound 7i

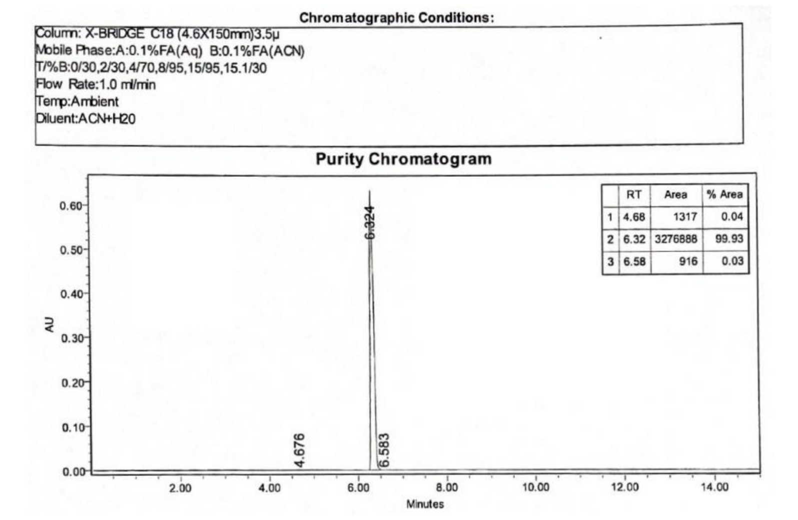

HPLC Chromatogram of Compound 7i

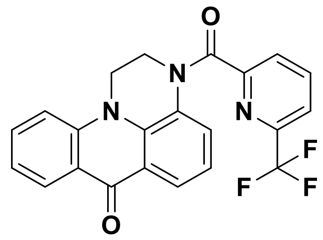

Structure of Compound 7i

Analysis of Compound 7j:

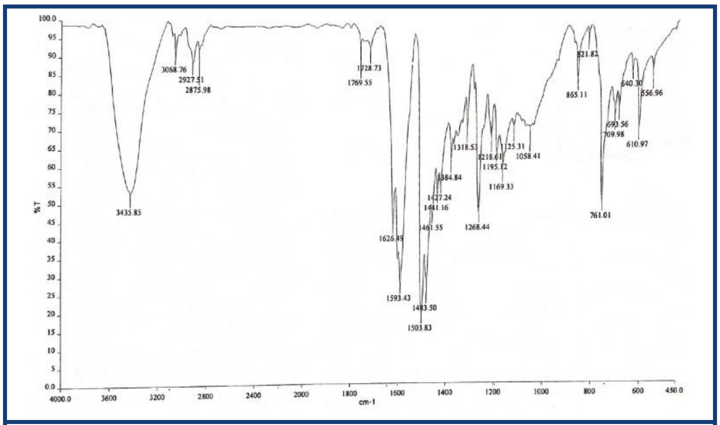

FTIR spectrum of Compound 7j

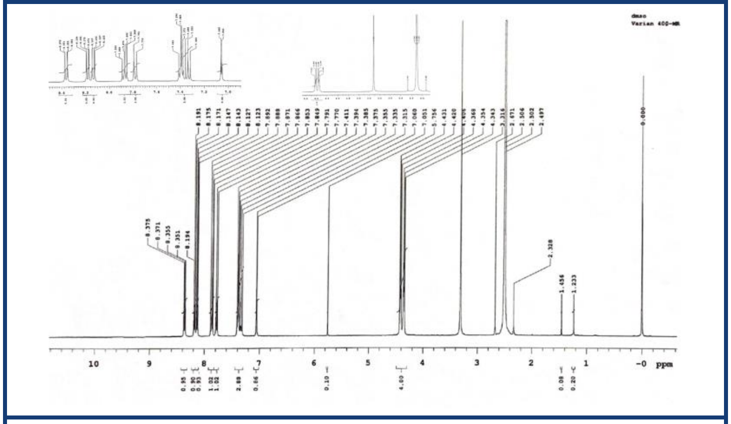

^{1}H NMR spectrum of Compound 7j

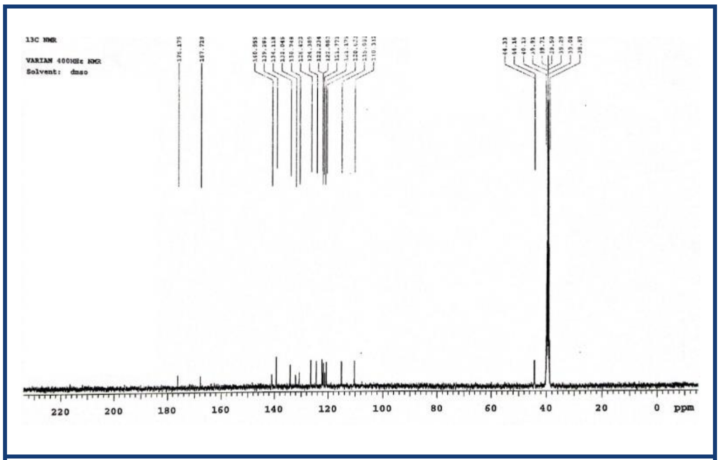

^{13}C NMR spectrum of Compound 7j

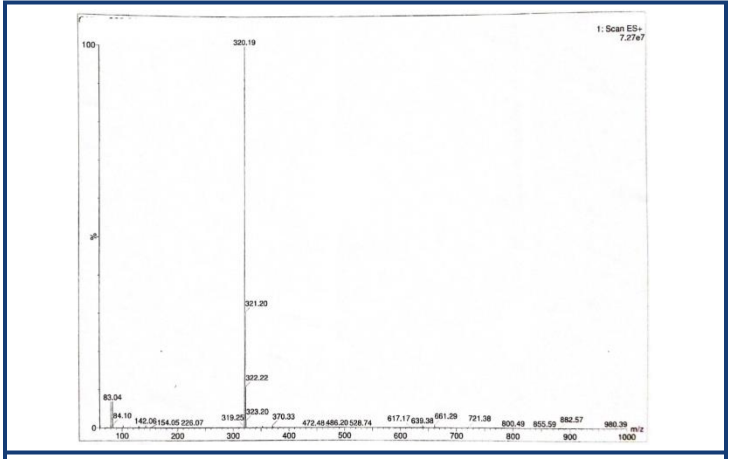

Mass spectrum of Compound 7j

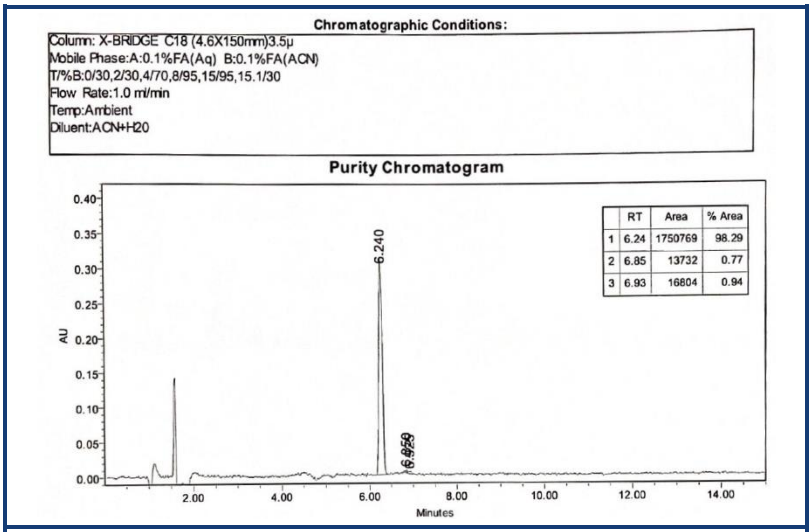

	RT	Area	% Area
1	6.24	1750769	98.29
2	6.85	13732	0.77
3	6.93	16804	0.94

HPLC Chromatogram of Compound 7j

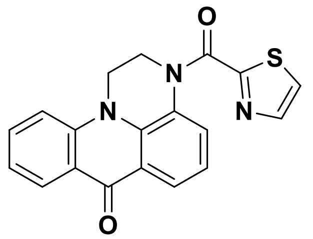

Structure of Compound 7j

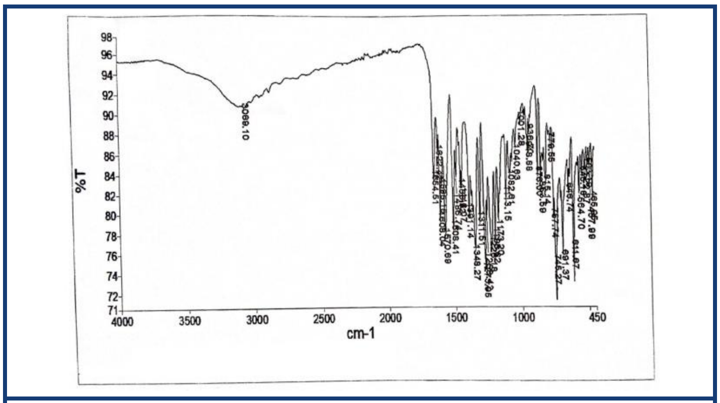

FTIR spectrum of Compound 7k

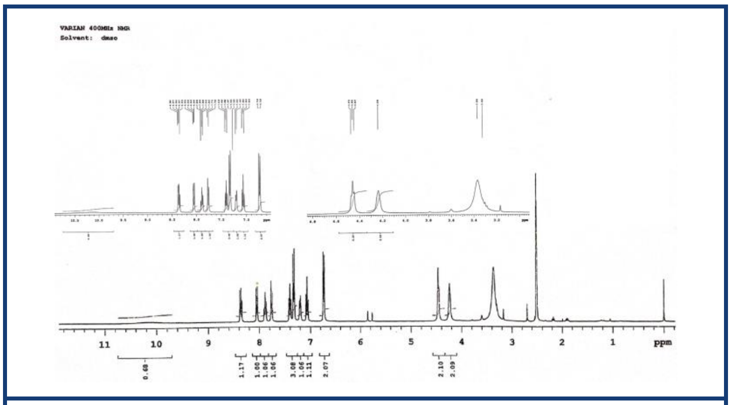

^{1}H NMR spectrum of Compound 7k

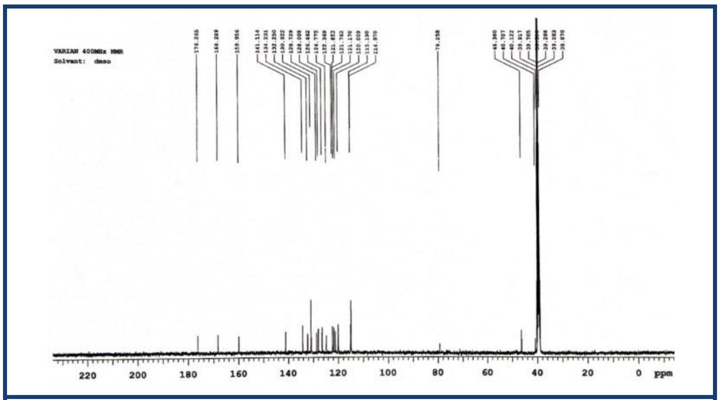

^{13}C NMR spectrum of Compound 7k

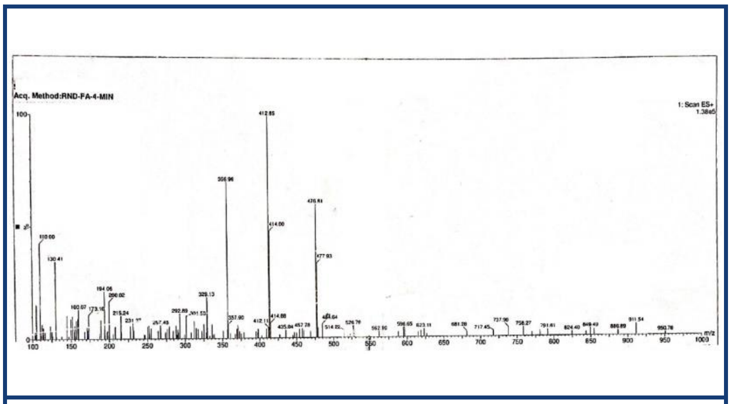

Mass spectrum of Compound 7k

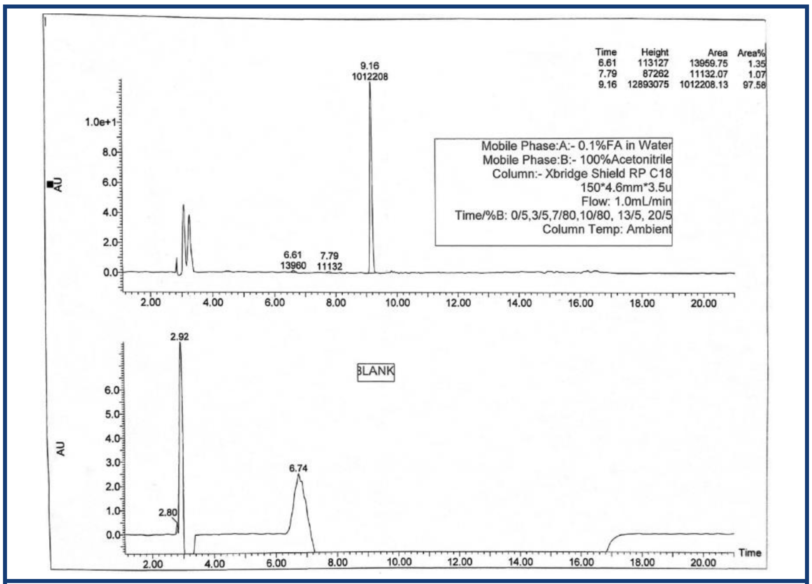

HPLC Chromatogram of Compound 7k

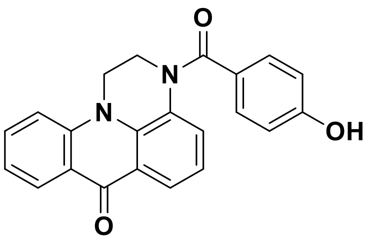

Structure of Compound 7k

Analysis of Compound 7l:

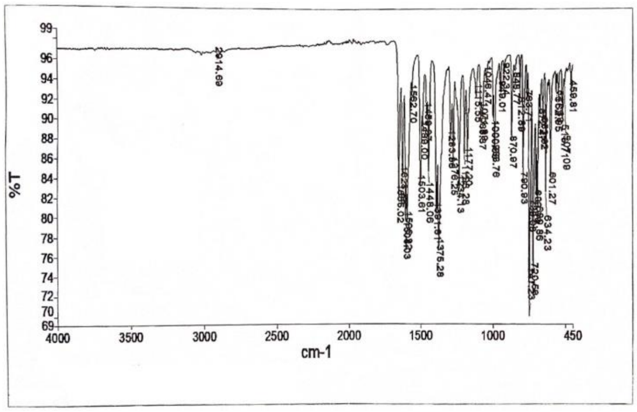

FTIR spectrum of Compound 7l

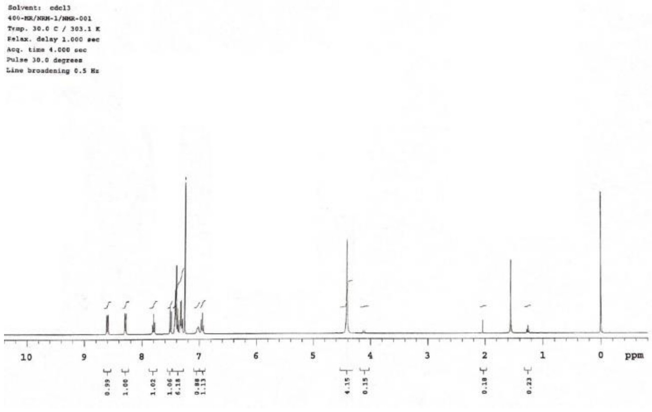

^{1}H NMR spectrum of Compound 7l

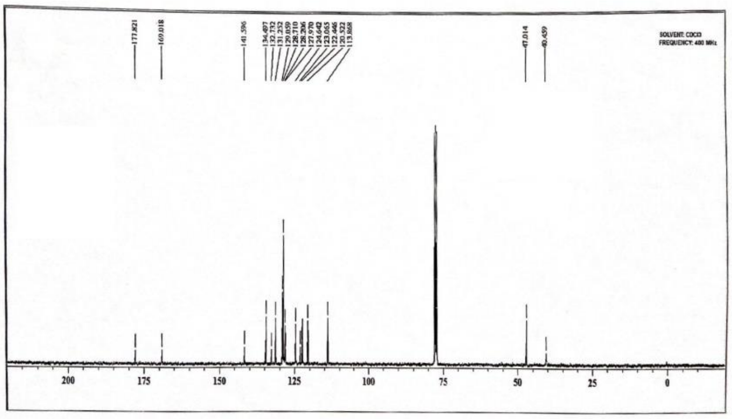

^{13}C NMR spectrum of Compound 7l

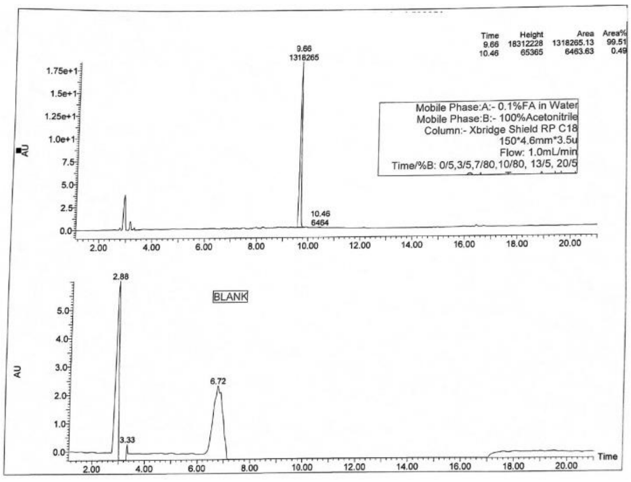

HPLC Chromatogram of Compound 7l

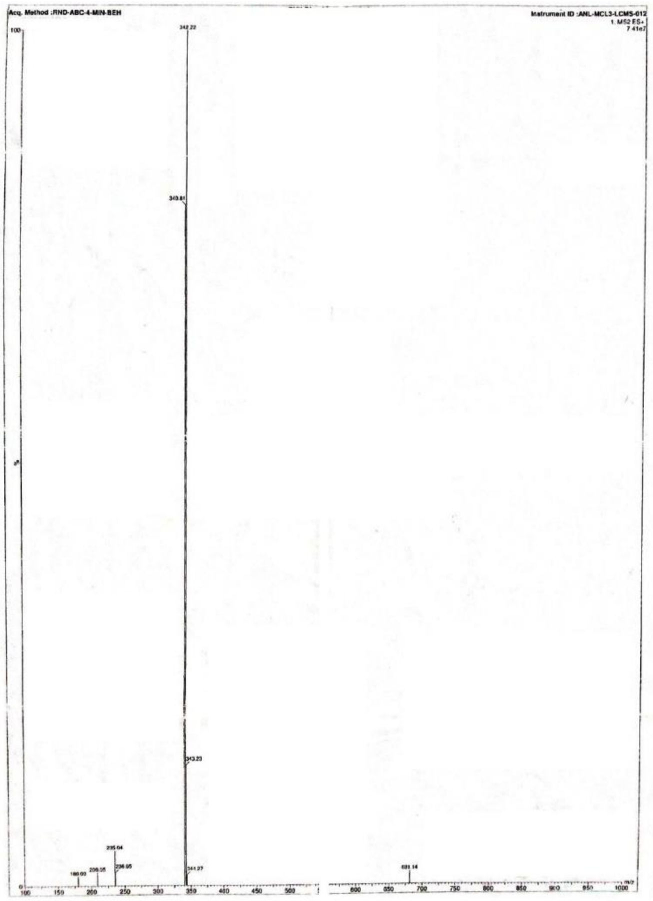

Mass spectrum of Compound 7l

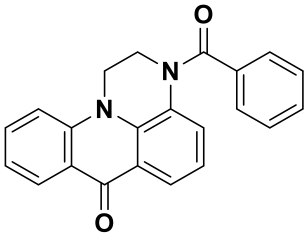

Structure of Compound 7l

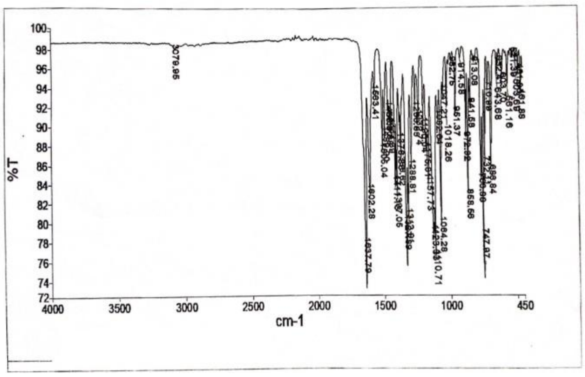

FTIR spectrum of Compound 7m

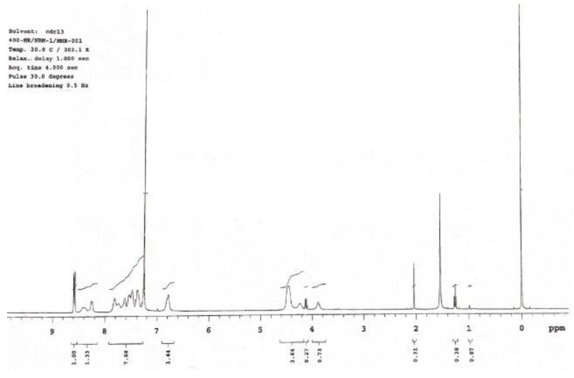

^{1}H NMR spectrum of Compound 7m

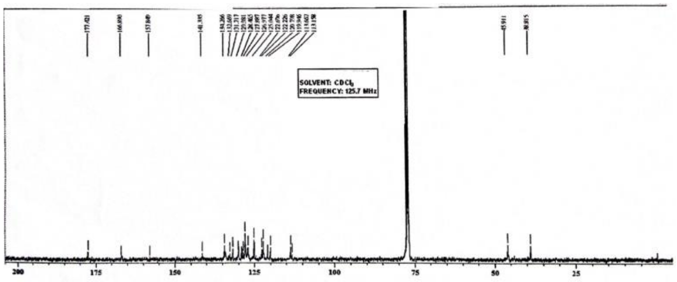

^{13}C NMR spectrum of Compound 7m

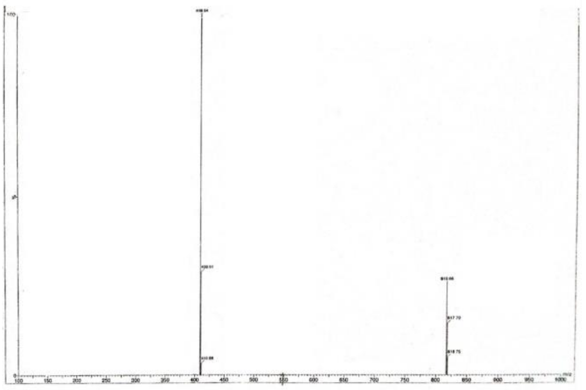

Mass spectrum of Compound 7m

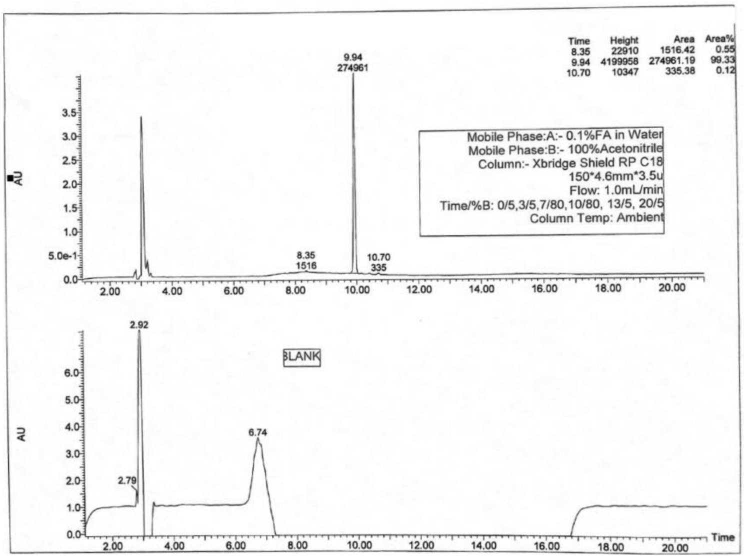

HPLC Chromatogram of Compound 7m

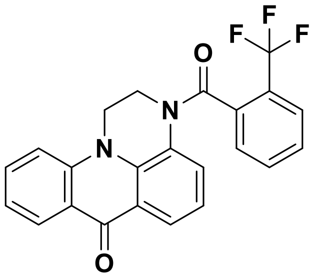

Structure of Compound 7m

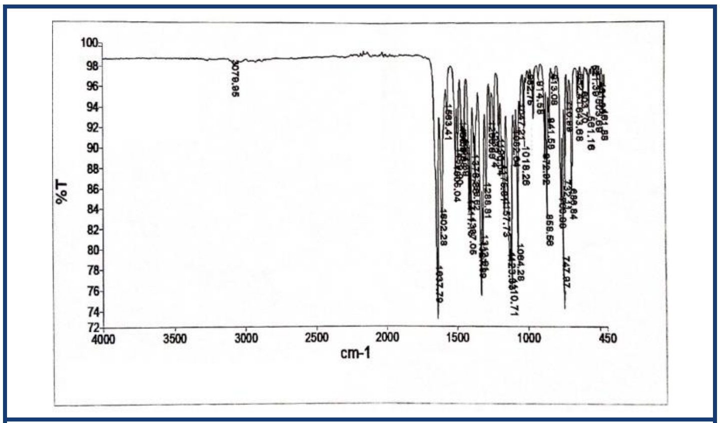

FTIR spectrum of Compound 7n

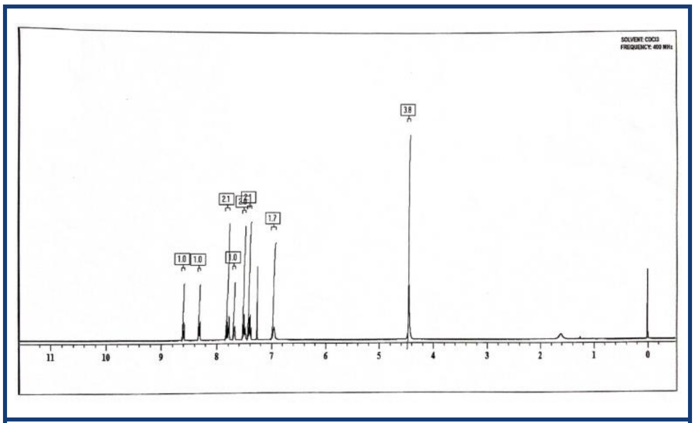

^{1}H NMR spectrum of Compound 7n

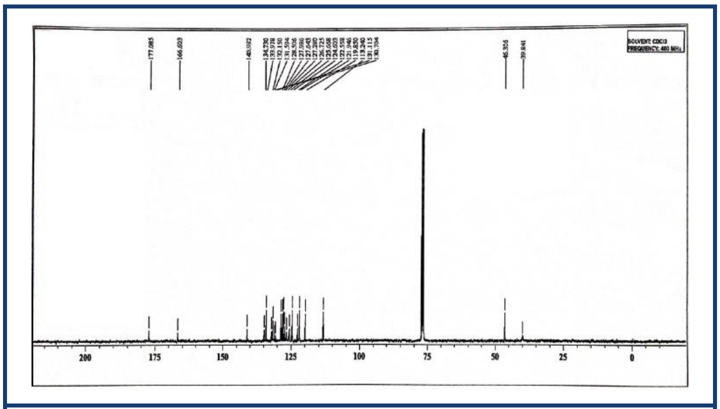

^{13}C NMR spectrum of Compound 7n

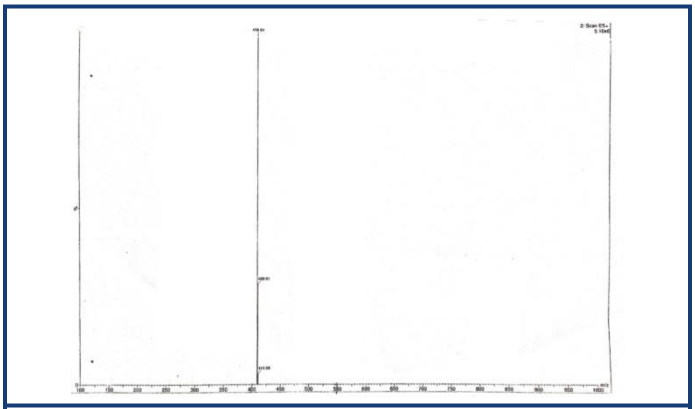

Mass spectrum of Compound 7n

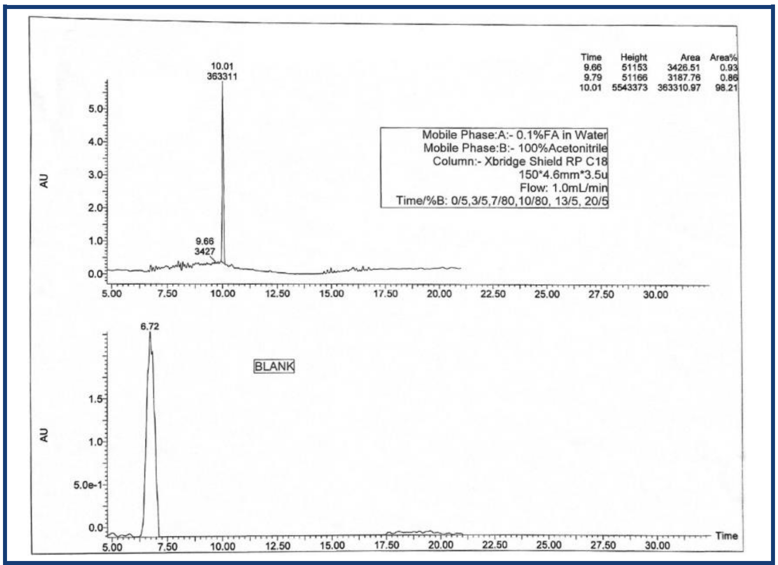

HPLC Chromatogram of Compound 7n

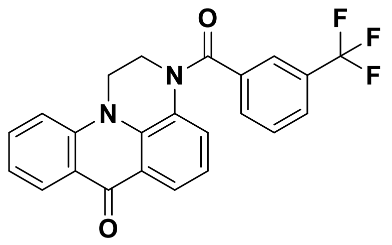

Structure of Compound 7n

Analysis of Compound 7o:

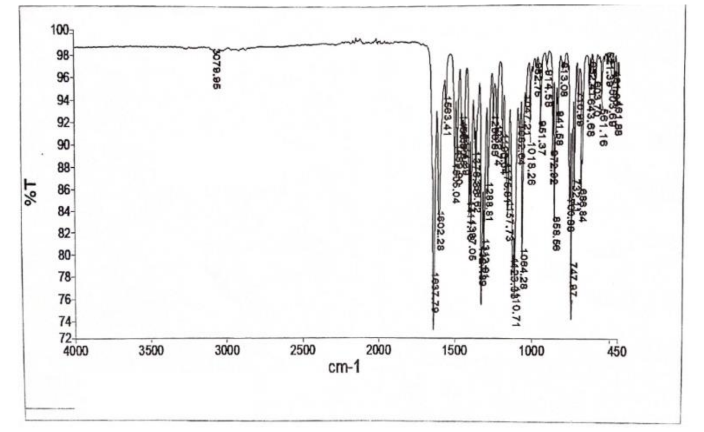

FTIR spectrum of Compound 7o

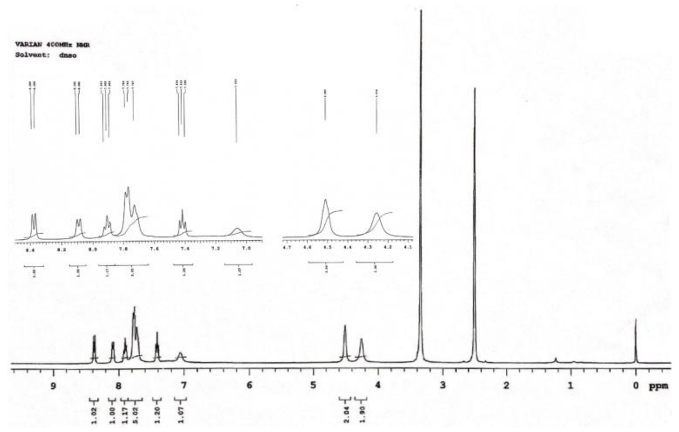

^{1}H NMR spectrum of Compound 7o

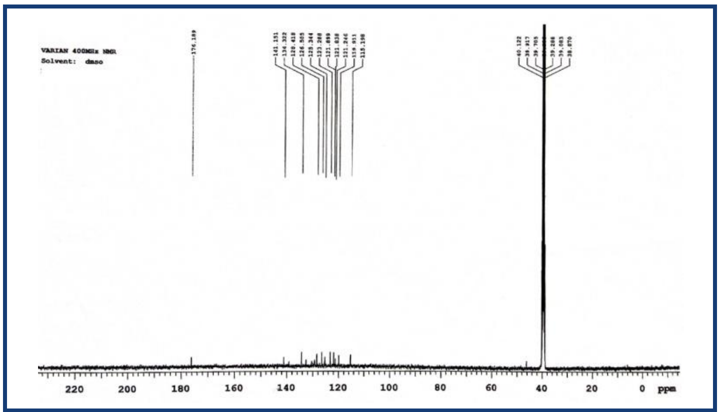

^{13}C NMR spectrum of Compound 7o

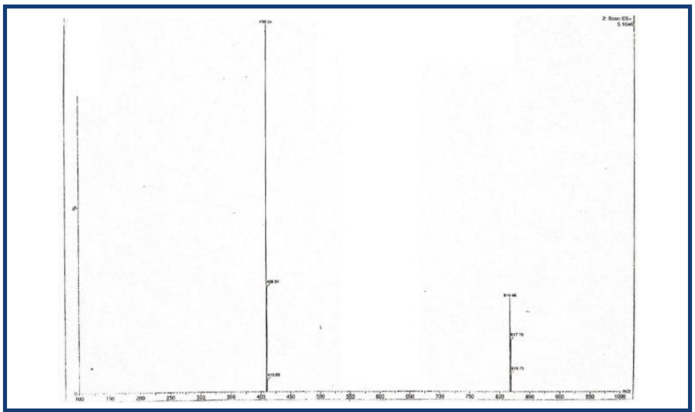

Mass spectrum of Compound 7o

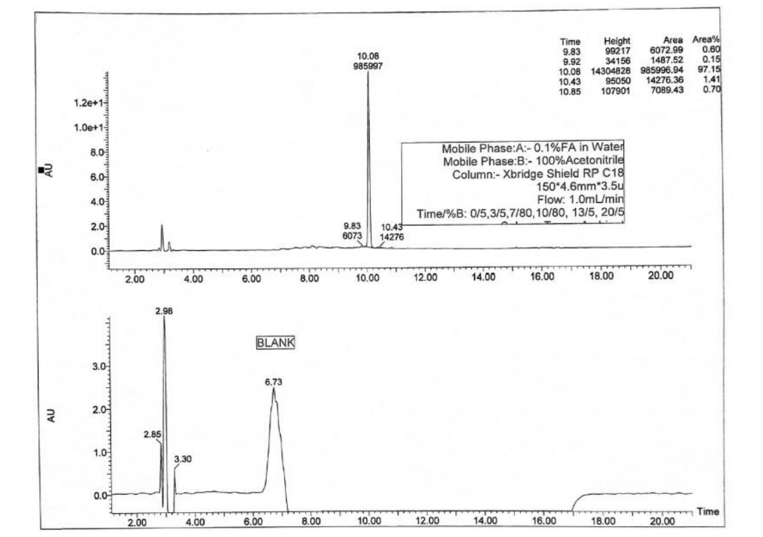

HPLC Chromatogram of Compound 7o

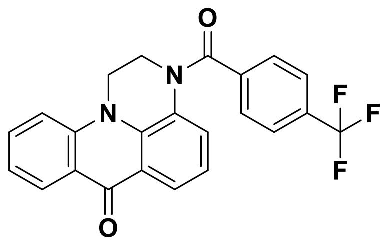

Structure of Compound 7o

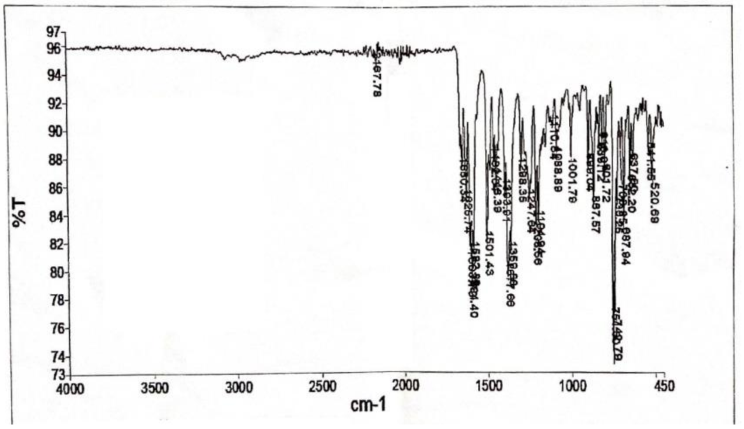

FTIR spectrum of Compound 7p

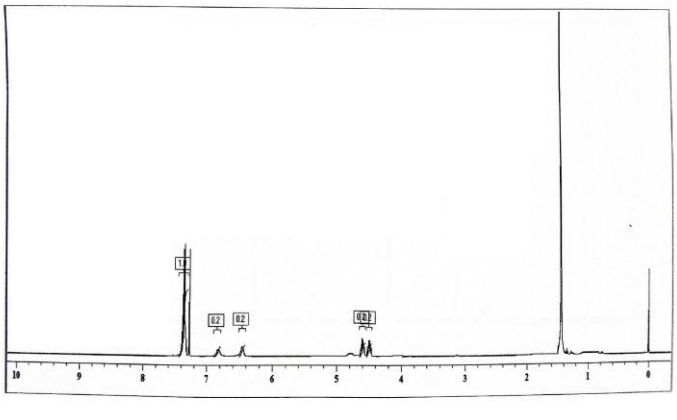

^{1}H NMR spectrum of Compound 7p

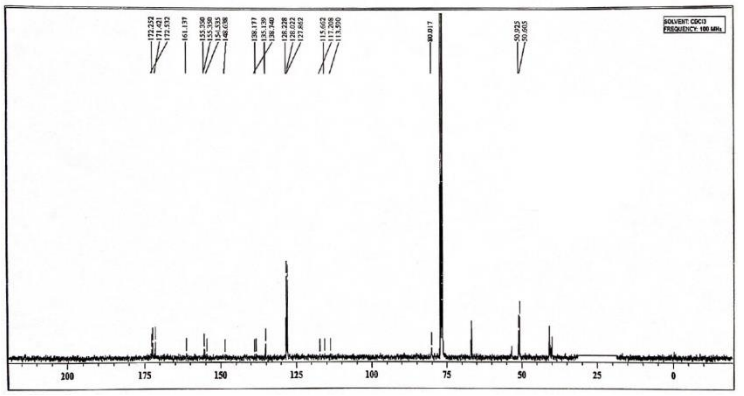

^{13}C NMR spectrum of Compound 7p

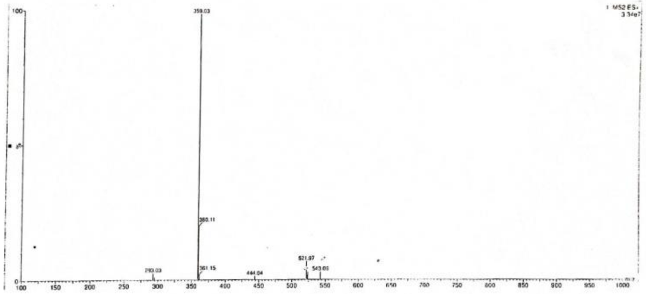

Mass spectrum of Compound 7p

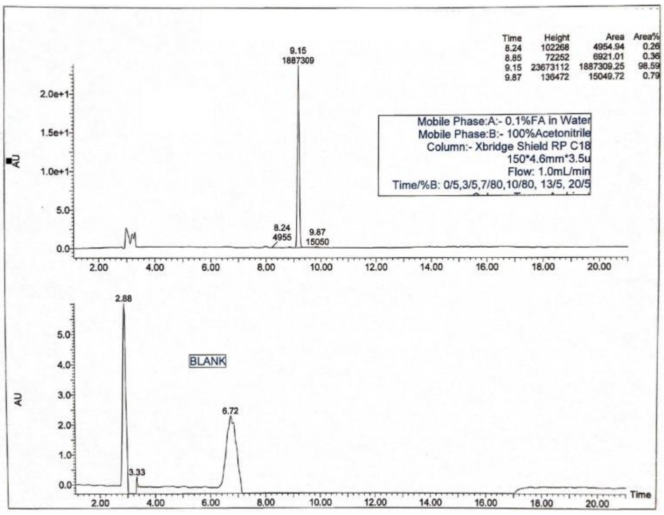

HPLC Chromatogram of Compound 7p

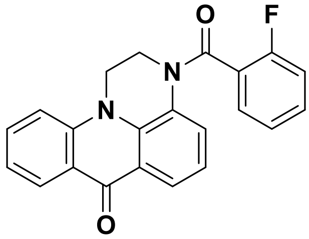

Structure of Compound 7p

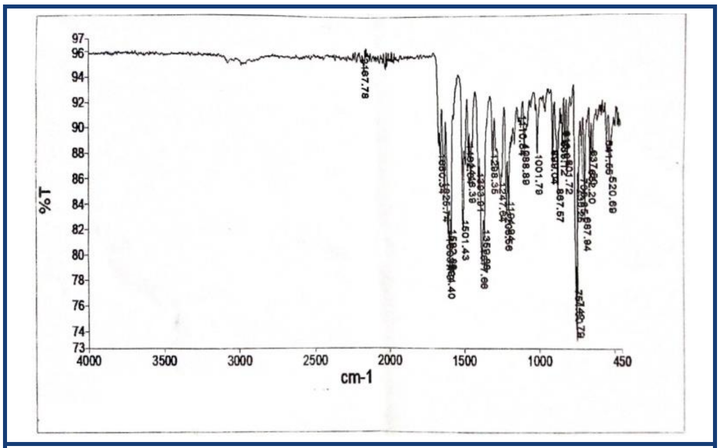

FTIR spectrum of Compound 7q

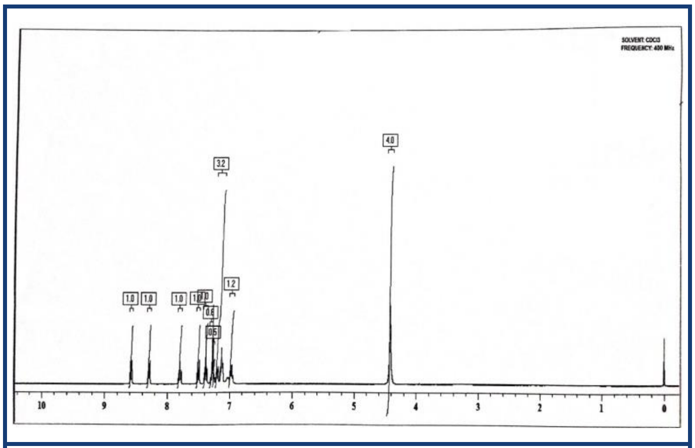

^{1}H NMR spectrum of Compound 7q

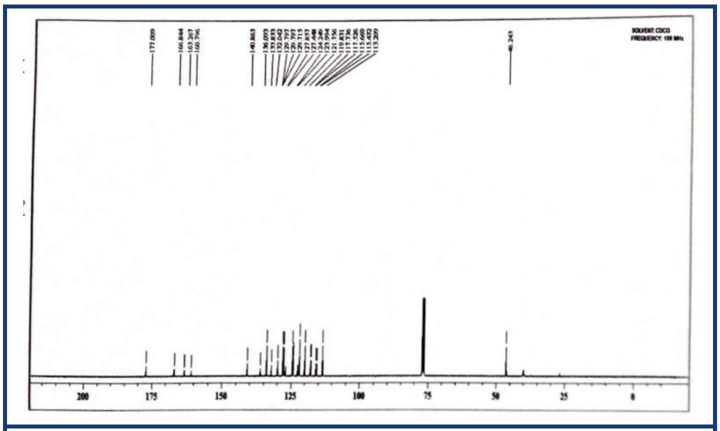

^{13}C NMR spectrum of Compound 7q

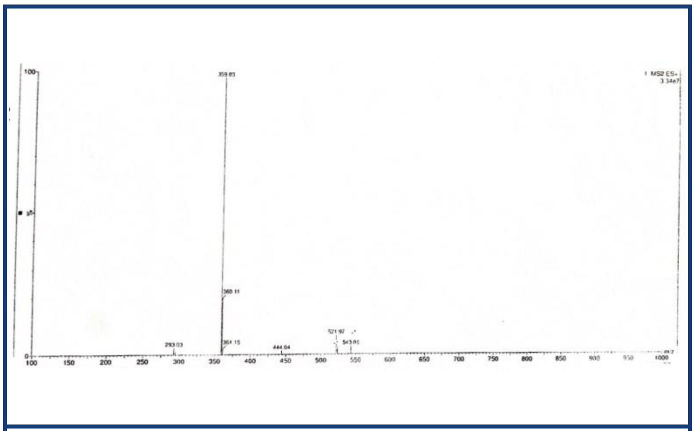

Mass spectrum of Compound 7q

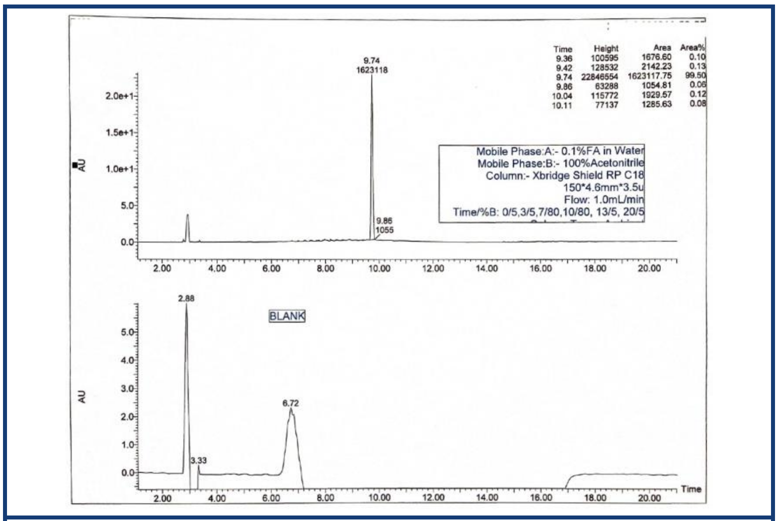

HPLC Chromatogram of Compound 7q

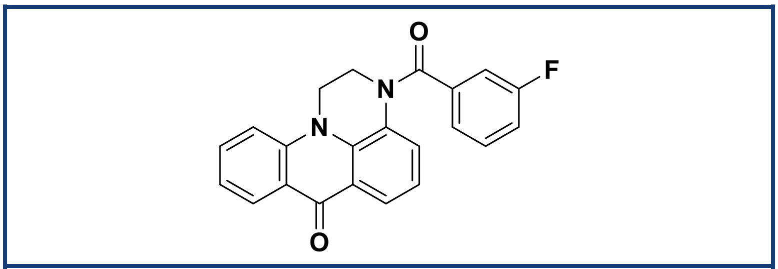

Structure of Compound 7q

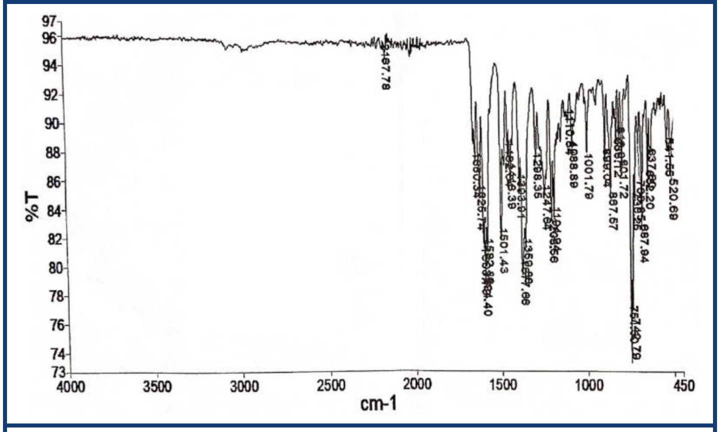

FTIR spectrum of Compound 7r

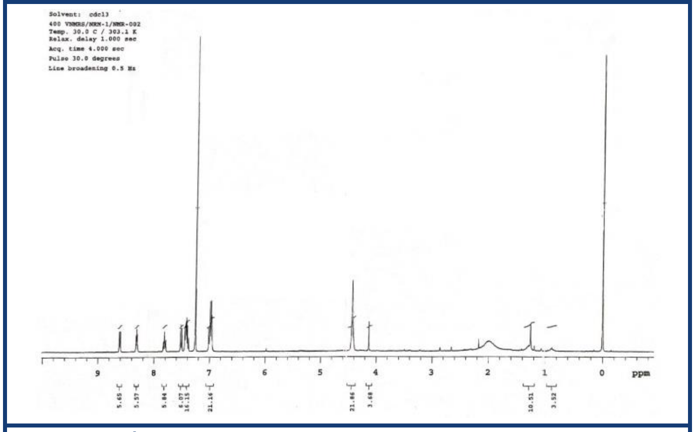

^{1}H NMR spectrum of Compound 7r

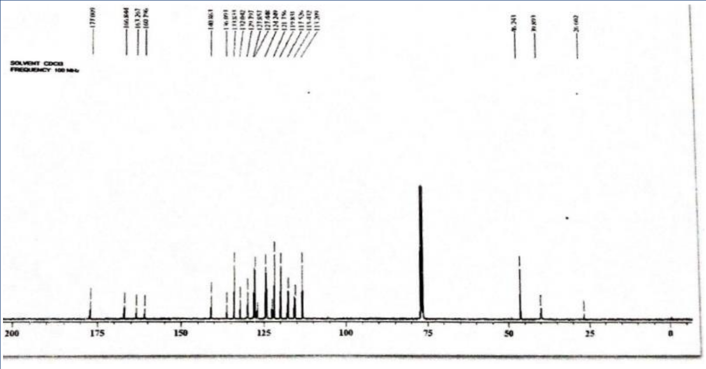

^{13}C NMR spectrum of Compound 7r

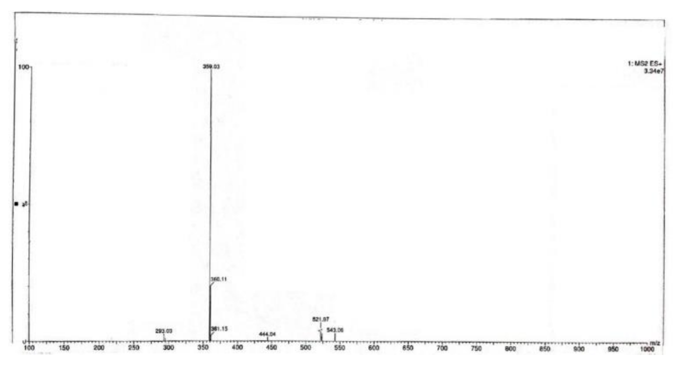

Mass spectrum of Compound 7r

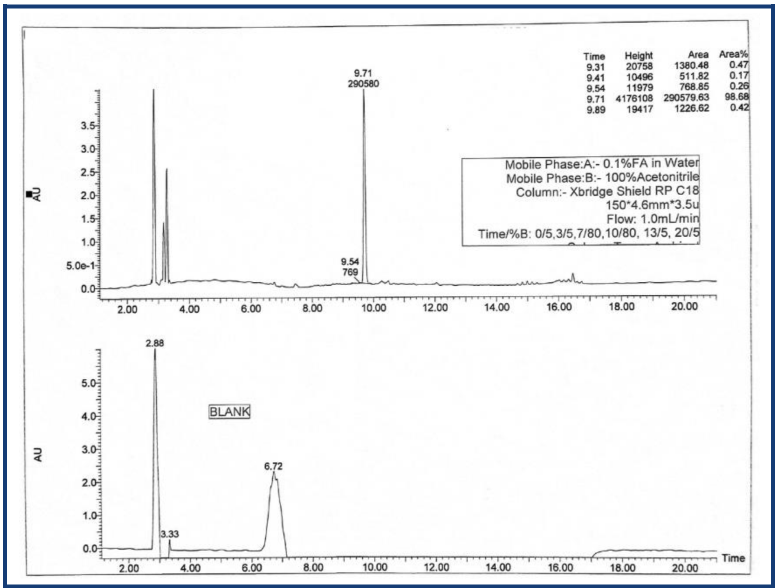

HPLC Chromatogram of Compound 7r

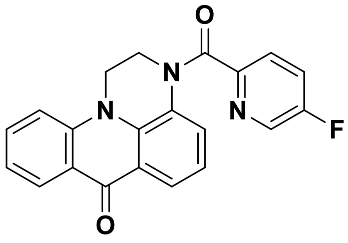

Structure of Compound 7r

Analysis of Compound 7s:

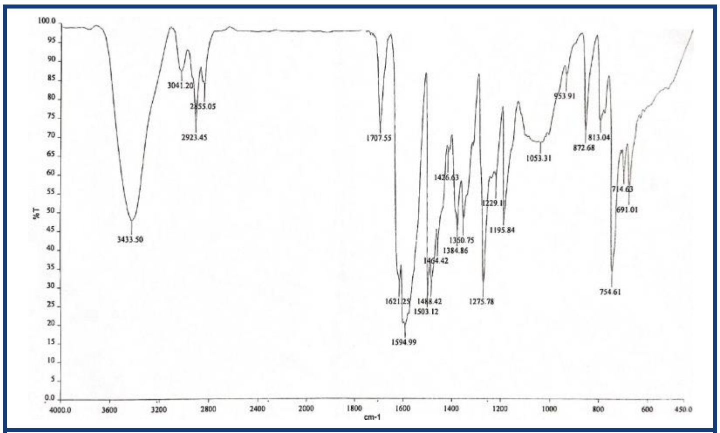

FTIR spectrum of Compound 7s

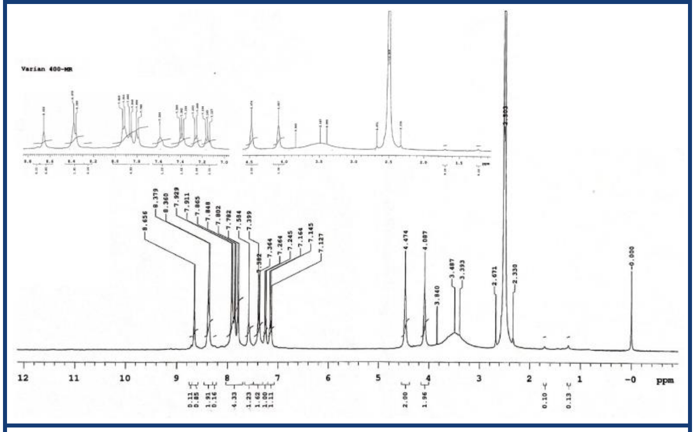

^{1}H NMR spectrum of Compound 7s

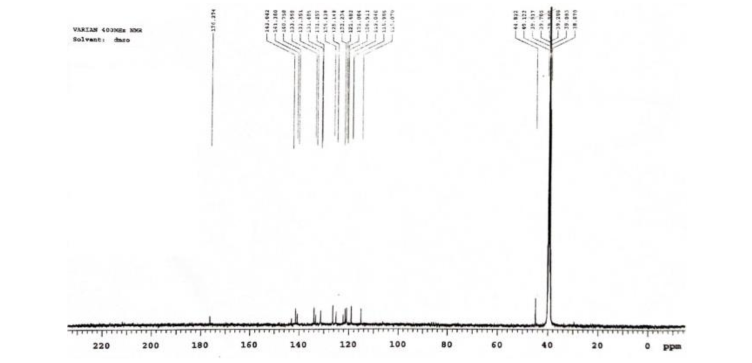

^{13}C NMR spectrum of Compound 7s

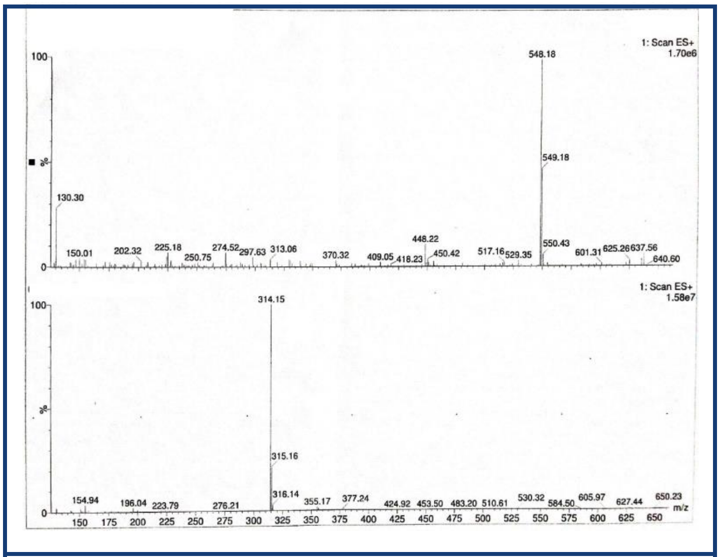

Mass spectrum of Compound 7s

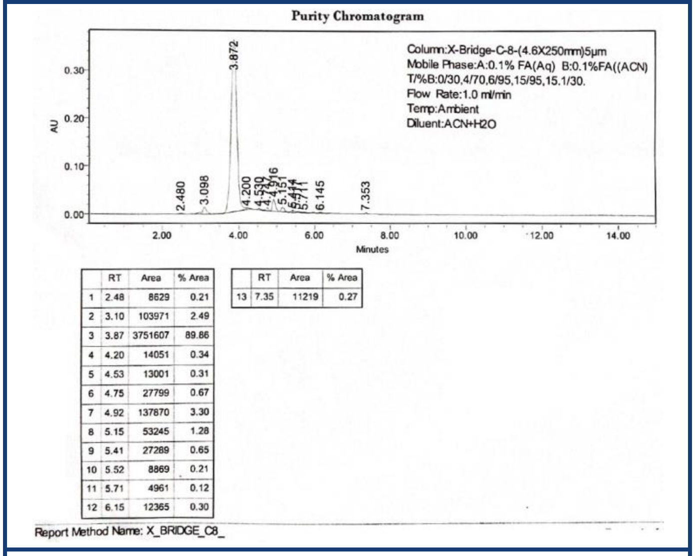

	RT	Area	% Area
1	2.48	8629	0.21
2	3.10	103971	2.49
3	3.87	3751607	89.86
4	4.20	14051	0.34
5	4.53	13001	0.31
6	4.75	27799	0.67
7	4.92	137870	3.30
8	5.15	53245	1.28
9	5.41	27289	0.65
10	5.52	8869	0.21
11	5.71	4961	0.12
12	6.15	12365	0.30
13	7.35	11219	0.27

HPLC Chromatogram of Compound 7s

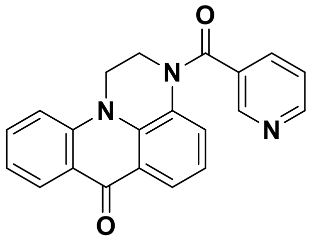

Structure of Compound 7s
